# Biocatalytic Regioselective C‐Formylation of Resorcinol Derivatives

**DOI:** 10.1002/anie.202519387

**Published:** 2026-01-29

**Authors:** Lilla Gal, Suresh Rohan, Anna Żądło‐Dobrowolska, Bianca Hilweg, Judith Müller, Kai Tittmann, Wolfgang Kroutil

**Affiliations:** ^1^ Institute of Chemistry University of Graz Graz Austria; ^2^ Department of Molecular Enzymology Georg‐August University Göttingen Göttingen Germany; ^3^ Field of Excellence Biohealth BioTech Med Graz University of Graz Austria

**Keywords:** acyltransferases, biocatalysis, biocatalytic formylation, resorcinol derivatives, X‐ray structure elucidation

## Abstract

Although aromatic formylation reactions are highly valuable from a synthetic perspective, a biocatalytic version has not yet been reported. Here, the cofactor‐independent multimeric three‐component acyltransferase from *Chromobacterium sphagni* (*Cs*ATase) was identified to enable the nonnatural promiscuous regioselective C‐formylation of polyphenolic substrates, especially resorcinol derivatives, and thus extending the reaction scope of acyltransferases. Formylation of 4‐ and 5‐substituted resorcinol derivatives gave access to regioselectively mono‐formylated products with up to 99% conversion and up to 74% isolated yield. Formylation of phloroglucinol led to the di‐formylated product with 99% conversion, outperforming chemical methods. Structural analysis of *Cs*ATase by X‐ray crystallography provided insights into its active site.

## Graphical abstract

An acyltransferase from *Chromobacterium sphagni* (*Cs*ATase) was identified that catalyzes the regioselective formylation of resorcinol substrates. The formylation of substituted resorcinol derivatives yielded mono‐formylated products with up to 99% conversion and up to 74% isolated yield. The structure of *Cs*ATase was elucidated by X‐ray crystallography, providing insight into its active site.

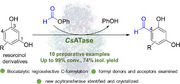

## 1 Introduction

**SCHEME 1:**
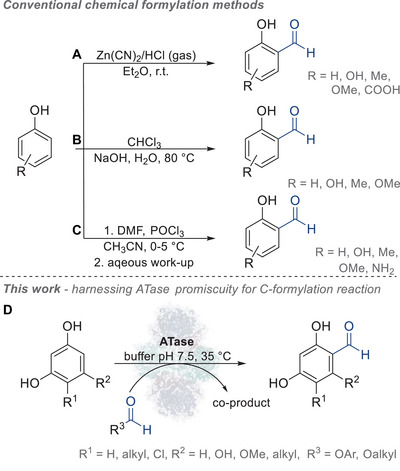
Selected established methods for chemical C‐formylation of phenols and the unprecedent biocatalytic C‐formylation of resorcinol. (A) Gattermann–Adams formylation of electron‐rich aromatic compounds using zinc cyanide, (B) base‐promoted Reimer–Tiemann formylation of phenols with chloroform, (C) Vilsmeyer‐Haack formylation of electron‐rich aromatics using *N,N*‐dimethylformamide and phosphorus oxychloride, (D) acyltransferase (ATase)‐catalyzed formylation of electron‐rich aromatic compounds.

Aromatic aldehydes are ubiquitous targets and intermediates in organic synthesis due to the versatility of the formyl group, which can be easily transformed into a variety of functional groups, including alcohols, carboxylic acids, and esters [1, 2, 3, 4, 5]. Although several methods have been developed for the formylation of aromatic compounds [6], the formylation of phenols through C─C bond‐forming reactions has remained a significant challenge throughout the history of organic chemistry [7]. Classical methods involve direct formylation reagents that introduce the formyl group in a single step. For instance, the Gattermann reaction [8, 9, 10] was subsequently simplified by Adams, who replaced the hazardous combination of HCN and ZnCl_2_ with the more stable and safer Zn(CN)_2_ (Scheme 1A) [11]. Around the same time, Reimer and Tiemann demonstrated that phenols can be formylated using chloroform in an aqueous alkali medium (Scheme 1B) [12, 13, 14]. The formylation of a wide range of substrates, particularly electron‐rich aromatics, can be efficiently achieved using the adduct formed between *N,N*‐dimethylformamide [15] (DMF) and phosphoryl chloride (POCl_3_), in a process known as the Vilsmeier–Haack reaction (Scheme 1C) [16, 17]. Other methods involve multi‐step C─C bond‐forming processes, in which functionalized intermediates are generated and subsequently oxidized to yield the target aldehydes. Prominent examples of this approach include the Mannich‐type Duff formylation [18, 19] and the Casiraghi approach utilizing iminium intermediates to achieve formylation through the reaction between phenols and paraformaldehyde [20, 21]. Other formylating agents have been developed as well, such as formyl fluoride [22, 23], dichloromethyl methyl ether [24, 25], formamide derivatives in the presence of Lewis acids [26], and organo‐metallic approaches, such as utilizing magnesium phenoxides and formaldehyde in the Casnati–Skattebøl reaction [21]. In contrast to these many chemical methods, a biocatalytic method [27, 28, 29, 30, 31, 32, 33, 34] for the C‐formylation of phenols via C─C bond formation has remained elusive. Nevertheless, significant advances have been made in biocatalytic C─C bond‐forming reactions [35, 36, 37, 38, 39, 40, 41, 42, 43], employing both wild‐type enzymes and engineered variants. These include classical transformations such as aldol reactions [44, 45, 46, 47, 48, 49, 50, 51], acyloin condensations [52, 53], carboligation [54, 55], and cyanohydrin formations [56, 57, 58], as well as transformations such as alkylation [59, 60, 61], acylation [62, 63, 64], oxidative C–C coupling [65, 66, 67, 68], cyclization [69, 70], and carbene transfer [71, 72, 73, 74, 75].

Formylation/formyl transfer involving enzymes has in general been described to occur at nitrogen atoms but not at a carbon: beyond the biosynthetic pathways, such as the initiation of protein synthesis via *N*‐formylmethionine, mediated by methionyl‐tRNA formyltransferase [76], or in the biosynthesis of purine nucleotides by transformylases [77], only a few examples of nonnatural enzymatic *N*‐formylation have been described (Figure S40), including transformations catalyzed by *Candida antarctica* lipase [78], chitobiose deacetylase [79], and *N*‐formyltransferase [80]. Formaldehyde has been described as a substrate in a C–C‐formation reaction in the artificial formolase‐catalyzed carboligation of three molecules of formaldehyde to form dihydroxyacetone [81, 82].

Herein, we report an unprecedented biocatalytic C‐formylation of resorcinol derivatives, offering an alternative to chemical methods and expanding the catalytic repertoire of acyltransferases acting on phenolic substrates (Scheme 1D) [62, 63, 64, 83].

## 2 Results and Discussion

### 2.1 Search for Promiscuous Activity

As C‐formylation of phenols has not been described in metabolic pathways yet, we started to search for enzymes transferring acyl groups without the need for activation, for example, as SCoA derivative. Such an SCoA‐free activity has been described in the disproportionation of monoacetylphloroglucinol to diacetylphloroglucinol (DAPG) and phloroglucinol catalyzed by a multicomponent acyltransferase (ATase) consisting of three subunits (*PhlA, PhlC*, and *PhlB*) [84, 85], which was later exploited in acylation reactions [62, 63, 64, 83]. Previous studies have shown that production of a functional ATase requires expression of the complete *PhlACB* operon, although the catalytic activity is primarily attributed to the *PhlC* subunit, a member of the thiolase superfamily. Within this operon, the *PhlA* subunit shows structural similarity to hydroxymethylglutaryl‐CoA synthases and β‐oxoacyl–(acyl‐carrier‐protein) synthases, whereas *PhlB* resembles a Zn‐ribbon–domain OB‐fold (oligonucleotide/oligosaccharide‐binding) protein [86]. Consequently, the gene cluster of such an ATase (*PhlACB* gene cluster sequence of *Pseudomonas* *protegens*) was taken as a template for a BLAST search to identify homologous subunits in other organisms to set up an enzyme library potentially capable of catalyzing the formylation of phenolic substrates in an aqueous environment. A search of the NCBI database yielded over 100 acyltransferase sequences, from which seven were arbitrarily selected (Table 1). Each sequence originated from a different species of prokaryote. Average sequence identity to the template for the three subunits ranged from 33%—the lowest, found in the *Thermofilum* *pendens* enzyme—to 96%, observed in the enzyme from *Pseudomonas piscis* (Table S3). When just considering the transfer‐active subunit *Phl*C, sequence identity varied between 30% and 98% (Table 1). Notably, the *PhlACB* gene cluster from *Chromobacterium sphagni*, which shares 81% average sequence identity with the template, was included, as the activity of the DAPG biosynthetic cluster in the *Chromobacterium* genus was reported very recently [87]. The seven multimeric homologous enzymes and the template were successfully produced in *Escherichia coli* (Figure S3) and subsequently tested as cell‐free extract (CFE) preparation for their ability to catalyze the formylation of the model substrate resorcinol (**1a**) using phenyl formate (**2a**) as formyl donor at 5 mM substrate concentration of **1a** and 10 equivalents of donor. Gratifying, four of the eight enzymes tested showed formylation activity (Table 1). In addition to *Pp*ATase, two *Pseudomonas*‐derived enzymes, *Pk*ATase and *Pt*ATase, demonstrated catalytic activity, while the CFE preparation with *Ppi*ATase (98% sequence identity for *PhlC*, but just 92% coverage) showed no detectable activity, which might be attributed to the missing of 28 residues at the *N*‐terminus of the *PhlC*‐subunit (Figure S33). *Cs*ATase also exhibited promising activity, which was particularly interesting as it displayed significantly lower expression levels compared to *Pp*ATaseCH (Figure S3). To enhance the expression level of *Cs*ATase, the expression construct was engineered by introducing linkers, prefixes, and suffixes between the *PhlC* and *PhlB* subunits in a comparable fashion as described for *Pp*ATaseCH (Figure S1). This modification resulted in clearly improved expression levels (Figure S4).

**TABLE 1:** Initial test on the formylation of **1a** using different ATases.

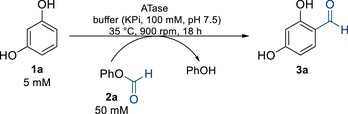			
Enzyme	Origin	*PhlC* Seq. Id. [%]^a^	Product formation^b^
*Pp*ATaseCH	*Pseudomonas protegens*	100	++
*Pt*ATase	*Pseudomonas thivervalensis*	94	+
*Pk*ATase	*Pseudomonas kilonensis*	87	+
*Ppi*ATase	*Pseudomonas piscis*	98	—
*Cs*ATase	*Chromobacterium sphagni*	91	++
*Bt*ATase	*Brenneria tiliae*	73	—
*Vb*ATase	*Vibrio aerogenes*	63	—
*Tp*ATase	*Thermofilum pendens*	30	—

^a^ Sequence identity in comparison to the template *Pp*ATase for the subunit *PhlC*, which is the subunit bearing the catalytic site for acyltransfer.

^b^ ++ means ≥50% product formation; +: 0–49%; ‐: below detection limit. Reactions were performed in 1.5 mL polypropylene tubes at 35°C, 900 rpm for 18 h, in 1 mL reaction volume, using 5 mM of resorcinol **1a**, 50 mM of **2a**, 60 mU/mL of CFE of ATases in KPi buffer (100 mM, pH 7.5).

### 2.2 Scope of Formyl Donors and Reaction Engineering

**FIGURE 1:**
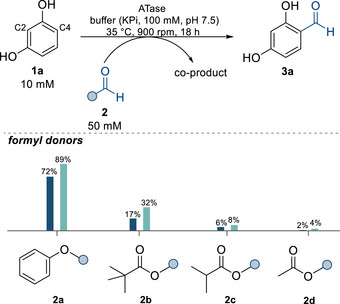
Conversions for accepted formyl donors for the formylation of **1a**. Conversions are shown in blue for *Pp*ATaseCH and in cyan for *Cs*ATase. Reactions were performed in 1.5 mL polypropylene tubes at 35°C, 900 rpm for 18 h, in 1 mL reaction volume, using 10 mM of **1a**, 50 mM of formyl donor, and 60 mU/mL of CFE of ATases (for specific activities see Table S10) in KPi buffer (100 mM, pH 7.5).

In the next step, the scope of formyl donors was investigated for the two best‐performing enzymes, *Cs*ATase and *Pp*ATaseCH, testing mixed anhydrides (**2b‐2d**) (Figure 1), amides (**2e‐2f**), and aliphatic esters (**2g**‐**2k**) (Figure S9). Among the tested donors, phenyl formate (**2a**) was the most effective one in the ATase‐catalyzed formylation of **1a** (10 mM) (Figure 1). *Cs*ATase reached 89% conversion, outperforming here *Pp*ATaseCH (72%). Other donors, such as **2b–2d**, resulted in lower conversions compared to ester **2a**. Nevertheless, the mixed anhydride from pivalic and formic acid **2b** was also accepted reasonably well by *Cs*ATase, leading to a conversion of 32% under the conditions used. One could expect that for the mixed anhydrides both acyl parts could be transformed, leading to a product mixture. This was indeed observed for acetic formic anhydride (**2d**) which led to both formylation as the main reaction and to a smaller extent acetylation (<2% conversion with a ratio between formyl:acetyl product 2:1).

It is worth mentioning that the mixed anhydrides underwent very fast spontaneous hydrolysis in the aqueous environment, which is likely one reason for the limited product formation in these cases. The other formyl donors tested (**2e**‐**2k**) did not lead to detectable product formation. Consequently, phenyl formate (**2a**) was the formyl donor of choice for the further experiments. Notably, using **2a** as donor, exclusively mono‐formylation of resorcinol at position C4 was observed, while, for example, with Reimer–Tiemann conditions formylation leads to products with the carbaldehyde moiety at C2 and C4 (C2:C4 ∼ 2:1) [88].

As the formyl donor **2a** also undergoes spontaneous hydrolysis like the mixed anhydrides but slower, its spontaneous degradation was followed over time (Figure S8). The results indicated that in case the donor is added at the beginning of the reaction at 50 mM, almost 90% were hydrolyzed after 80 min. This suggests that either the reaction should be performed for a short time (ideally 1–2 h) or the donor may be added continuously. For the small‐scale experiments, we decided to adjust the reaction conditions in a way that the reaction time is only a few hours.

**FIGURE 2:**
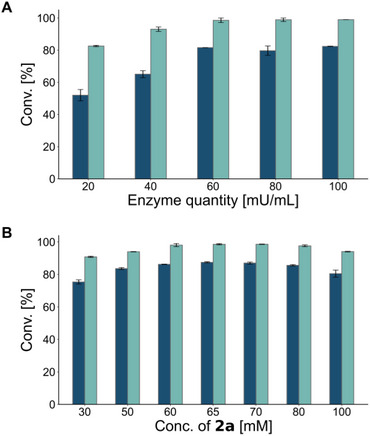
Formylation of **1a** using different quantities of acyltransferase (A) and various concentrations of **2a** (B). Conversions are shown in blue for *Pp*ATaseCH and in cyan for *Cs*ATase. Reactions were performed in 1.5 mL polypropylene tubes at 35°C, 900 rpm for 1 h, in 1 mL reaction volume, using 10 mM of **1a**, 30–100 mM of **2a** (50 mM for A), 20–100 mU/mL (60 mU/mL for B) of CFE of ATase in KPi buffer (100 mM, pH 7.5). Standard deviations were calculated from duplicate measurements.

Additionally, we were curious whether product **3a** would also serve as a substrate for the ATase potentially undergoing hydrolysis. This reverse hydrolysis reaction was investigated using *Cs*ATase and *Pp*ATaseCH at varying enzyme concentrations (Table S11). Indeed, **3a** underwent slow enzyme‐catalyzed deformylation when tested at 10 mM, leading to around 2% deformylated product within 18 h. Fortunately, the formylation (83 nmol min^−1^ mg^−1^_CFE_) proceeds approximately 900 times faster than the deformylation reaction (0.093 nmol min^−1^ mg^−1^_CFE_). This again supports that a short reaction time is preferable for the formylation reaction. To maximize conversion in the formylation of **1a** with **2a**, various enzyme loadings and formyl donor concentrations were evaluated. Increasing the amount of enzyme *Cs*ATase led to almost complete conversion (99%) of 10 mM **1a** within 1 h, whereas *Pp*ATaseCH never reached more than 82% (Figure 2A). Based on these results and prior optimization, 60 mU/mL was employed for subsequent reactions. Evaluating the impact of the concentration of the formyl donor **1a** on the reaction outcome (Figure 2B), a concentration of 70 mM was selected as the most suitable for the lab‐scale experiments. Again, the *Cs*ATase was clearly superior to the *Pp*ATase, as *Cs*ATase allowed to reach completion, while the conversions with *Pp*ATase were limited to a maximum of 87%.

### 2.3 Substrate Scope

**SCHEME 2:**
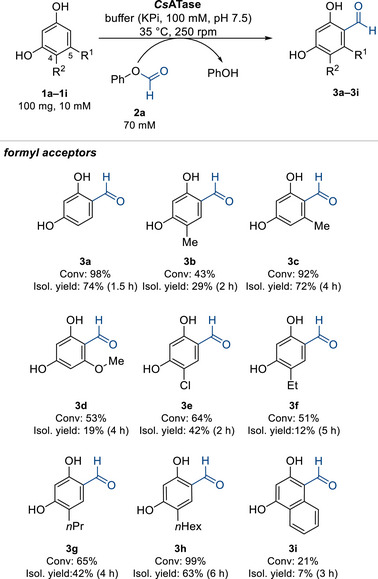
Scope of formyl acceptors in the biocatalytic C‐formylation of resorcinol derivatives. Reaction time for analytical reactions is 18 h, for preparative transformations see scheme. For solubility reasons 10% *v*/*v* DMSO was used in case of **1b**‐**1i**. Conversions were calculated based on calibration curves with the respective compounds and correspond also to HPLC yield due to a closed mass balance.

To tap the potential of substrates, a panel of phenolic compounds was evaluated using phenyl formate as the formyl donor and *Cs*ATase as the best biocatalyst. Substrates bearing two hydroxyl groups in 1,3‐position (resorcinol derivatives) were efficiently converted by *Cs*ATase (Scheme 2), whereas substrates bearing only one hydroxy group, like in guaiacol (**1m**) or phenol (**1k**), or having the hydroxy groups in 1,4‐position (**1l**) were not accepted (Figure S10). Substituents on resorcinol were accepted in positions 4 and 5, while position 2, as far as tested, was not accessible. For instance, the methyl group was accepted in position 4 (**1b**) as well as 5 (**1c**), whereby the latter led to higher conversion (92%) under the conditions tested. Also, chloro and methoxy groups were accepted as substituents (**1d**, **1e**). Increasing the size of the alkyl substituent in position 4 showed that with increasing length of the substituent from ethyl (**1f**) over *n*‐propyl (**1g**) to *n*‐hexyl (**1h**), also the conversion went up, reaching 99% for **1h**. Even a fused ring in position 4,5 was accepted (substrate **1i**), although the conversion was moderate (21%). However, increasing this substrate part even further, like to a flavone derivative (**1r**) (Figure S10), led to nonaccepted substrates; the same is true for a carboxylic acid moiety in position 4 (**1o**) or its ester in position 5 (**1p**). For all the resorcinol substrates accepted (Scheme 2), exclusively mono‐formylation was observed in a regioselective fashion.

To demonstrate the transformations on a semi‐preparative scale, the reactions were run for resorcinol derivatives **1a–i** using 100 mg of each substrate. Reaction progress was monitored over time, and each transformation was quenched upon conversion reaching a plateau under these conditions. The aldehydes **3a–i** were purified and isolated in moderate‐to‐high yields (7–74%, Scheme 2) and characterized by NMR (see Supporting information). No side products were detected; the diminished isolated yields in selected cases are due to challenging product isolation as a result of the similar polarity of product and co‐product (phenol).

**SCHEME 3:**
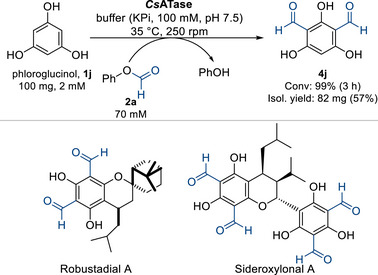
Biocatalytic di‐C‐formylation of phloroglucinol **1j** and bioactive molecules having **4j** as a precursor.

**FIGURE 3:**
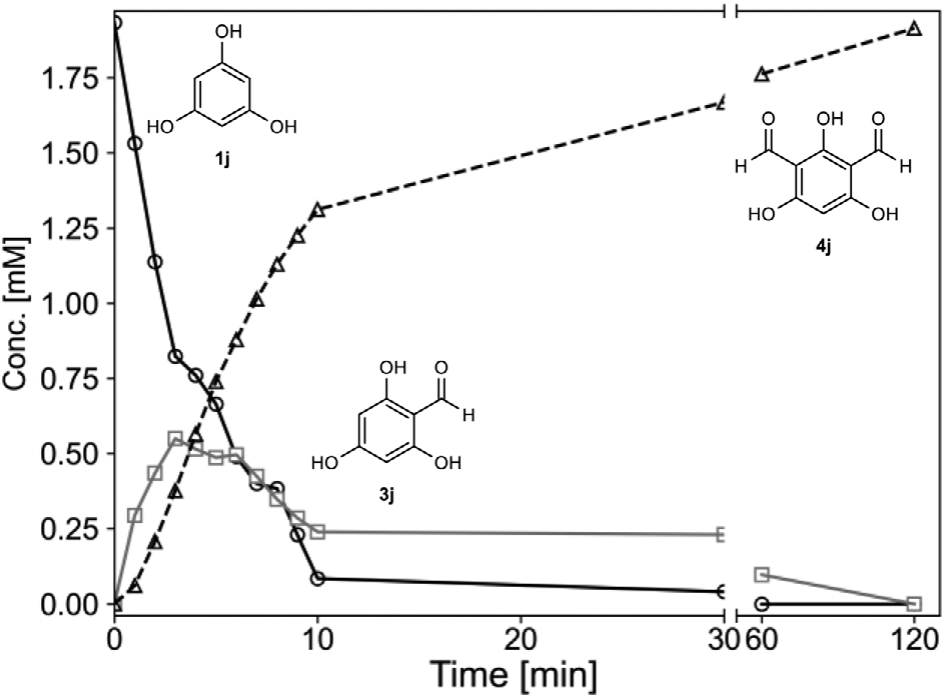
Time course of the *Cs*ATase catalyzed formylation of **1j**. The concentration of substrate **1j** (solid line, °), mono‐formylated product **3j** (solid lines, □), and di‐formylated product **4j** (dashed lines, △) were determined by HPLC using calibration curves. Reactions were performed in 1.5 mL polypropylene tubes at 35°C, 900 rpm for 2 h, in 1 mL reaction volume, using 2 mM of **1j**, 70 mM of **2a**, 60 mU/mL of CFE of ATase in KPi buffer (100 mM, pH 7.5).

Considering 1,3,5‐trihydroxybenzene (phloroglucinol **1j**) as substrate, mono‐, di‐ or tri‐formylation could be expected. Testing phloroglucinol (**1j**), indeed, di‐substitution was found giving **4j** without any detectable tri‐substitution (Scheme 3). Monitoring the progress of the formylation of **1j** over time revealed that the mono‐formylated intermediate **3j** was only detected in the initial phase of the formylation (Figure 3). A recent review [89] indicated that getting selectively only the di‐formylated phloroglucinol (2,4,6‐trihydroxyisophthalaldehyde **4j**) is challenging due to the strong deactivating effect of the formyl group on the aromatic ring [18].

It is worth noting that **4j** can be converted in a single step into the antimalarial robustadials A and B (Scheme 3) [90], and is a precursor in the synthesis of cancer chemopreventive euglobals [91], and antifouling sideroxylonals, which occur in plant leaves exhibiting significant antifeedant activity [92]. The latter is composed of two trihydroxyisophthalaldehyde molecules and has also shown notable antibacterial activity against Gram‐positive bacteria such as *Staphylococcus aureus* and *Bacillus subtilis* [93]. After some optimization, phloroglucinol **1j** was efficiently converted to the di‐formylated product **4j** with excellent conversion (>99%). Performing the di‐formylation of **1j** on a semi‐preparative scale (100 mg) yielded 82 mg of **4j** (57% isolated yield, for NMR see Supporting information). For comparison, chemical di‐formylation of **1j** has been reported either with Vilsmeier–Haack reagent (3 equiv. each of POCl_3_ and DMF) in 40% yield [90] and with Gattermann–Adams in 1.5% yield [94], thus the biocatalytic version is an interesting alternative.

### 2.4 Structure Elucidation and Mechanism

**FIGURE 4:**
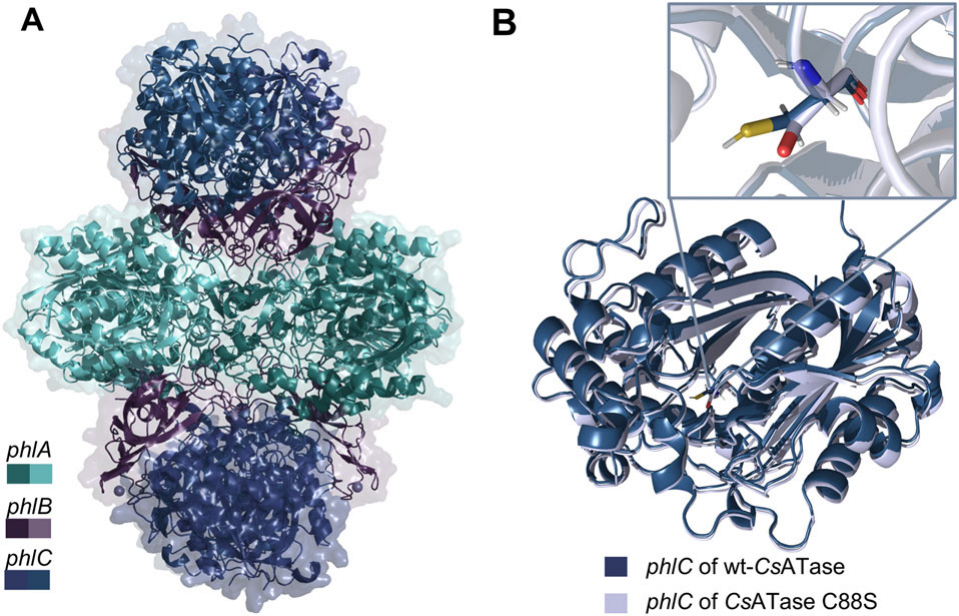
(A) Structure of the heterododecamer of *Cs*ATase (*PhlA* = cyan, *PhlB* = magenta, *PhlC* = blue) (PDB 9SKH). (B) Structural overlay of the *PhlC* wild‐type *Cs*ATase (blue) and the *PhlC*‐*Cs*ATase C88S variant (PDB 9SKM) (light purple).

The crystal structure of the newly identified acyltransferase from *C. sphagni* (*Cs*ATase) was determined by X‐ray crystallography at a resolution of 2.6 Å (Table S12). The structure was solved by molecular replacement involving automated and manual rebuilding (see Supporting information). *Cs*ATase is a multienzyme complex and consists of three individual subunits in multiple copies: *PhlA*, *PhlB*, and *PhlC*. The structure of the assembly is best described as a Phl(A_2_C_2_)_2_B_4_ heterododecamer (Figure 4A) similarly to the previously reported structure from *Pp*ATaseCH [86].

From the three subunits (*PhlA*, *PhlB*, and *PhlC*) it was expected that only *PhlC* is responsible for the formylation reaction, whereby a cysteine at position 88 (C88) in the *PhlC* subunit might be directly involved in formyl transfer. To test this assumption, C88 was exchanged for a serine by site‐selective mutagenesis. The variant was successfully expressed in a soluble form and crystallized. The structure of the C88S variant was solved at 1.8 Å resolution, whereby the structures of the C88S variant and the wild‐type aligned well with each other (Figure 4B), with an RMSD of 0.204 Å over 3397 residues. In a biocatalytic transformation test, the C88S variant did not show any formylation activity, confirming the essential catalytic function of C88 in the *PhlC* subunit.

As soaking experiments with substrates failed to yield well‐diffracting crystals, molecular docking experiments were performed to examine the binding mode and orientation of the substrate within the active site of the crystal structure of *Cs*ATase. Docking of the substrate into the structure resulted in poor substrate positioning, with the ligand being too distant from the catalytic Cys88. The reason for that was most likely that the crystal structure in the absence of a ligand captured the enzyme in an open conformation with a tryptophan (W211) displaced away from the active site, as observed in other studies [86]. Consequently, when simulating the closed conformation of W211, the binding poses observed were consistent with key mechanistic steps of a previously proposed reaction mechanism for acylation, based on quantum chemical calculations [95].

**FIGURE 5:**
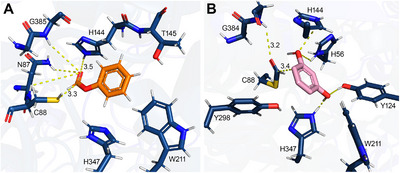
(A) Crystal structure of *Cs*ATase *PhlC* with donor substrate phenyl formate modeled into the active site by simulating the closed conformation of residue W211. (B) Crystal structure with acceptor substrate resorcinol modeled into the active site by extending the formyl moiety manually and simulating the closed conformation of residue W211. The shown distances are displayed in Å.

**SCHEME 4:**
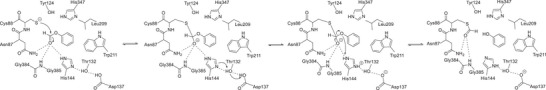
Plausible mechanism for the formyltransfer from phenyl formate to the active Cys88 of *Cs*ATase.

The formylation of resorcinol most likely consists of two half‐reactions: (i) the formylation of the enzyme by a formyl donor (first half‐reaction) and (ii) the transfer of the formyl group to the acceptor molecule, such as resorcinol (second half‐reaction). Separate experiments indicated that the formyl transfer occurs directly to the carbon of the resorcinol and not via O‐formylation followed by spontaneous rearrangement (see Supporting information). Docking of phenyl formate **2a** placed the donor in a conformation, where the formyl group is oriented toward the region defined by His144, Phe148, and Gly384 enabling a nucleophilic attack by the thiol group of Cys88 residue on the carbonyl carbon of phenyl formate (Figure 5A, Scheme 4), leading to the formation of a covalent enzyme–substrate intermediate. Stabilization of the negative charge on the oxygen atom in this alkoxide intermediate may be facilitated by an oxyanion hole formed by residues Gly385 and Asp87. To dock acceptor molecules to the active site, the formyl group was manually inserted at the C88 residue. Substrates **1a** (Figure 5B), **1h** and **1i** (Figure S39) were modeled into the active site using AutoDock Vina and Schrödinger Glide. The docking results obtained with AutoDock Vina corresponded well with the previously published crystal structure containing phloroglucinol (Figure S36). The phenolic hydroxyl group at *C*‐1 of **1a** points to the region defined by His56 and His144 (Figure 5B), and the hydroxyl group at *C*‐3 to His347 and Tyr124. The observed orientation of the hydroxyl groups is consistent with the mechanism in which deprotonation at *C*‐1 and *C*‐3 facilitates activation of the aromatic ring for electrophilic attack, followed by a C─C bond formation between the *C*‐6 carbon of the substrate and the formyl group covalently bonded to Cys88. Mutagenesis of these key residues (C88, H144, Y124, H347) in the active site resulted in complete loss of activity (Table S10), confirming their essential contribution to the catalytic mechanism. The docking results also indicated that the substrate binding site can accommodate bulkier substrates such as **1i** (Figure S39A) and **1h**; for the latter, its alkyl chain points toward to outside of the active site (Figure S39B,C).

## 3 Conclusion

Formylation of aromatic compounds has not been described to be catalyzed by an enzyme before. By screening a library of multimeric three‐component acyltransferases, the acyltransferase originating from *C. sphagni* (*Cs*ATase) was identified allowing to formylate resorcinol **1a** and derivatives as a promiscuous unprecedent activity with outstanding regioselectivity. Conversions up to 99% were reached (e.g., at 10 mM substrate concentration), and the products were isolated with up to 74% yield. Investigation of the substrate pattern indicated flexibility, especially for substitutions at positions 4 and 5. For resorcinol substrates, regioselective mono‐formylation was observed. In the case of 1,3,5‐trihydroxybenzene (phloroglucinol), the enzyme exhibited di‐formylation activity, giving an essential precursor for various bioactive compounds (e.g., robustadials, euglobals [91], sideroxylonals) at 99% conversion.

Structure elucidation by X‐ray crystallography of the wild‐type enzyme and the cysteine variant followed by docking provided insight into substrate binding and processing. Mutational analysis demonstrated that the cysteine residue at position 88 (C88) in the active site of *PhlC* is essential for catalysis. The same is true for H144, Y124, H347.

This study opens new avenues to develop unprecedented C─C bond forming reactions and paves the way for further studies to exploit enzymes for the C‐formylation of aromatic compounds.

## Conflicts of Interest

The authors declare no conflicts of interest.

## Supporting information




**Supporting File 1**: The authors have cited additional references within the Supporting Information [1–15].

**Supporting File 2**: anie71292‐sup‐0002‐Data.zip.

## Data Availability

The data that support the findings of this study are available in the supplementary material of this article.
