# Supplementary material for: Biocatalytic Regioselective C‐Formylation of Resorcinol Derivatives
Source: Angew Chem Int Ed Engl. 2026 Jan 29;65(10):e19387. doi: 10.1002/anie.202519387 (PMC12955522; doi:10.1002/anie.202519387)
Supplement: Supplementary file 1 — Supporting File 1: The authors have cited additional references within the Supporting Information [1–15]. [file ANIE-65-e19387-s002.pdf]

Biocatalytic Regioselective *C*-Formylation of Resorcinol Derivatives

Lilla Gal,[a] Suresh Rohan,[b] Anna Żądło-Dobrowolska,[a] Bianca Hilweg,[a] Judith Müller,[a] Kai Tittmann,*[b] Wolfgang Kroutil*[a,c]

[a] Institute of Chemistry, University of Graz, Heinrichstrasse 28, 8010 Graz, Austria

[b] Department of Molecular Enzymology, Georg August University Göttingen, Julia-Lermontowa-Weg 3, 37077 Göttingen, Germany

[c] Field of Excellence BioHealth, BioTech Med Graz, University of Graz, Austria

Supporting Information

Table of contents

1. Materials and methods 3

1.1. Chemicals and reagents 3

1.2. Strains 3

1.3. DNA- and protein sequences 3

1.4. Plasmid construction and design of *Cs*ATase 12

1.5. Mutagenesis of *Cs*ATase 12

1.6. Shake flask cultivation and expression of the recombinant acyltransferases (ATases) 14

1.7. Purification of *Cs*ATase and its variant 16

2. Analytical methods 18

2.1. Activity assay 18

3. Reaction optimization and substrate scope: Procedure and structural data 20

4. Synthesis of 1,3-phenylene diformate and its hydrolysis in aqueous reaction conditions 26

5. Spectroscopic data 27

6. X-ray crystallization and structure determination 34

7. Molecular docking 36

7.1. Protein structures and ligands 36

8. Additional figures 40

9. NMR spectra 41

10. References 52

1. Materials and methods

1.1. Chemicals and reagents

Chemicals and solvents were obtained from BLDpharm, Carl Roth, Deutero GmbH, Enamine, Honeywell, Sigma-Aldrich, Thermo Fisher Scientific Inc., TCl, VWR. Chemicals and used without further purification.

The SDS-PAGE loading buffer, SDS-PAGE gel and SDS-PAGE ladder were supplied by BioRad, GenScript and Thermo Fisher Scientific. SDS-PAGE was performed on a precast gel (SurePAGE™, Bis-Tris, 12%) using the PageRuler Unstained Protein Ladder (10−200 kDa) as a marker.

1.2. Strains

Escherichia coli DH5α was used for cloning. *E. coli* BL21(DE3) was used for the expression of ATases.

1.3. DNA- and protein sequences

Table S1. Plasmids used in this study.

Plasmids	Origin	Description/Comments (GeneBank ID)
*Pp*ATaseCH (pEG332)	Schmidt *et al.*, 2017[1]	Codon-optimized gene fragments *PhlA*, *PhlC* and *PhlB* based on *PhlACB* from *Pseudomonas protegens* (DSM19095)
*Cs*ATase (pEG821)	this study	Gene fragments *PhlA*, *PhlC* and *PhlB* from *Chromobacterium spaghni* (MKCS01000001.1)
pEG821-C88S	this study	Single mutation C88S introduced to pEG821
pEG821-Y124F	this study	Single mutation Y124F introduced to pEG821
pEG821-H144V	this study	Single mutation H144V introduced to pEG821
pEG821-H347V	this study	Single mutation H347V introduced to pEG821
*Pt*ATase (pEG846)	this study	Gene fragments *PhlA*, *PhlC* and *PhlB* from *Pseudomonas thivervalensis* (LRSO01000023.1)
*Pk*ATase (pEG845)	this study	Gene fragments *PhlA*, *PhlC* and *PhlB* from *Pseudomonas kilonensis* (GCA001269885.1)
*Ppi*ATase	this study	Gene fragments *PhlA*, *PhlC* and *PhlB* from *Pseudomonas piscis* (GCA009380155.1)
*Bt*ATase	this study	Gene fragments *PhlA*, *PhlC* and *PhlB* from *Brenneria tiliae* (GCA023347575.1)
*Va*ATase	this study	Gene fragments *PhlA*, *PhlC* and *PhlB* from *Vibrio aerogenes* (GCA024346755.1)
*Tp*ATase	this study	Gene fragments *PhlA*, *PhlC* and *PhlB* from *Thermofilum pendens* (GCA000015225.1)

Table S2. Primers used in this study.

Primer	Sequence (5’- 3’)
C88S_forward	ctaccagtagtagcgttagcttccagat
C88S_reverse	aattactgctggtaatgaaggtcggtg
Y124F_forward	tcacttcaattttgcagaatatattg
Y124F_reverse	tcggtcattttttcgaag
H144V_forward	gggtattagcgtgaccgatgccttcgc
H144V_ reverse	aggaagtaatcatattcacattc
H347V_forward	tggtcgcggtgtggcaagcggctg
H347V_ reverse	atattaccaccatcggtattc

Table S3. Sequence identities compared to the sequence of *Pp*ATaseCH.

Enzyme	Seq. Id. [%]			Average seq. id. [%]	Seq. Coverage [%]
	phlA	*phlB*	*phlC*		*phlC*
*Pt*ATase	84	88	94	89	100
*Pk*ATase	85	94	87	89	100
*Ppi*ATase	96	95	98	96	92
*Cs*ATase	76	75	91	81	100
*Bt*ATase	59	63	73	65	100
*Va*ATase	54	64	63	60	100
*Tp*ATase	35	33	30	33	100

In the gene sequence below, lower case letters are part of the vector backbone (IBA3plus), while the insert is shown in capital letters. Motifs were highlighted as follows: start/stop codon, EcoRI restriction site (GAATTC), BamHI restriction site (GGATCC), linkers, suffixes, prefixes between the subunit.

***Pp*ATaseCH** (codon-harmonized *phlACB* gene sequence, *P. protegens* DSM 19095, Genbank accession no.: KY173355)

PhlA
MNVKKIGIVSYGAGIPVCRLKVQEVINVWKNTDLKLVEENLGVTERAVLQPDEDVITLGVLAAQRALDKVPGHQIE
ALYLGTCTNPYDSRASASIILEMLGSGYDAYCADVQFAGKSGTSALQICQALVASGMTGSALAIGADTINRNTAPGD
LTESYAGAGAAALLIGSQDVIAEFDASFSCAADVADNIRPQGDRYIRSGMGLGSDKNSIGLEDQTRRAAEGLMAKL
HTSPADYDYVVFQQNLVSTPYSLAKHLGFNPKQVEPGIYAGNVGDAGSASPLLGLINVLDQARPGQKILLVSYGFG
AGSDAIALTVTDAIEQYQKHNKPLRELLESKIYVDYGTSIKYEFKYLRADYALTAYL
PhlC
MCARRVAIVSAAYTPKPGSSRVRQTFKEMIVESAYKALKDAKMHPREIQAVAYGYHGEGISEYGGLGPTISDALGIS
PAPTFMSTANCTSSSVSFQMGHQMVASGEYDIVLCGGFEKMTDHFNYAEYIGSSTECEYDYFLGISHTDAFALATAE
YFQKFGYAGREADVLATFGRQMRIYAQNTPTATRYGQPIPSLEVLKNSEACGSMLAWGEASGCAILVAEHLAHKY
TDKPVFVRGCAYTGVSHYFGTRFHNPTLHHPGLPKDVGMAVSANSIACAEIAYKKAGITAKDIDVAQVYDLLGAGL
IQMESMGICGKGQAGDFVLEGGIALDGQLPLNTDGGNIGRGHASGCDGILHITELFRQLRGESDNQVKGARIGVSQN
LGGYAAHNSVIVLSND
PhlB
MSMYPEQIHRMTTASMLREWREHGGKYRLEGSQCEECNEIFFPRRTVCGACNSLSVKPYRCARSGKIEVMAPAENP
ILAAMGYGETVPRIMAMVRLDDGLVIASEIVDVCDQQQLKVGAPVRMVIRKHVRESNLAWQYAYKFVLDI

5’gagaccgcggtcccGAATTCaaggagatatacatatgATGAATGTGAAGAAAATAGGTATCGTTAGCTACGGCGCCGGTATCC
CGGTATGTCGCCTGAAAGTTCAGGAAGTGATTAATGTTTGGAAGAATACCGATCTCAAACTGGTGGAGGAAAA
TCTCGGCGTTACGGAAAGGGCGGTGCTGCAACCGGACGAAGACGTTATTACCCTCGGGGTGCTGGCCGCCCAA
CGGGCGCTGGACAAGGTTCCAGGTCACCAGATTGAAGCGCTGTATCTGGGCACCTGCACCAACCCATACGATT
CCCGGGCGTCGGCTTCGATTATCCTGGAAATGCTCGGCAGCGGCTATGATGCGTACTGCGCGGATGTGCAGTTT
GCGGGCAAATCGGGAACTTCAGCGCTGCAGATTTGCCAGGCCCTGGTGGCTTCGGGCATGACGGGCAGCGCGC
TGGCGATTGGCGCGGATACCATTAATCGCAATACCGCGCCGGGCGATCTGACCGAATCTTACGCGGGGGCGGG
AGCGGCTGCCCTGCTGATTGGCAGCCAGGACGTTATTGCGGAATTTGATGCGAGCTTTTCTTGCGCGGCCGACG
TTGCGGATAATATTCGCCCACAGGGCGACCGCTATATCCGTTCGGGCATGGGCCTGGGCTCGGATAAAAATAG
CATTGGCCTGGAAGATCAGACCCGCCGTGCGGCCGAAGGCCTGATGGCGAAACTGCATACCAGCCCAGCGGAT
TACGATTACGTGGTTTTTCAGCAAAATCTGGTGTCGACGCCATATTCTCTGGCGAAACATCTGGGCTTTAATCC
AAAACAGGTGGAACCGGGCATTTACGCGGGCAATGTAGGCGACGCGGGATCGGCGAGCCCGCTGCTCGGCCT
GATTAATGTACTGGATCAGGCACGCCCGGGGCAGAAGATCCTTTTGGTGTCTTATGGTTTTGGCGCGGGCAGCG
ATGCGATTGCGCTGACCGTTACCGATGCGATTGAGCAGTATCAGAAACATAATAAACCTCTGCGCGAACTGCT
GGAATCTAAAATTTATGTTGATTATGGCACGTCTATTAAATATGAGTTTAAATATCTGCGGGCTGATTATGCGC
TGACCGCGTACCTCTAAggtaccaaggagatatacatatgATGTGCGCACGTCGCGTAGCAATTGTATCGGCGGCCTACACG
CCGAAGCCAGGAAGTTCACGAGTACGGCAGACGTTTAAAGAAATGATTGTTGAGTCTGCGTATAAAGCACTCA
AAGATGCGAAAATGCATCCACGGGAAATTCAGGCGGTGGCGTACGGTTACCATGGTGAAGGCATCTCGGAATA
CGGCGGTCTGGGCCCGACCATCTCTGATGCGCTGGGCATTAGCCCGGCCCCGACCTTTATGAGCACCGCGAATT
GCACCAGCAGCTCGGTGTCGTTTCAGATGGGCCATCAGATGGTGGCCTCGGGGGAGTATGATATTGTTCTGTGC
GGCGGTTTTGAGAAAATGACCGACCATTTTAATTATGCGGAATATATTGGCTCGAGCACTGAATGTGAATATG
ACTACTTTCTCGGCATCTCTCATACCGACGCGTTTGCGCTGGCGACCGCGGAGTATTTTCAGAAATTTGGCTAC
GCGGGTCGCGAGGCGGATGTACTGGCGACCTTTGGCCGGCAGATGCGCATTTATGCACAGAATACCCCAACCG
CGACCCGTTACGGCCAGCCGATCCCATCGCTGGAAGTGTTGAAAAATAGCGAAGCGTGCGGCTCGATGCTGGC
GTGGGGCGAAGCGAGTGGCTGCGCCATTCTGGTGGCGGAACATCTGGCGCATAAATATACCGACAAGCCGGTG
TTTGTACGCGGTTGCGCGTACACCGGGGTTTCTCATTACTTTGGTACGCGCTTCCATAATCCGACCCTGCACCAT
CCGGGCCTGCCAAAAGACGTGGGCATGGCCGTCTCGGCGAATTCTATTGCGTGTGCCGAGATTGCGTATAAAA
AGGCGGGGATTACCGCGAAAGATATTGATGTGGCGCAGGTTTATGATCTGCTCGGCGCAGGGCTGATTCAGAT

GGAATCTATGGGCATTTGTGGCAAAGGCCAGGCGGGCGATTTTGTGCTCGAAGGCGGTATCGCGCTGGACGGC
CAGCTGCCGCTCAACACCGATGGCGGTAATATTGGCCGCGGCCACGCGTCTGGCTGCGATGGCATTCTGCATAT
TACCGAGCTGTTTCGGCAGCTGAGAGGTGAATCGGATAATCAGGTTAAAGGCGCGCGCATTGGCGTGTCGCAG
AATCTTGGCGGTTATGCCGCGCATAATTCCGTGATCGTTCTCTCGAATGATTGAatttaaatcacatgctcggctcagtcgcctgcgg
atgcatggtctggatgtgccgaggtctgcgtcggtgcagactcagtcgacgagatctaaggagatatacatatgATGTCTATGTATCCTGAACAGATTCATA
GAATGACCACCGCGAGCATGCTTCGCGAATGGCGCGAGCACGGCGGTAAATATCGCCTCGAAGGCAGCCAGT
GCGAAGAATGCAATGAAATTTTTTTCCCACGGCGCACCGTTTGCGGCGCTTGCAATTCTCTGAGTGTGAAACCG
TACCGCTGCGCCCGCAGTGGCAAAATTGAGGTTATGGCGCCGGCGGAGAATCCGATTCTGGCGGCCATGGGCT
ACGGCGAAACCGTGCCGCGCATTATGGCGATGGTGCGCCTGGATGATGGCCTGGTGATTGCTTCGGAAATTGT
TGACGTGTGCGACCAGCAACAGCTGAAGGTTGGTGCGCCGGTGCGCATGGTGATTCGCAAACACGTGCGCGAA
AGCAATCTGGCGTGGCAATATGCTTACAAATTTGTACTCGACATATGAggcgcgcccacatgctcggctcagtcGGATCCctcga
ggtcgacctg-3’

***Cs*ATase** (*phlACB* gene sequence, *Chromobacterium sphagni*)

PhlA
MKKIGIVSYGSSIPTCRLKINDVIDVWKNTDLDLVKNHLGVCERAVLQPDEDVITLGVQAAQRALEHAGSPTLDAL
HLGTCTNPYDSRSSAAIILEMLGQGYDMYCADVQFSGKSGTSALQISQALVASGMAGHALAIAADAINRHTAPGDL
TESYAGAGAAAMLVGSENLIAEIDGTFSCAADIADNIRPQGERYIRSGMGLGSDKNSIGLEDQTRRAAEGLMGKLKT
SASDFDYVVFQQNVVSTPRSLGKLLGFTAEQLEPALFADTIGDTGAASPLLGLIQVLDQAKPGDRILLVSYGFGAGS
DAIALTVTDNIAAHQQRATTLKTQLGQKQYVDYGTAIKYEFKYLRPDYALTAYL
PhlC
MSARRVAIVSAAYTPKPGSSRVRQTFKEMIVESAYKAIRDAKMHPREIQGVAYGYHGEGISEYGGLGPTISDALGISP
APTFITSSNCTSSSVSFQMGHQMVASGEYDIVLCGGFEKMTDHFNYAEYIGSSTECEYDYFLGISHTDAFALATSEYF
HKYGYAGREADVLAHFGRQMRIYAQNTPSATRYGQAIPSLEALKASEAHGSMLAWGEASGCAILVAEHLAHKYTD
KPVFIKGCAYTGVSHYFGTRYHNPTLQYPGLPQDVGMALSANSIACAEMAYKKAGITAKDIDVAQVYDLLGAGLIQ
MESMGICQPGQAGHFVMEGGIALDGQVPLNTDGGNIGRGHASGCDGILHITELFRQLRGEADNQVKNARIAVSQNL
GGYAAHNSVIVLTNE
PhlB
MSLYPEHIHRMTTASMLREWREHGGKYRLEGSRCQECEAIFFPRRSVCGACNSLKIEPYGCKRHGTIVALSRAENPI
LAGMGYGEAVPRHMVMLRLDDGIGIASEVVDILDPAKLKIGARVKMVIRKHVRESNLAWQYAYKFVLE

DNA sequence of *Cs*ATase – first approach

5’gagaccgcggtcccGAATTCgagctcggtaccATGAAGAAGATCGGCATTGTGAGCTATGGTAGTAGTATTCCGACCTGCC
GCCTGAAAATTAATGATGTGATTGATGTGTGGAAGAATACCGATCTGGATCTGGTTAAAAATCATCTGGGTGT
GTGTGAACGTGCAGTTCTGCAGCCGGATGAAGATGTGATTACCCTGGGTGTGCAGGCAGCACAGCGCGCACTG
GAACATGCCGGTAGCCCGACCCTGGATGCACTGCATCTGGGCACCTGTACCAATCCGTATGATAGCCGTAGCA
GCGCAGCCATTATTCTGGAAATGCTGGGTCAGGGTTATGATATGTATTGTGCAGATGTGCAGTTCAGCGGCAA
AAGCGGCACCAGTGCCCTGCAGATTAGCCAGGCACTGGTTGCCAGTGGCATGGCAGGTCATGCCCTGGCCATT
GCCGCCGATGCAATTAATCGCCATACCGCACCGGGCGATCTGACCGAAAGCTATGCAGGTGCCGGCGCCGCAG
CAATGCTGGTGGGTAGTGAAAATCTGATTGCCGAAATTGATGGCACCTTCAGCTGTGCCGCCGATATTGCAGAT
AATATTCGCCCGCAGGGTGAACGTTATATTCGCAGCGGTATGGGTCTGGGTAGCGATAAAAATAGTATTGGCC
TGGAAGATCAGACCCGTCGTGCCGCCGAAGGTCTGATGGGCAAACTGAAAACCAGCGCAAGCGACTTCGATTA
TGTTGTGTTCCAGCAGAATGTTGTTAGTACCCCGCGCAGTCTGGGCAAACTGCTGGGCTTCACCGCCGAACAGC
TGGAACCGGCACTGTTCGCCGATACCATTGGTGATACCGGTGCCGCAAGCCCGCTGCTGGGTCTGATTCAGGTG
CTGGATCAGGCAAAACCGGGCGATCGTATTCTGCTGGTGAGTTATGGCTTCGGTGCAGGTAGCGATGCCATTG
CCCTGACCGTGACCGATAATATTGCAGCCCATCAGCAGCGTGCAACCACCCTGAAAACACAACTGGGTCAGAA
ACAGTATGTGGATTATGGTACCGCAATTAAATATGAGTTCAAATATCTGCGTCCGGATTATGCCCTGACCGCCT
ATCTGTAAttacgcctgtctacgcaaggagcATGAGTGCACGTCGTGTTGCAATTGTGAGTGCCGCATATACCCCGAAACCG
GGTAGCAGCCGTGTGCGCCAGACCTTCAAAGAAATGATTGTGGAAAGTGCATATAAGGCAATTCGCGATGCAA
AAATGCATCCGCGCGAAATTCAGGGCGTGGCATATGGTTATCATGGTGAAGGTATTAGCGAATATGGCGGTCT
GGGCCCGACCATTAGCGATGCACTGGGCATTAGCCCGGCACCGACCTTCATTACCAGCAGTAATTGTACCAGT
AGTAGCGTTAGCTTCCAGATGGGCCATCAGATGGTGGCCAGCGGTGAATATGATATTGTTCTGTGCGGCGGCTT
CGAAAAAATGACCGATCACTTCAATTATGCAGAATATATTGGTAGCAGTACCGAATGTGAATATGATTACTTCC
TGGGTATTAGCCATACCGATGCCTTCGCACTGGCCACCAGTGAATACTTCCATAAATATGGCTATGCAGGTCGC
GAAGCCGATGTGCTGGCACACTTCGGTCGTCAGATGCGTATCTATGCACAGAATACCCCGAGCGCCACCCGTT
ATGGTCAGGCAATTCCGAGCCTGGAAGCCCTGAAAGCCAGTGAAGCCCATGGTAGCATGCTGGCATGGGGCGA
AGCAAGCGGCTGCGCAATTCTGGTGGCCGAACATCTGGCACATAAATATACCGATAAACCGGTGTTCATTAAA
GGTTGCGCCTATACCGGTGTTAGCCATTACTTCGGTACCCGTTATCATAATCCGACCCTGCAGTATCCGGGCCT
GCCGCAGGATGTTGGCATGGCCCTGAGCGCCAATAGCATTGCATGCGCCGAAATGGCCTATAAAAAAGCCGGC
ATTACCGCAAAAGATATTGATGTTGCACAGGTGTATGATCTGCTGGGTGCAGGTCTGATTCAGATGGAAAGTA
TGGGCATCTGTCAGCCGGGTCAGGCAGGTCACTTCGTGATGGAAGGCGGCATTGCCCTGGATGGTCAGGTGCC
GCTGAATACCGATGGTGGTAATATTGGTCGCGGTCATGCAAGCGGCTGTGATGGCATTCTGCATATTACCGAAC
TGTTCCGCCAGCTGCGCGGTGAAGCCGATAATCAGGTGAAAAATGCCCGCATTGCCGTTAGTCAGAATCTGGG
TGGCTATGCCGCCCATAATAGTGTTATTGTTCTGACCAATGAATAAggagcccggaccATGAGCCTGTATCCGGAACA
TATTCATCGTATGACCACCGCAAGCATGCTGCGCGAATGGCGTGAACATGGCGGCAAATATCGCCTGGAAGGC

AGTCGCTGTCAGGAATGCGAAGCCATCTTCTTCCCGCGTCGCAGTGTGTGTGGCGCATGTAATAGTCTGAAAAT
TGAACCGTATGGCTGCAAACGTCATGGCACCATTGTTGCACTGAGCCGTGCCGAAAATCCGATTCTGGCAGGT
ATGGGTTATGGCGAAGCAGTTCCGCGTCATATGGTGATGCTGCGTCTGGATGATGGCATTGGCATTGCAAGTG
AAGTTGTTGATATTCTGGACCCTGCCAAACTGAAAATTGGTGCACGTGTTAAAATGGTGATTCGCAAACATGTT
CGTGAAAGCAATCTGGCCTGGCAGTATGCATATAAATTCGTGCTGGAATAAGGATCCctcgaggtcgacctg-3’

Improved DNA sequence of *Cs*ATase

5’gagaccgcggtcccGAATTCaaggagatatacatATGAAGAAGATCGGCATTGTGAGCTATGGTAGTAGTATTCCGACCTGC
CGCCTGAAAATTAATGATGTGATTGATGTGTGGAAGAATACCGATCTGGATCTGGTTAAAAATCATCTGGGTGT
GTGTGAACGTGCAGTTCTGCAGCCGGATGAAGATGTGATTACCCTGGGTGTGCAGGCAGCACAGCGCGCACTG
GAACATGCCGGTAGCCCGACCCTGGATGCACTGCATCTGGGCACCTGTACCAATCCGTATGATAGCCGTAGCA
GCGCAGCCATTATTCTGGAAATGCTGGGTCAGGGTTATGATATGTATTGTGCAGATGTGCAGTTCAGCGGCAA
AAGCGGCACCAGTGCCCTGCAGATTAGCCAGGCACTGGTTGCCAGTGGCATGGCAGGTCATGCCCTGGCCATT
GCCGCCGATGCAATTAATCGCCATACCGCACCGGGCGATCTGACCGAAAGCTATGCAGGTGCCGGCGCCGCAG
CAATGCTGGTGGGTAGTGAAAATCTGATTGCCGAAATTGATGGCACCTTCAGCTGTGCCGCCGATATTGCAGAT
AATATTCGCCCGCAGGGTGAACGTTATATTCGCAGCGGTATGGGTCTGGGTAGCGATAAAAATAGTATTGGCC
TGGAAGATCAGACCCGTCGTGCCGCCGAAGGTCTGATGGGCAAACTGAAAACCAGCGCAAGCGACTTCGATTA
TGTTGTGTTCCAGCAGAATGTTGTTAGTACCCCGCGCAGTCTGGGCAAACTGCTGGGCTTCACCGCCGAACAGC
TGGAACCGGCACTGTTCGCCGATACCATTGGTGATACCGGTGCCGCAAGCCCGCTGCTGGGTCTGATTCAGGTG
CTGGATCAGGCAAAACCGGGCGATCGTATTCTGCTGGTGAGTTATGGCTTCGGTGCAGGTAGCGATGCCATTG
CCCTGACCGTGACCGATAATATTGCAGCCCATCAGCAGCGTGCAACCACCCTGAAAACACAACTGGGTCAGAA
ACAGTATGTGGATTATGGTACCGCAATTAAATATGAGTTCAAATATCTGCGTCCGGATTATGCCCTGACCGCCT
ATCTGTAAggtaccaaggagatatacatATGAGTGCACGTCGTGTTGCAATTGTGAGTGCCGCATATACCCCGAAACCGGG
TAGCAGCCGTGTGCGCCAGACCTTCAAAGAAATGATTGTGGAAAGTGCATATAAGGCAATTCGCGATGCAAAA
ATGCATCCGCGCGAAATTCAGGGCGTGGCATATGGTTATCATGGTGAAGGTATTAGCGAATATGGCGGTCTGG
GCCCGACCATTAGCGATGCACTGGGCATTAGCCCGGCACCGACCTTCATTACCAGCAGTAATTGTACCAGTAGT
AGCGTTAGCTTCCAGATGGGCCATCAGATGGTGGCCAGCGGTGAATATGATATTGTTCTGTGCGGCGGCTTCGA
AAAAATGACCGATCACTTCAATTATGCAGAATATATTGGTAGCAGTACCGAATGTGAATATGATTACTTCCTGG
GTATTAGCCATACCGATGCCTTCGCACTGGCCACCAGTGAATACTTCCATAAATATGGCTATGCAGGTCGCGAA
GCCGATGTGCTGGCACACTTCGGTCGTCAGATGCGTATCTATGCACAGAATACCCCGAGCGCCACCCGTTATGG
TCAGGCAATTCCGAGCCTGGAAGCCCTGAAAGCCAGTGAAGCCCATGGTAGCATGCTGGCATGGGGCGAAGC
AAGCGGCTGCGCAATTCTGGTGGCCGAACATCTGGCACATAAATATACCGATAAACCGGTGTTCATTAAAGGT
TGCGCCTATACCGGTGTTAGCCATTACTTCGGTACCCGTTATCATAATCCGACCCTGCAGTATCCGGGCCTGCC
GCAGGATGTTGGCATGGCCCTGAGCGCCAATAGCATTGCATGCGCCGAAATGGCCTATAAAAAAGCCGGCATT
ACCGCAAAAGATATTGATGTTGCACAGGTGTATGATCTGCTGGGTGCAGGTCTGATTCAGATGGAAAGTATGG
GCATCTGTCAGCCGGGTCAGGCAGGTCACTTCGTGATGGAAGGCGGCATTGCCCTGGATGGTCAGGTGCCGCT
GAATACCGATGGTGGTAATATTGGTCGCGGTCATGCAAGCGGCTGTGATGGCATTCTGCATATTACCGAACTGT
TCCGCCAGCTGCGCGGTGAAGCCGATAATCAGGTGAAAAATGCCCGCATTGCCGTTAGTCAGAATCTGGGTGG
CTATGCCGCCCATAATAGTGTTATTGTTCTGACCAATGAATAAatttaaatcacatgctcggctcagtcgcctgcggatgcatggtctggatgt
gccgaggtctgcgtcggtgcagactcagtcgacgagatctaaggagatatacatATGAGCCTGTATCCGGAACATATTCATCGTATGACCACCG
CAAGCATGCTGCGCGAATGGCGTGAACATGGCGGCAAATATCGCCTGGAAGGCAGTCGCTGTCAGGAATGCG
AAGCCATCTTCTTCCCGCGTCGCAGTGTGTGTGGCGCATGTAATAGTCTGAAAATTGAACCGTATGGCTGCAAA
CGTCATGGCACCATTGTTGCACTGAGCCGTGCCGAAAATCCGATTCTGGCAGGTATGGGTTATGGCGAAGCAG
TTCCGCGTCATATGGTGATGCTGCGTCTGGATGATGGCATTGGCATTGCAAGTGAAGTTGTTGATATTCTGGAC
CCTGCCAAACTGAAAATTGGTGCACGTGTTAAAATGGTGATTCGCAAACATGTTCGTGAAAGCAATCTGGCCT
GGCAGTATGCATATAAATTCGTGCTGGAATGAggcgcgcccacatgctcggctcagtcGGATCCctcgaggtcgacctg-3’

***Pt*ATase** (*phlACB* gene sequence, *Pseudomonas thivervalensis*)

phlA
MNKVGIVSYGAGIPVCRLKVDDVIQVWKNTDLSLVKGQLGVIERAVLQPDEDVITLSVLAAQRALDKAPPCSLEAL
YLGTCTNPYDSRASAAIILEMLGCGYDAFCADVQFAGKSGTSALQIAYALVASGMVGNALAVGADTINRNTAPGD
LTESYAGAGAAALLLGTENVIAHFDASFSCAADVADNIRPQGDRYIRSGMGLGSDKNSIGLEDQTRRAASGLMAKI
HAQAGDFDYVVFQQNLVSTPYSLGKHLGFTSAQIEPGIYAQSVGDAGAASPLLGLVNVLDQARPGERILMVSYGFG
AGSDAIALTVTDAIEAYQKTNVPLRTLLEDKYYVDYGTSIKYEFKYLRPDYALTAYL
phlC
MCARRVAIVSAAYTPKPGSSRVRQTFKEMIVESAYQALNAIKMHPRELQAVAYGYHGEGISEYGGLGPTISDALGIS
PAPTFMSTANCTSSSVSFQMAHQMVASGEYDIVLCGGFEKMTDHFNYAEYIGSSTECEYDYFLGISHTDAFALATAE
YFEKFGYAGREADVLATFGRQMRIYAHNTPTATRYGVPIPSLDTLKSSEACGSMLAWGEASGCAILVAEHLVHRYT
TQPVFIRGCAYTGVSHYFGTRYHNPTLQFPGLPKDVGMAVSANSMACAEIAYKKAGITAKDIDVAQVYDLLGAGLI
QMESMGVCGPGQAGDFVLEGGIALDGQLPLNTDGGNIGRGHASGCDGILHITELFRQLRGESNNQVKGARIGVSQN
LGGYAAHNSVIVLSND
phlB
MSLYPEQIHRMTTASMLREWREHGGKYRLEGSQCQDCEEIFFPRRTVCGACNSLNVEPYRCARTGTIQVSAHAENPI
LAAMGYGETVPRFMAMVRLDDGLVIASEIVDVIDSRQVVSGAPVRMVIRKHVRESNLAWQYAYKFVL

5’gagaccgcggtcccGAATTCgagctcggtaccATGAACAAGGTTGGTATTGTTAGCTATGGTGCCGGTATTCCGGTGTGTCG
CCTGAAAGTGGATGATGTTATTCAGGTGTGGAAAAATACCGATCTGAGCCTGGTTAAAGGCCAGCTGGGTGTG
ATTGAACGCGCAGTGCTGCAGCCGGATGAAGATGTTATTACCCTGAGCGTGCTGGCCGCCCAGCGCGCATTAG
ATAAAGCACCGCCGTGCAGCCTGGAAGCACTGTATCTGGGTACCTGCACCAATCCGTATGATAGTCGCGCAAG
CGCAGCAATTATTCTGGAAATGCTGGGCTGCGGTTATGATGCATTCTGTGCAGATGTTCAGTTCGCAGGTAAAA
GCGGTACCAGCGCCCTGCAGATTGCATATGCACTGGTGGCAAGTGGTATGGTTGGCAATGCACTGGCAGTTGG
CGCAGATACCATTAATCGCAATACCGCCCCGGGTGATCTGACCGAAAGTTATGCCGGTGCCGGTGCAGCCGCA
CTGCTGTTAGGCACCGAAAATGTTATTGCACACTTCGATGCAAGCTTCAGTTGTGCCGCCGATGTGGCAGATAA
TATTCGCCCGCAGGGCGATCGTTATATTCGTAGTGGTATGGGCCTGGGTAGTGATAAAAATAGCATTGGCCTGG
AAGATCAGACCCGTCGTGCAGCAAGCGGTCTGATGGCCAAAATTCATGCCCAGGCCGGTGACTTCGATTATGT
GGTGTTCCAGCAGAATCTGGTGAGCACCCCGTATAGCCTGGGTAAACATCTGGGCTTCACCAGTGCACAGATT
GAACCGGGCATCTATGCACAGAGCGTTGGCGATGCAGGCGCAGCAAGCCCGCTGCTGGGTCTGGTGAATGTGC
TGGATCAGGCCCGCCCGGGTGAACGCATTCTGATGGTGAGCTATGGCTTCGGTGCAGGTAGCGATGCAATTGC
ACTGACCGTTACCGATGCAATTGAAGCCTATCAGAAAACCAATGTGCCGCTGCGTACCCTGCTGGAAGATAAA
TATTATGTGGATTATGGTACCAGCATTAAATATGAGTTCAAATATCTGCGTCCGGATTATGCACTGACCGCATA
TCTGTAAttacgcctgtctacgcaaggagcATGTGTGCCCGCCGCGTTGCAATTGTTAGTGCCGCATATACCCCGAAACCGG
GCAGTAGCCGTGTGCGTCAGACCTTCAAAGAAATGATTGTGGAAAGTGCCTATCAGGCACTGAATGCAATTAA
AATGCATCCGCGTGAACTGCAGGCCGTGGCCTATGGTTATCATGGTGAAGGCATTAGCGAATATGGTGGTCTG
GGTCCGACCATTAGTGATGCCCTGGGCATTAGTCCGGCCCCGACCTTCATGAGTACCGCCAATTGTACCAGCAG
CAGCGTTAGCTTCCAGATGGCCCATCAGATGGTGGCAAGCGGTGAATATGATATTGTGCTGTGCGGTGGCTTCG
AAAAAATGACCGATCACTTCAATTATGCAGAATATATTGGTAGTAGCACCGAATGTGAATATGATTACTTCCTG
GGTATTAGCCATACCGATGCATTCGCACTGGCCACCGCCGAATACTTCGAAAAATTCGGTTATGCAGGTCGTGA
AGCAGATGTGCTGGCAACCTTCGGCCGCCAGATGCGCATCTATGCCCATAATACCCCGACCGCAACCCGTTAT
GGCGTTCCGATTCCGAGCCTGGATACCCTGAAAAGTAGTGAAGCATGCGGTAGCATGCTGGCATGGGGTGAAG
CCAGCGGTTGCGCAATTCTGGTTGCCGAACATCTGGTGCATCGTTATACCACCCAGCCGGTGTTCATTCGCGGC
TGTGCCTATACCGGCGTTAGTCATTACTTCGGCACCCGCTATCATAATCCGACCCTGCAGTTCCCGGGTCTGCC
GAAAGATGTTGGCATGGCAGTGAGTGCAAATAGTATGGCATGTGCAGAAATTGCATATAAAAAAGCAGGCATT
ACCGCCAAAGATATTGATGTGGCACAGGTGTATGATCTGCTGGGTGCCGGTCTGATTCAGATGGAAAGCATGG
GCGTGTGTGGTCCGGGCCAGGCCGGTGACTTCGTGCTGGAAGGCGGTATTGCCCTGGATGGTCAGCTGCCGCT
GAATACCGATGGTGGTAATATTGGCCGTGGTCATGCAAGCGGCTGTGATGGTATTCTGCATATTACCGAACTGT
TCCGCCAGCTGCGCGGCGAAAGTAATAATCAGGTTAAAGGTGCACGCATTGGTGTTAGCCAGAATCTGGGTGG
CTATGCCGCCCATAATAGCGTGATTGTGCTGAGCAATGATTAAggagcccggaccATGAGTCTGTATCCGGAACAGA
TTCATCGCATGACCACCGCAAGTATGCTGCGTGAATGGCGTGAACATGGCGGTAAATATCGCCTGGAAGGCAG
CCAGTGTCAGGATTGCGAAGAAATCTTCTTCCCGCGTCGTACCGTGTGTGGCGCATGTAATAGTCTGAATGTGG
AACCGTATCGCTGTGCACGCACCGGTACCATTCAGGTTAGCGCCCATGCAGAAAATCCGATTCTGGCCGCCAT
GGGTTATGGCGAAACCGTTCCGCGCTTCATGGCAATGGTGCGCCTGGATGATGGTCTGGTGATTGCAAGTGAA
ATTGTGGATGTTATTGATAGCCGCCAGGTTGTTAGCGGCGCCCCGGTGCGTATGGTTATTCGCAAACATGTGCG
TGAAAGTAATCTGGCCTGGCAGTATGCCTATAAATTCGTTCTGTAAGGATCCctcgaggtcgacctg-3’

***Pk*ATase** (*phlACB* gene sequence, *Pseudomonas kilonensis*)

phlA
MNKVGIVSYGAGIPVCRLKVDDVIQVWKNTDLSLVKGQLGILERAVLQPDEDVITLGVLAAQRALDKAPPCSLEAL
YLGTCTNPYDSRASAAIILEMLGCGYDAFCADVQFAGKSGTSALQIAYALVASGMVGNALAVGADTINRNTAPGD
LTESYAGAGAAALLLGTENVIAHFDASFSCAADVADNIRPQGDRYIRSGMGLGSDKNSIGLEDQTRRAASGLMAKI
HAQAADFDYVVFQQNLVSTPYSLGKHLGFTTAQIEPGIYAQNVGDAGAASPLLGLVNVLDQARPGERILVVSYGFG
AGSDAIALTVTDAIEAYQKTNVPLRTLLEDKYYVDYGTSIKYEFKYLRPDYALTAYL
phlC
MSARRVAIVSAAYTPKPGSSRVRQTFKEMIVESAYQALNAIKMHPRELQAVAYGYHGEGISEYGGLGPTISDALGIS
PAPTFMSTANCTSSSVSFQMAHQMVASGEYDIVLCGGFEKMTDHINYAEYIGSSTECEYDYFLGISHTDAFALATAE
YFEKFGYAGREADVLATFGRQMRIYAHNTPMATRYGVPIPSLEALKSSEACGSMLAWGEASGCAILVAEHLAHRY
TTQPVFIRGCAYTGVSHYFGTRYHNPTLQFPGLPKDVGMAVSANSIACAEIAYKKAGITAKDIDVAQVYDLLGAGLI
QMESMGVCGPGQAGDFVLEGGIALDGQLPLNTDGGNIGRGHASGCDGILHITELFRQLRGESNNQVKGARIGVSQN
LGGYAAHNSVIVLSND
phlB
MSLYPEQIHRMTTASMLREWREHGGKYRLEGSRCQDCDEIFFPRRTVCGACNSLNVEPYRCARTGTIQVSARAENPI
LAAMGYGETVPRFMAMVRLDDGLVIASEIVDVIDPRQVVSGAPVRMVIRKHVRESNLAWQYAYKFVL

5’gagaccgcggtcccGAATTCgagctcggtaccATGAACAAGGTTGGCATTGTTAGCTATGGCGCCGGTATTCCGGTGTGTCG
CCTGAAAGTGGATGATGTTATTCAGGTGTGGAAAAATACCGATCTGAGCCTGGTGAAAGGCCAGCTGGGCATT
CTGGAACGCGCCGTTCTGCAGCCGGATGAAGATGTTATTACCCTGGGTGTTCTGGCAGCCCAGCGCGCCCTGG
ATAAAGCCCCGCCTTGCAGCCTGGAAGCACTGTATCTGGGTACCTGCACCAATCCGTATGATAGTCGCGCCAG
CGCAGCCATTATTCTGGAAATGCTGGGTTGTGGTTATGATGCCTTCTGTGCCGATGTTCAGTTCGCCGGTAAAA
GTGGTACCAGCGCCCTGCAGATTGCCTATGCACTGGTTGCAAGTGGTATGGTTGGCAATGCACTGGCCGTGGG
CGCAGATACCATTAATCGTAATACCGCACCGGGTGATCTGACCGAAAGTTATGCAGGTGCAGGCGCCGCCGCC
CTGCTGTTAGGTACCGAAAATGTGATTGCCCACTTCGATGCAAGCTTCAGTTGTGCCGCAGATGTTGCCGATAA
TATTCGTCCGCAGGGCGATCGTTATATTCGTAGCGGCATGGGCCTGGGTAGTGATAAAAATAGCATTGGCCTG

GAAGATCAGACCCGTCGCGCCGCCAGCGGTCTGATGGCAAAAATTCATGCCCAGGCCGCAGACTTCGATTATG
TTGTGTTCCAGCAGAATCTGGTGAGTACCCCGTATAGTCTGGGCAAACATCTGGGCTTCACCACCGCACAGATT
GAACCGGGCATCTATGCCCAGAATGTGGGTGATGCAGGCGCAGCAAGTCCGCTGCTGGGCCTGGTGAATGTGC
TGGATCAGGCCCGCCCGGGTGAACGTATTCTGGTGGTGAGCTATGGCTTCGGTGCCGGTAGCGATGCAATTGC
CCTGACCGTGACCGATGCCATTGAAGCATATCAGAAAACCAATGTGCCGCTGCGCACCCTGCTGGAAGATAAA
TATTATGTTGATTACGGTACCAGTATCAAATATGAGTTCAAATATCTGCGTCCGGATTATGCACTGACCGCCTA
TCTGTAAttacgcctgtctacgcaaggagcATGCACATGAGCGCCCGTCGTGTGGCCATTGTTAGTGCCGCCTATACCCCGAA
ACCGGGTAGCAGTCGCGTGCGTCAGACCTTCAAAGAAATGATTGTGGAAAGCGCATATCAGGCCCTGAATGCC
ATTAAAATGCATCCGCGCGAACTGCAGGCCGTTGCATATGGTTATCATGGCGAAGGTATTAGCGAATATGGTG
GCCTGGGTCCGACCATTAGTGATGCACTGGGTATTAGCCCGGCCCCGACCTTCATGAGCACCGCCAATTGCACC
AGCAGCAGCGTGAGCTTCCAGATGGCCCATCAGATGGTTGCAAGCGGTGAATATGATATTGTGCTGTGTGGTG
GCTTCGAAAAAATGACCGATCATATTAATTACGCCGAATATATTGGCAGCAGTACCGAATGTGAATATGATTA
CTTCCTGGGCATTAGTCATACCGATGCATTCGCACTGGCCACCGCAGAATACTTCGAAAAATTCGGTTATGCCG
GCCGCGAAGCCGATGTGCTGGCCACATTCGGCCGTCAGATGCGTATCTATGCACATAATACCCCGATGGCCAC
CCGTTATGGTGTGCCGATTCCGAGTCTGGAAGCCCTGAAAAGCAGCGAAGCCTGCGGCAGCATGCTGGCCTGG
GGTGAAGCCAGTGGTTGCGCCATTCTGGTTGCCGAACATCTGGCACATCGCTATACCACCCAGCCGGTGTTCAT
TCGCGGCTGTGCATATACCGGCGTTAGCCATTACTTCGGTACCCGCTATCATAATCCGACCCTGCAGTTCCCGG
GCCTGCCGAAAGATGTTGGTATGGCAGTTAGTGCCAATAGCATTGCATGTGCCGAAATTGCATATAAAAAAGC
AGGCATTACCGCCAAAGATATTGATGTGGCACAGGTGTATGATCTGCTGGGCGCCGGTCTGATTCAGATGGAA
AGCATGGGCGTGTGCGGCCCGGGCCAGGCAGGTGACTTCGTGCTGGAAGGTGGTATTGCACTGGATGGCCAGC
TGCCGCTGAATACCGATGGTGGTAATATTGGTCGCGGTCATGCAAGCGGTTGTGATGGCATTCTGCATATTACC
GAACTGTTCCGTCAGCTGCGTGGCGAAAGCAATAATCAGGTGAAAGGCGCCCGTATTGGTGTGAGTCAGAATC
TGGGTGGTTATGCCGCACATAATAGTGTTATTGTTCTGAGTAATGACTAAggagcccggaccATGAGCCTGTATCCGG
AACAGATTCATCGCATGACCACCGCCAGTATGCTGCGTGAATGGCGTGAACATGGCGGCAAATATCGCCTGGA
AGGCAGTCGTTGTCAGGATTGTGATGAAATCTTCTTCCCGCGTCGTACCGTGTGTGGTGCCTGCAATAGTCTGA
ATGTGGAACCGTATCGTTGCGCCCGCACCGGCACCATTCAGGTTAGCGCCCGTGCCGAAAATCCGATTCTGGCC
GCCATGGGTTATGGTGAAACCGTGCCGCGCTTCATGGCAATGGTGCGTCTGGATGATGGCCTGGTTATTGCAAG
CGAAATTGTTGATGTGATTGATCCGCGCCAGGTGGTTAGTGGTGCACCGGTGCGTATGGTTATTCGCAAACATG
TTCGTGAAAGCAATCTGGCCTGGCAGTATGCCTATAAATTCGTTCTGTAAGGATCCctcgaggtcgacctg-3’

***Ppi*ATase** (*phlACB* gene sequence, *Pseudomonas piscis*)

phlA
MNVKKVGIVSYGAGIPVCRLKVQEVINVWKNTDLKLVEENLGVTERAVLQPDEDVITLGVLAAQRALDKVPGHEL
QALYLGTCTNPYDSRASASIILEMLGSGYDAYCADVQFAGKSGTSALQICQALVASGMTGSALAIGADTINRNTAPG
DLTESYAGAGAAALLVGTENVIAEFDGSFSCAADIADNIRPQGDRYIRSGMGLGSDKNSIGLEDQTRRAAEGLMAK
LHTSAADYDYVVFQQNLVSTPYSLGKHLGFNAKQIEPGIYAGSVGDTGAASPLLGLINVLDQARPGQKILMVSYGF
GAGSDAIALTVTDAIGQYQKRNKPLRQLLEDKTYVDYGTSIKYEFKYLRADYALTAYL
phlC
MIVESAYKAIKDAKMHPREIQGVAYGYHGEGISEYGGLGPTISDALGISPAPTFMSTANCTSSSVSFQMGHQMVASG
EYDIVLCGGFEKMTDHFNYAEYIGSSTECEYDYFLGISHTDAFALATAEYFQKFGYAGREADVLATFGRQMRIYAH
NTPTATRYGQAIPTLEALKNSEACGSMLAWGEASGCAILVAEHLAHKYTDKPVFVRGCAYTGVSHYFGTRFHNPTL
HHPGLPKDVGMAVSANSIACAEIAYKKAGITPKDIDVAQVYDLLGAGLIQMESMGVCGKGQAGDFVLEGGIALDG
QLPLNTDGGNIGRGHASGCDGILHITELFRQLRGEADNQVKGARIGVSQNLGGYAAHNSVIVLSND
phlB
MSMYPEQIHRMTTASMLREWREHGGKYRLEGSQCEECNEIFFPRRTVCGACNSMSVKPYRCARTGKIEVMAHADN
PILAAMGYGETVPRCMAMIRLDDGLVIASEIVDVCDAQQLKVGAPVRMVVRKHVRESNLAWQYAYKFVLD

5’gagaccgcggtcccGAATTCgagctcggtaccATGAACGTTAAGAAGGTGGGCATTGTTAGCTATGGTGCCGGCATTCCGG
TGTGCCGTCTGAAAGTTCAGGAAGTGATTAATGTGTGGAAAAATACCGATCTGAAACTGGTTGAAGAAAATCT
GGGCGTTACCGAACGCGCCGTTCTGCAGCCGGATGAAGATGTTATTACCCTGGGTGTTCTGGCAGCACAGCGT
GCACTGGATAAAGTGCCGGGTCATGAACTGCAGGCCCTGTATCTGGGCACCTGTACCAATCCGTATGATAGTC
GCGCCAGTGCAAGTATTATTCTGGAAATGCTGGGCAGCGGTTATGATGCCTATTGCGCCGATGTTCAGTTCGCA
GGTAAAAGCGGTACCAGTGCCCTGCAGATCTGTCAGGCCCTGGTGGCCAGTGGCATGACCGGTAGTGCCCTGG
CAATTGGTGCCGATACCATTAATCGCAATACCGCCCCGGGTGATCTGACCGAAAGTTATGCCGGTGCAGGCGC
AGCCGCACTGCTGGTTGGTACCGAAAATGTTATTGCCGAGTTCGATGGTAGCTTCAGCTGCGCAGCAGATATTG
CCGATAATATTCGTCCGCAGGGCGATCGCTATATTCGTAGTGGCATGGGCCTGGGCAGCGATAAAAATAGTAT
TGGCCTGGAAGATCAGACCCGCCGCGCCGCAGAAGGCCTGATGGCAAAACTGCATACCAGCGCCGCCGATTAT
GATTATGTTGTGTTCCAGCAGAATCTGGTTAGCACCCCGTATAGTCTGGGTAAACATCTGGGCTTCAATGCCAA
ACAGATTGAACCGGGCATCTATGCAGGTAGCGTTGGCGATACCGGCGCCGCCAGCCCTCTGTTAGGTCTGATT
AATGTGCTGGATCAGGCCCGTCCGGGTCAGAAAATTCTGATGGTTAGTTATGGCTTCGGTGCAGGCAGTGATG
CCATTGCCCTGACCGTGACCGATGCAATTGGTCAGTATCAGAAACGCAATAAACCGCTGCGTCAGCTGCTGGA
AGATAAAACCTATGTGGATTATGGTACCAGTATTAAATACGAGTTCAAATATCTGCGCGCCGATTATGCCCTGA
CCGCATATCTGTAAttacgcctgtctacgcaaggagcATGATCGTTGAAAGTGCCTATAAAGCCATTAAAGATGCAAAAATG
CACCCGCGTGAAATTCAGGGTGTTGCCTATGGTTATCATGGCGAAGGCATTAGTGAATATGGTGGTCTGGGCCC
GACCATTAGTGATGCCCTGGGCATTAGCCCGGCACCGACCTTCATGAGTACCGCCAATTGTACCAGTAGCAGC
GTGAGCTTCCAGATGGGTCATCAGATGGTTGCAAGTGGCGAATATGATATTGTTCTGTGCGGTGGCTTCGAAAA

AATGACCGATCACTTCAATTATGCAGAATATATTGGTAGTAGCACCGAATGCGAATATGATTACTTCCTGGGCA
TTAGTCATACCGATGCCTTCGCACTGGCCACCGCAGAATACTTCCAGAAATTCGGCTATGCCGGTCGTGAAGCA
GATGTGCTGGCAACCTTCGGTCGTCAGATGCGCATCTATGCCCATAATACCCCGACCGCAACCCGTTATGGCCA
GGCAATTCCGACCCTGGAAGCACTGAAAAATAGTGAAGCATGTGGTAGTATGCTGGCCTGGGGTGAAGCAAGT
GGTTGCGCAATTCTGGTTGCCGAACATCTGGCACATAAATATACCGATAAACCGGTGTTCGTTCGCGGCTGCGC
ATATACCGGCGTTAGTCATTACTTCGGTACCCGCTTCCATAATCCGACCCTGCATCATCCGGGTCTGCCGAAAG
ATGTTGGCATGGCAGTGAGCGCAAATAGCATTGCATGTGCCGAAATTGCCTATAAAAAAGCAGGTATTACCCC
GAAAGATATTGATGTGGCCCAGGTGTATGATCTGCTGGGTGCAGGTCTGATTCAGATGGAAAGTATGGGCGTG
TGTGGTAAAGGCCAGGCCGGCGACTTCGTGCTGGAAGGCGGCATTGCCCTGGATGGCCAGCTGCCGCTGAATA
CCGATGGTGGCAATATTGGCCGTGGCCATGCAAGCGGCTGCGATGGCATTCTGCATATTACCGAACTGTTCCGC
CAGCTGCGTGGTGAAGCAGATAATCAGGTGAAAGGTGCACGTATTGGTGTGAGCCAGAATCTGGGCGGCTATG
CCGCACATAATAGTGTTATTGTGCTGAGTAATGATTAAggagcccggaccATGAGCATGTATCCGGAACAGATTCAT
CGCATGACCACCGCAAGTATGCTGCGTGAATGGCGTGAACATGGTGGTAAATATCGCCTGGAAGGCAGCCAGT
GCGAAGAATGCAATGAAATCTTCTTCCCGCGTCGTACCGTGTGTGGCGCATGTAATAGCATGAGTGTTAAACC
GTATCGTTGCGCCCGCACCGGTAAAATTGAAGTTATGGCACATGCAGATAATCCGATTCTGGCAGCCATGGGC
TATGGCGAAACCGTTCCGCGTTGCATGGCAATGATTCGCCTGGATGATGGCCTGGTGATTGCAAGTGAAATTGT
TGATGTGTGTGATGCACAGCAGCTGAAAGTTGGCGCCCCGGTGCGCATGGTTGTTCGTAAACATGTTCGCGAA
AGCAATCTGGCATGGCAGTATGCATATAAATTCGTGCTGGATTAAGGATCCctcgaggtcgacctg-3’

***Bt*ATase** (*phlACB* gene sequence, *Brenneria tiliae*)

phlA
GITSYGFSLPLYRIKVEDIINVWKNTAPDIITQTLGTTSRTVLQPDEDTITLSLDAARQALRRANISTLDAIYLGTCTNP
YDSRSSAAILLEMLGAGKQAFCADIQFSGKSGTSAMQICHAMITSGLANTALAIGADVLSRHVAPGDLTESYAGAA
AAAILFGKKNVIAEIDATFSCAEDLADNIRPQGERYIRSGMGLGSDKNNLGINKHMAFAFHGLLSQTETCKEDYSYV
VFQQPTISIVHTMTKKLGLSNQQTSPALYADTVGDTGASSPMLGLAKILDIAKPGEKILVVSYGFGAGSDAISFTVTN
NILSYRKNHRTVQSVLKEVLWTDYGTATKYEFKFLRPDYALTAYL
phlC
SRRVAIVSVDHTPQPGTCHLEKTFKDLIVESAYRAIGNIDMDPREIQGISFGYHGEGISEYGGLGPTISDALGLSPAPCF
ITASNCTSSSISLQTGCHMVASGEYDIVLCGGFEKMTDHYNYAEYIGSSTESEYDYFLGISHTDAFELCTAEYFHQYG
YQGREADILAKFGHEMRTYAFNTPTSQHYQRPVPSIEKLKNAPGHGALLSWGEASGCAILVAEHLAHRFTDRPVFIK
GAAYSCVSHYYGTRYGNPTLKYPGLPEKINMAISANSIACADIAFRKAGIKASDIDVAQVYDQLGAGLIQIESLGICK
PGQAGDFVLEGGIGINGICPVNTDGGNIGRGHASGCDGIMHVVELFRQLRGESANQVKNARIAVTQNVGGYAAHNS
VIVLTNE
phlB
MSQYPENIHRMTTAGMVRQWREHGGKYRLEGTRCKHCGTISFPRRSVCGNCNKQGLEIYPCSHFGKIETIVDVNNP
ALVIMGYGEVVPRHIATVRLADDITIVTEIVDIIDEYELKPGTEVEMVIRKQVRESNLAWQYAYKF

5’gagaccgcggtcccGAATTCgagctcggtaccATGGGTATTACCAGCTATGGCTTCAGCCTGCCGCTGTATCGCATTAAAGT
GGAAGATATTATTAACGTGTGGAAAAATACCGCACCGGATATTATTACCCAGACCCTGGGCACCACCAGTCGT
ACCGTTCTGCAGCCGGATGAAGATACCATTACCCTGAGCCTGGATGCAGCACGCCAGGCACTGCGCCGCGCAA
ATATTAGTACCCTGGATGCCATCTATCTGGGCACCTGCACCAATCCGTATGATAGCCGTAGCAGCGCCGCCATT
CTGCTGGAAATGCTGGGCGCCGGTAAACAGGCATTCTGCGCCGATATTCAGTTCAGTGGTAAAAGCGGTACCA
GTGCCATGCAGATCTGTCATGCAATGATTACCAGCGGCCTGGCCAATACCGCCCTGGCAATTGGTGCAGATGT
GCTGAGCCGTCATGTTGCCCCGGGCGATCTGACCGAAAGCTATGCAGGTGCAGCAGCAGCCGCAATTCTGTTC
GGCAAAAAAAATGTTATTGCCGAAATTGATGCCACCTTCAGCTGTGCAGAAGATCTGGCAGATAATATTCGTC
CGCAGGGCGAACGCTATATTCGTAGCGGTATGGGTCTGGGCAGCGATAAAAATAATCTGGGCATTAATAAGCA
CATGGCATTCGCATTCCATGGTCTGCTGAGTCAGACCGAAACCTGTAAAGAAGATTATAGTTATGTGGTGTTCC
AGCAGCCGACCATTAGCATTGTGCATACCATGACCAAAAAACTGGGCCTGAGTAATCAGCAGACCAGCCCGGC
CCTGTATGCCGATACCGTTGGCGATACCGGCGCCAGCAGTCCGATGCTGGGCCTGGCTAAAATTCTGGATATTG
CAAAACCGGGCGAAAAAATTCTGGTGGTGAGTTATGGCTTCGGCGCAGGTAGCGATGCCATTAGCTTCACCGT
GACCAATAATATTCTGAGCTATCGTAAAAACCACCGCACCGTTCAGAGTGTTCTGAAAGAAGTGCTGTGGACC
GATTATGGTACCGCAACCAAATATGAGTTCAAATTCCTGCGTCCGGATTATGCCCTGACCGCATATCTGTAAttac
gcctgtctacgcaaggagcATGAGTCGCCGCGTGGCCATTGTGAGCGTGGATCATACCCCGCAGCCGGGCACCTGTCATC
TGGAAAAAACCTTCAAAGATCTGATTGTTGAAAGTGCATATCGCGCCATTGGTAATATTGATATGGACCCTCGC
GAAATTCAGGGCATTAGCTTCGGCTATCATGGTGAAGGCATTAGCGAATATGGTGGTCTGGGCCCGACCATTA
GCGATGCCCTGGGCCTGAGTCCGGCCCCTTGCTTCATTACCGCAAGTAATTGTACCAGCAGTAGTATTAGTCTG
CAGACCGGCTGTCATATGGTTGCAAGCGGCGAATATGATATTGTGCTGTGTGGCGGCTTCGAAAAAATGACCG
ATCATTATAATTACGCAGAATATATCGGCAGTAGCACCGAAAGTGAATATGATTACTTCCTGGGTATTAGTCAT
ACCGATGCATTCGAACTGTGTACCGCCGAATACTTCCATCAGTATGGTTATCAGGGTCGTGAAGCCGATATTCT
GGCCAAATTCGGCCATGAAATGCGTACCTATGCATTCAATACCCCGACCAGCCAGCATTATCAGCGTCCGGTTC
CGAGTATTGAAAAACTGAAAAATGCCCCGGGCCATGGCGCCCTGCTGAGTTGGGGTGAAGCCAGTGGCTGCGC
CATTCTGGTTGCAGAACATCTGGCACATCGCTTCACCGATCGCCCGGTGTTCATTAAAGGTGCCGCATATAGCT
GTGTGAGTCATTATTATGGTACCCGTTATGGTAATCCGACCCTGAAATATCCGGGCCTGCCGGAAAAAATTAAT
ATGGCAATTAGTGCCAACAGCATTGCATGTGCCGATATTGCATTCCGCAAAGCCGGTATTAAAGCCAGCGATA
TTGATGTTGCCCAGGTGTATGATCAGCTGGGCGCCGGCCTGATTCAGATTGAAAGCCTGGGCATCTGTAAACCG
GGTCAGGCAGGCGACTTCGTTCTGGAAGGCGGCATTGGTATTAATGGTATCTGTCCGGTTAATACCGATGGTGG

CAATATTGGTCGCGGCCATGCAAGTGGTTGTGATGGTATTATGCATGTTGTTGAACTGTTCCGTCAGCTGCGTG
GTGAAAGCGCAAATCAGGTGAAAAATGCCCGTATTGCCGTTACCCAGAATGTTGGTGGTTATGCCGCACATAA
TAGTGTGATTGTGCTGACCAATGAATAAggagcccggaccATGAGTCAGTATCCGGAAAATATTCATCGTATGACCA
CCGCAGGCATGGTGCGTCAGTGGCGTGAACATGGTGGTAAATATCGTCTGGAAGGCACCCGTTGCAAACATTG
TGGCACCATTAGCTTCCCGCGTCGTAGCGTGTGCGGCAATTGCAATAAACAGGGCCTGGAAATCTATCCGTGC
AGCCACTTCGGTAAAATTGAAACCATTGTGGATGTTAATAACCCGGCCCTGGTTATTATGGGTTATGGTGAAGT
TGTTCCGCGCCATATTGCCACCGTTCGTCTGGCAGATGATATTACCATTGTTACCGAAATTGTTGATATCATTGA
CGAATATGAGCTGAAACCGGGCACCGAAGTGGAAATGGTTATTCGCAAACAGGTTCGTGAAAGCAATCTGGCC
TGGCAGTATGCCTATAAATTCTAAGGATCCctcgaggtcgacctg-3’

***Va*ATase** (*phlACB* gene sequence, *Vibrio aerogenes*)

phlA
GITGYGYSLPFLRLSVEQTTEVWKNTPLALLKNGLKVSSRTVLQPDEDTITLAADAARQALKQHLKQQARFPDISPE
AIYLGTCTNPYQSRSSAAIVAEMLGLSRQLFCADIQFAGKSGTSALQICHALINSGMCHQALAIGSDTMNRHTAPGD
LTESYAGAGAVAMLLGTERVIARIDNTGSGCEDLADNIRPEGERYIRSGMSLGSDKNNLGIYRHMQLSGEQLLHDM
DCTWADFDFVVVQQPTESAVDTMTEKLQLEYAQTVHSRYAATTGDIGSASPLMGLAATLDHAQPGQKILMLSYGF
GAGSDAIALTVTDEILPYREQAPTVAALLARTKEVSYAEAAKYEFKFSRPDYALAPYL
phlC
RRVAIVAAEHTTNPGIYHPEITFKEMIAESAYRAIKRIDLDPKMIQGLSYGYHGEGVSEYGGLGPTISDALGLSPAPCF
ITASNCTSGSVSFQMGVQMIESGRYDIVLCGGFEKPSDHLNYTDYINASTETEYDYLMGISHVDAFHLANEAYFHYY
GYSADDTAEVLARFGLQMRRYGQQCPDSFYYQKSLPSVEHLKSLPEHGAMLAPGEASGSVILVAEELAHRYTDKP
VFVKGMAYTNTSHYFGSRYNRTAVRGIDHASASGMVSPANAIACTTAAYQDAGITAEDVDVIQVYDEQAAGLIQM
ETLGICPAGEAGRWVLDDQIGPDGRCPVNTDGGNIGRGHASGCDGIMHIAELFRQLRGESANQVSHARIAVSSNIGG
YMAHNSVIVLCNE
phlB
MSQYPEQIHRLNTASMLREWREHGGKYRLEGTRCQHCGATFFPRRAVCSHCHESGLEVCRFPHEGTIEVMQDADIP
VLALMGYGEMMPRHIAMIRLTDDIVIAGEIVDVASEDELEPGARVEMVVRKQVRESNLAWQYAYKF

5’gagaccgcggtcccGAATTCgagctcggtaccATGGGTATTACCGGCTATGGCTATAGTCTGCCGTTCCTGCGTCTGAGCGT
TGAACAGACCACCGAAGTGTGGAAAAATACCCCGCTGGCCCTGCTGAAAAATGGTCTGAAAGTGAGTAGCCGT
ACCGTGCTGCAGCCGGATGAAGATACCATTACCCTGGCCGCCGATGCAGCACGTCAGGCACTGAAACAGCATC
TGAAACAGCAGGCACGCTTCCCGGATATTAGTCCGGAAGCCATCTATCTGGGTACCTGCACCAATCCGTATCA
GAGCCGTAGCAGCGCAGCCATTGTTGCAGAAATGCTGGGTCTGAGTCGCCAGCTGTTCTGCGCAGATATTCAG
TTCGCCGGCAAAAGTGGTACCAGCGCACTGCAGATCTGTCATGCACTGATTAATAGCGGCATGTGCCATCAGG
CACTGGCCATTGGCAGTGATACCATGAATCGCCATACCGCACCGGGCGATCTGACCGAAAGCTATGCAGGCGC
AGGTGCAGTTGCAATGCTGCTGGGCACCGAACGTGTGATTGCCCGTATTGATAATACCGGCAGTGGTTGTGAA
GATCTGGCCGATAATATTCGCCCGGAAGGTGAACGCTATATTCGTAGCGGCATGAGTCTGGGCAGTGATAAAA
ATAATCTGGGCATCTATCGTCATATGCAGCTGAGCGGTGAACAGCTGCTGCATGATATGGATTGCACCTGGGCC
GACTTCGACTTCGTGGTGGTTCAGCAGCCGACCGAAAGCGCCGTTGATACCATGACCGAAAAACTGCAGCTGG
AATATGCCCAGACCGTGCATAGTCGTTATGCCGCAACCACCGGTGATATTGGCAGTGCCAGTCCGCTGATGGG
TCTGGCCGCCACCCTGGATCATGCACAGCCGGGTCAGAAAATTCTGATGCTGAGCTATGGCTTCGGTGCAGGC
AGCGATGCCATTGCACTGACCGTGACCGATGAAATTCTGCCGTATCGCGAACAGGCACCGACCGTGGCAGCAC
TGCTGGCCCGTACCAAAGAAGTGAGCTATGCCGAAGCCGCAAAATATGAGTTCAAATTCAGTCGCCCGGATTA
TGCACTGGCCCCGTATCTGTAAttacgcctgtctacgcaaggagcATGCGCCGTGTGGCCATTGTGGCCGCCGAACATACCA
CCAATCCGGGTATCTATCATCCGGAAATTACCTTCAAAGAAATGATTGCCGAAAGTGCATATCGCGCAATTAA
ACGCATTGATCTGGACCCTAAAATGATTCAGGGTCTGAGTTATGGTTATCATGGTGAAGGCGTGAGTGAATAT
GGTGGCCTGGGTCCGACCATTAGTGATGCACTGGGTCTGAGCCCGGCACCGTGCTTCATTACCGCCAGCAATTG
TACCAGCGGTAGTGTTAGCTTCCAGATGGGTGTGCAGATGATTGAAAGTGGTCGCTATGATATTGTGCTGTGCG
GCGGCTTCGAAAAACCGAGCGATCATCTGAATTATACCGATTATATTAACGCCAGCACCGAAACCGAATATGA
TTATCTGATGGGTATTAGTCATGTTGATGCATTCCATCTGGCCAATGAAGCCTACTTCCATTATTATGGTTATAG
TGCAGATGATACCGCAGAAGTGCTGGCACGCTTCGGCCTGCAGATGCGCCGTTATGGTCAGCAGTGCCCGGAT
AGCTTCTATTATCAGAAAAGCCTGCCGAGCGTGGAACATCTGAAAAGCCTGCCTGAACATGGTGCCATGCTGG
CACCGGGCGAAGCAAGCGGCAGCGTTATTCTGGTGGCAGAAGAACTGGCACATCGCTATACCGATAAACCGGT
GTTCGTGAAAGGTATGGCATATACCAATACCAGCCATTACTTCGGTAGCCGCTATAATCGTACCGCCGTGCGCG
GTATTGATCATGCAAGTGCAAGTGGCATGGTGAGTCCGGCCAATGCAATTGCCTGTACCACCGCAGCCTATCA
GGATGCCGGCATTACCGCAGAAGATGTTGATGTGATTCAGGTGTATGATGAACAGGCAGCCGGCCTGATTCAG
ATGGAAACCTTAGGTATCTGTCCGGCAGGTGAAGCCGGCCGTTGGGTTCTGGATGATCAGATTGGCCCGGATG
GCCGTTGCCCGGTTAATACCGATGGTGGTAATATTGGTCGCGGTCATGCCAGTGGTTGCGATGGTATTATGCAT
ATTGCAGAACTGTTCCGCCAGCTGCGCGGTGAAAGCGCAAATCAGGTGAGCCATGCCCGCATTGCAGTGAGCA
GTAATATTGGCGGTTATATGGCCCATAATAGTGTGATTGTTCTGTGTAATGAATAAggagcccggaccATGAGCCAGT
ATCCGGAACAGATTCATCGCCTGAATACCGCAAGCATGCTGCGCGAATGGCGTGAACATGGCGGCAAATATCG
CCTGGAAGGTACCCGTTGCCAGCATTGTGGTGCCACCTTCTTCCCGCGTCGCGCAGTGTGTAGCCATTGTCATG
AAAGTGGTCTGGAAGTGTGTCGCTTCCCGCATGAAGGTACCATTGAAGTTATGCAGGATGCCGATATTCCGGTT
CTGGCCCTGATGGGTTATGGTGAAATGATGCCGCGCCATATTGCCATGATTCGTCTGACCGATGATATTGTGAT
TGCCGGTGAAATTGTTGATGTTGCCAGTGAAGATGAACTGGAACCGGGTGCACGTGTTGAAATGGTGGTGCGC
AAACAGGTTCGTGAAAGTAATCTGGCCTGGCAGTATGCCTATAAATTCTAAGGATCCctcgaggtcgacctg-3’

***Tp*ATase** (*phlACB* gene sequence, *Thermofilum pendens*)

phlA
MPGIASILGYGAYIPVYRIESGEISRVHTKGGEKAPVKQKSVPGPDEDSLTMAYEASKNALKRARLDPREVQALYIG
SESPPYAVKPSATVVAEALGLSRKLYGIDMEFACKAGTTALISVAGLVKSGIITYGLAVGTDTAQGRPGDELEYTAG
AGAAAFVVSPVRSDAVATIEHVYSYVTDTPDFWRREGERFPMHTFRFTGEPAYFHHIVSAAKGLFEETGLKPSDFAY
AVFHQPNVKFPQRVGAMLGFKPEQLKLGLLSGEIGNTYAAASLIGLTNVLDHAKPGERILVVSFGSGAGSDALSIVV
EEGIEDRRGLAPLTMQYVKRAKLVDYAVYLKYKDFIKR
phlC
MTGVYLVGIGATKIDEHWERPLRDLMLEASLKALRDAGLSKRDVEAIFVGNMSSGYLQGQEHLGSLLATWLGVPG
VAANKVEAACGSGGAAFHNAFLAVKSGLYDCVLAVGVEKLTDATTPDATSALIMAEDQEYVAFAGFSFVALNAL
VYKAYMKKYGAKQEDIALFAVHDHKYAVNNPLAQYPRAVTLEEVLSSPMVADPLRLLESAPLGDGAAAALLCSE
KKLKEVEKDVVIEVVGSALATDVLSLHDRADLTTLSATVKASRKAYAMAGIEPKDVDVLEVHDAFTVLGVIHLED
LGFAEKGAGWKLLKEGQLEKDGDLPTNTMGGLKARGHPVGATGIYQIYDIAVQLRGEAGKNQVDGAEVGLAQNV
GGVGGTVSVNILKRVR
phlB
SVPRAWRERKIKYQLIGGKCKDCGKSFYPYRQVCPACGSENVEEVKLPERGVVEVFTVVRSPPSDFAWQAPYVVAL
VRLEDGTLVPAQITDVDPDEVHEGMEVEAVFRKYREQGQQGIIEYGIKF

5’gagaccgcggtcccGAATTCgagctcggtaccATGCCGGGTATTGCAAGTATTCTGGGCTATGGTGCCTATATTCCGGTGTA
TCGTATTGAAAGTGGCGAAATTAGCCGTGTTCATACCAAAGGTGGCGAAAAAGCCCCGGTTAAACAGAAAAGT
GTTCCGGGTCCGGATGAAGATAGCCTGACCATGGCCTATGAAGCAAGCAAAAATGCACTGAAACGCGCACGTC
TGGACCCTCGCGAAGTTCAGGCCCTGTATATTGGTAGCGAAAGTCCGCCGTATGCCGTTAAACCGAGCGCCAC
CGTGGTTGCCGAAGCACTGGGCCTGAGTCGTAAACTGTATGGCATTGATATGGAGTTCGCATGTAAAGCAGGT
ACCACCGCACTGATTAGTGTTGCCGGTCTGGTTAAAAGTGGCATTATTACCTATGGCCTGGCAGTTGGCACCGA
TACCGCACAGGGTCGTCCGGGCGATGAACTGGAATATACCGCCGGCGCAGGTGCAGCAGCATTCGTTGTTAGT
CCGGTGCGCAGCGATGCAGTGGCCACCATTGAACATGTGTATAGTTATGTTACCGATACCCCGGACTTCTGGCG
CCGTGAAGGCGAACGCTTCCCGATGCATACCTTCCGCTTCACCGGTGAACCGGCCTACTTCCATCATATTGTTA
GCGCAGCCAAAGGTCTGTTCGAAGAAACCGGCCTGAAACCGAGTGACTTCGCATATGCAGTGTTCCATCAGCC
GAATGTGAAATTCCCGCAGCGCGTGGGTGCAATGCTGGGCTTCAAACCGGAACAGCTGAAACTGGGCCTGCTG
AGCGGCGAAATTGGCAATACCTATGCAGCAGCCAGTCTGATTGGTCTGACCAATGTGCTGGATCATGCAAAAC
CGGGTGAACGCATTCTGGTGGTGAGCTTCGGCAGTGGCGCAGGCAGCGATGCACTGAGTATTGTGGTGGAAGA
AGGTATTGAAGATCGTCGCGGCCTGGCACCGCTGACCATGCAGTATGTTAAACGTGCAAAACTGGTGGATTAT
GCAGTGTATCTGAAATATAAAGACTTCATTAAGCGCTAAttacgcctgtctacgcaaggagcATGACCGGCGTGTATCTGGT
GGGTATTGGCGCAACCAAAATTGATGAACATTGGGAACGTCCGCTGCGCGATCTGATGCTGGAAGCAAGCCTG
AAAGCACTGCGTGATGCCGGTCTGAGCAAACGTGATGTTGAAGCCATCTTCGTTGGTAATATGAGCAGCGGTT
ATCTGCAGGGTCAGGAACATCTGGGTAGTCTGCTGGCCACCTGGCTGGGCGTTCCGGGTGTTGCCGCCAATAA
AGTGGAAGCAGCATGTGGTAGTGGCGGTGCAGCCTTCCATAATGCATTCCTGGCCGTTAAAAGTGGTCTGTAT
GATTGCGTTCTGGCAGTTGGTGTTGAAAAACTGACCGATGCAACCACCCCGGATGCCACCAGTGCCCTGATTAT
GGCAGAAGATCAGGAATATGTTGCCTTCGCAGGCTTCAGCTTCGTTGCCCTGAATGCCCTGGTGTATAAAGCCT
ATATGAAAAAATACGGCGCAAAACAGGAAGATATTGCACTGTTCGCCGTTCATGATCATAAATATGCCGTTAA
TAACCCGCTGGCCCAGTATCCGCGCGCAGTTACCCTGGAAGAAGTTCTGAGTAGTCCGATGGTGGCCGATCCG
CTGCGCCTGCTGGAAAGTGCCCCGCTGGGTGATGGTGCCGCCGCAGCACTGCTGTGTAGCGAAAAAAAACTGA
AAGAAGTTGAAAAGGACGTGGTGATTGAAGTTGTGGGTAGCGCACTGGCCACCGATGTGCTGAGTCTGCATGA
TCGCGCCGATCTGACCACCCTGAGCGCCACCGTTAAAGCAAGTCGTAAAGCATATGCCATGGCAGGTATTGAA
CCGAAAGATGTGGATGTTCTGGAAGTGCATGATGCCTTCACCGTTCTGGGCGTTATTCATCTGGAAGATCTGGG
CTTCGCAGAAAAAGGTGCAGGTTGGAAACTGCTGAAAGAAGGTCAGCTGGAAAAAGATGGTGATCTGCCGAC
CAATACCATGGGTGGTCTGAAAGCCCGTGGCCATCCGGTGGGCGCAACCGGTATCTATCAGATCTATGATATT
GCAGTGCAGCTGCGCGGCGAAGCAGGTAAAAATCAGGTTGATGGCGCCGAAGTGGGTCTGGCCCAGAATGTT
GGTGGTGTTGGTGGCACCGTTAGCGTTAATATTCTGAAACGCGTGCGCTAAggagcccggaccATGAGTGTGCCGCG
CGCATGGCGTGAACGCAAAATTAAATATCAGCTGATTGGTGGTAAGTGCAAAGATTGCGGCAAAAGCTTCTAT
CCGTATCGCCAGGTGTGCCCGGCCTGCGGTAGTGAAAATGTGGAAGAAGTTAAACTGCCGGAACGCGGCGTTG
TTGAAGTGTTCACCGTGGTGCGCAGTCCGCCGAGTGACTTCGCCTGGCAGGCACCGTATGTGGTGGCCCTGGTG
CGTCTGGAAGATGGCACCCTGGTGCCGGCCCAGATTACCGATGTGGACCCTGATGAAGTGCATGAAGGCATGG
AAGTGGAAGCAGTGTTCCGCAAATATCGTGAACAGGGCCAGCAGGGTATTATTGAATATGGTATTAAATTCTA
AGGATCCctcgaggtcgacctg-3’

1.4. Plasmid construction and design of *Cs*ATase

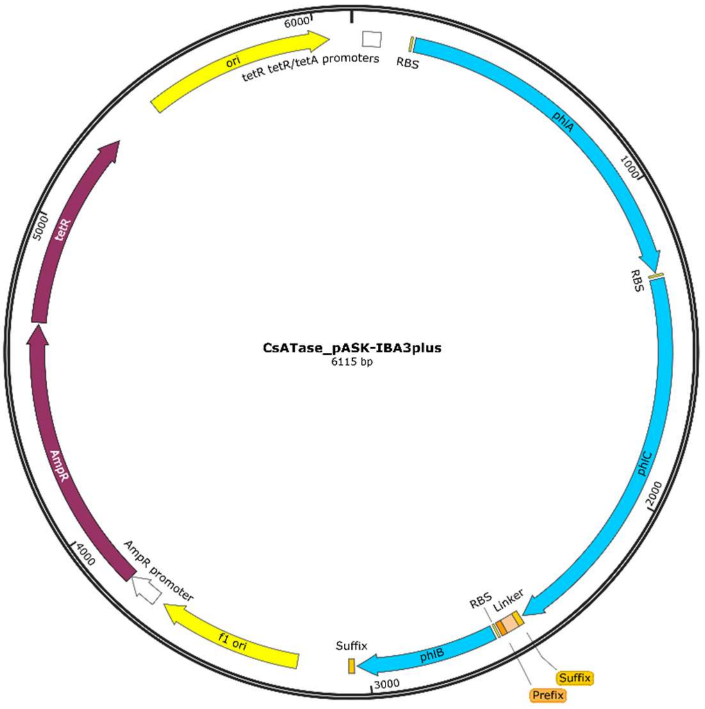

Figure S1. Plasmid map of *Cs*ATase in pASK-IBA3plus.

Table S4. Gene fragments introduced to the plasmid of *Cs*ATase

Gene fragments	Sequence (5'- 3')
Suffix	cacatgctcggctcagtc
Linker	gcctgcggatgcatggtctggatgtgccgaggtctgcgtcg
Prefix	gtgcagactcagtcgacg

1.5. Mutagenesis of *Cs*ATase

The following reagents were assembled in a thin-walled PCR tube.

Table S5. PCR Mix

PCR Mix	
Nuclease free water	77.5
5x Phusion GC buffer	25
10 mM dNTP	2.5
10 µM forward primer	6.25
10 µM reverse primer	6.25
10 ng/µL template DNA	2.5
3% DMSO	3.75
Phusion DNA polymerase	1.25
Total volume	125 µL (5x25 µL)

The reactions were mixed completely and 25 µL was aliquoted to thin-walled PCR tubes. A gradient method (60.3 °C, 62 °C, 64.4 °C, 66.8 °C, 69.7 °C) was used in the thermocycler and the following cycling condition was performed.

Table S6. Thermocycling conditions for a PCR

98 °C	30 sec	
98 °C	10 sec	29x
gradient	20 sec	
72 °C	3 min	
72 °C	5 min	
4 °C	Hold	

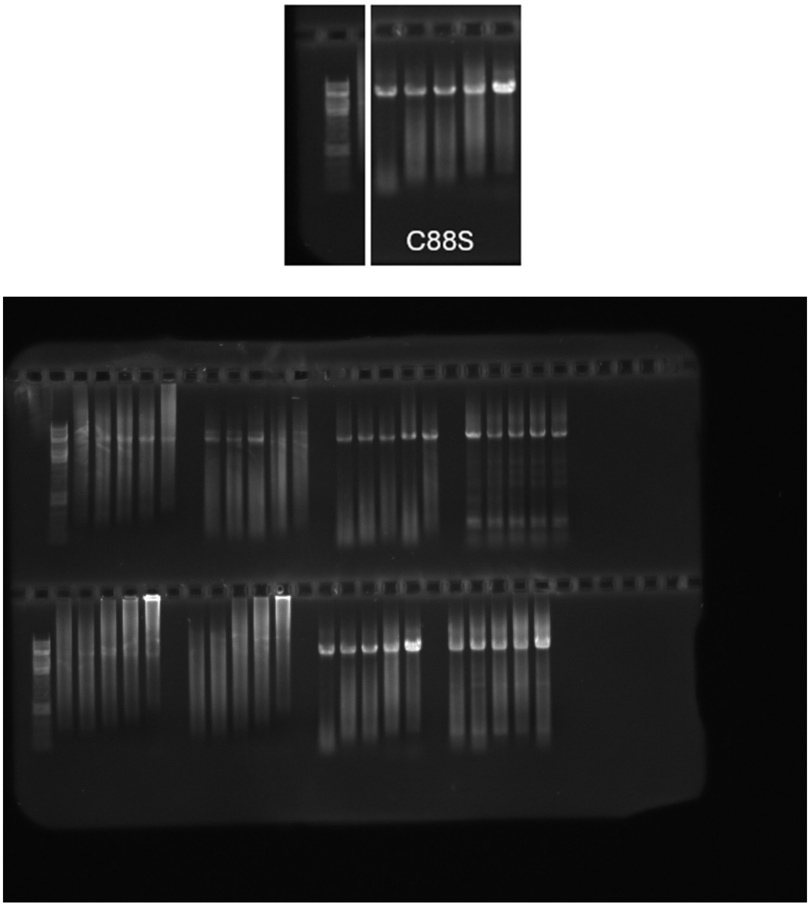

Figure S2. Top: Agarose gel (1%) after PCR reaction for the C88S variant [cropped image for easier readability. The parts shown are taken from the full-size image below, bottom row, third block (lane 15-19) and the marker at the beginning of the row (lane 2)]. Bottom: Uncropped/full-size image.

The reactions with a visible band (**Figure S2**) were pooled together and DpnI (1 µL) was added to the mixture which was subsequently incubated at 37 °C overnight (300 rpm). The reaction was cleaned up with a Clean-up Kit (Macherey-Nagel) and the concentration was measured using NanoDrop 2000 Spectrophotometer (Thermo Fisher Scientific, Waltham, USA). For the ligation reaction it was calculated that in the reaction the DNA should be diluted to approx. 50 ng/µL and the following reaction was set up.

Table S7. KLD reaction

KLD reaction	Volume (µL)
Nuclease free water	x
10x T4 Ligase buffer	2
T4 Ligase	1
DNA (total 50 ng/µL)	x
Total volume	20 µL

The reactions were incubated overnight at room temperature. Subsequently, 2 µL of the reaction mixture was transformed into *E. coli* DH5α cells for plasmid storage. After sequence confirmation, the verified plasmid was transformed into *E. coli* BL21(DE3) cells for protein expression.

1.6. Shake flask cultivation and expression of the recombinant acyltransferases (ATases)

LB-medium (1 L containing 10 g/L tryptone, 5 g/L yeast extract, 5 g/L NaCl) supplemented with ampicillin (100 µg/mL) was inoculated with the overnight culture (10 mL) and incubated (INFORS HT Multitron Standard incubation shaker) at 37 °C and 135 rpm until the OD_{600} (Eppendorf BioPhotometer) reached 0.7. The protein expression was induced by addition of anhydrotetracycline (AHTC, 200 µg/L) and incubation was continued for ATases overnight at 30 °C. The cells were harvested by centrifugation (25 min, 8000 rpm) and washed once with KPi buffer (50 mM, pH 7.5). The KPi-treated pellet was weight and resuspended again in KPi buffer (7 mL to 1 g wet cells) followed by ultrasonification (40% amplitude, 8 min, pulse 1 sec, pause 4 sec, Branson Ultrasonics™ Sonifier™ SFX250 Cell Disruptor). After centrifugation (25 min, 18000 rpm), the supernatant was aliquoted and shock-frozen in liquid nitrogen. The aliquots were stored at -20 °C until use for biotransformations.

The total protein concentration of cell free extracts was measured with Bradford protein assay. First, a calibration curve was prepared by dissolving BSA in eight different concentrations (2 mg/mL, 1.5 mg/mL, 1 mg/mL, 0.75 mg/mL, 0.5 mg/mL, 0.25 mg/mL, 0.125 mg/mL, 0.025 mg/mL) in KPi buffer (50 mM 7.5 pH). All protein samples (20 µL) were mixed with commercial Bradford working reagent (1000 µL) in cuvettes. A blank was prepared using a cuvette filled with water, and the color was allowed to develop for 10 min. Absorbance measurements were taken at 595 nm using a BioPhotometer from Eppendorf, and the calibration was saved in the instrument. The unknown samples were diluted, and 20 µL of each diluted sample was mixed with 1000 µL Bradford working reagent. The color was allowed to develop for 10 min and the absorbance measurements were taken using the calibrated BioPhotometer. The total protein contents were determined as mean concentrations of the measured dilutions.

The cell free extract was analyzed on commercially available 10% or 12% Bis-Tris SDS-PAGE gel. The samples were diluted with distilled water and they were mixed with Laemmli sample buffer (1:1). Prior to uploading on the gel, the samples were heated at 95 °C for 5 min.

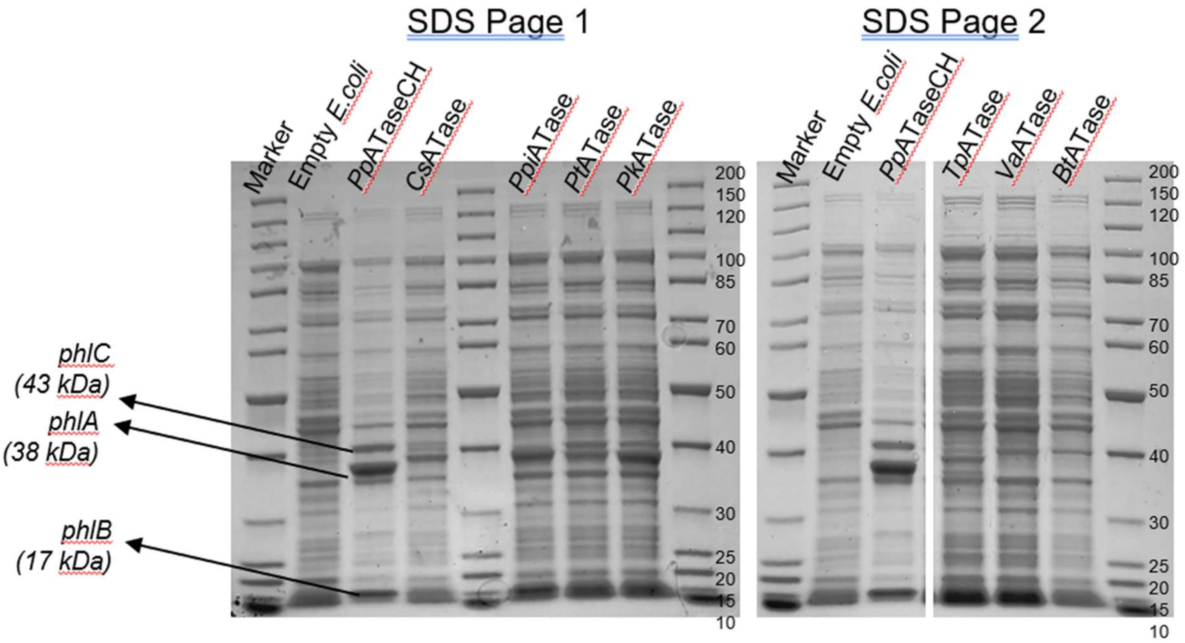

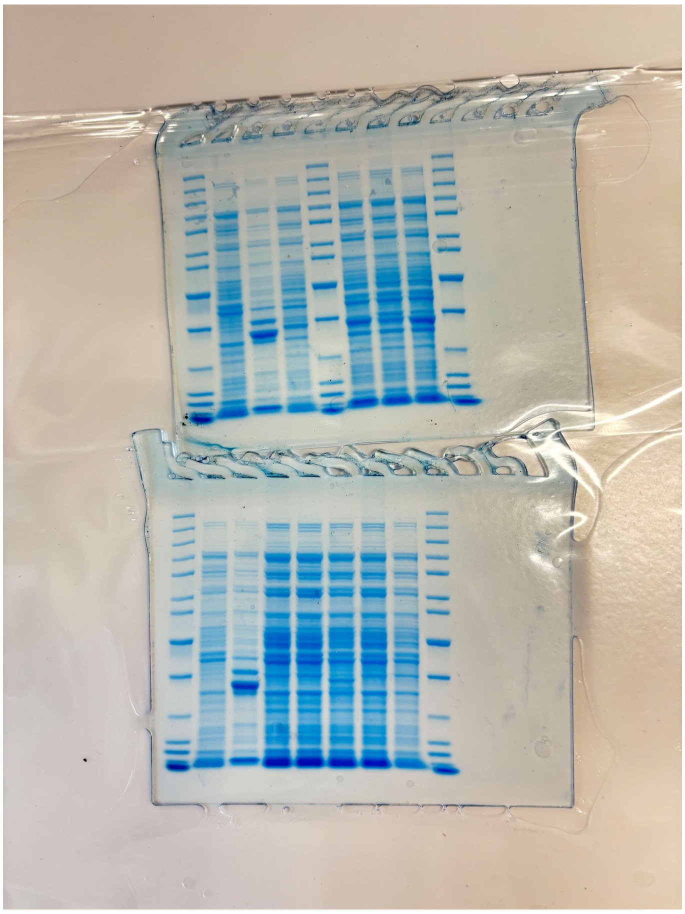

Figure S3. Top: SDS PAGEs of cell free extracts of the explored ATases in this study [cropped image for easier readability. The parts shown are taken from the full-size image below]. Bottom: Uncropped/full-size image.

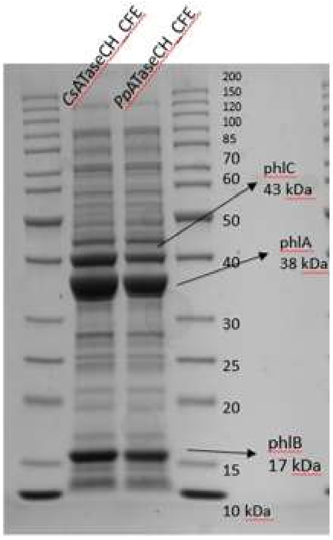

Figure S4. SDS Page gel of CFE of *Cs*ATase (improved expression) and *Pp*ATaseCH.

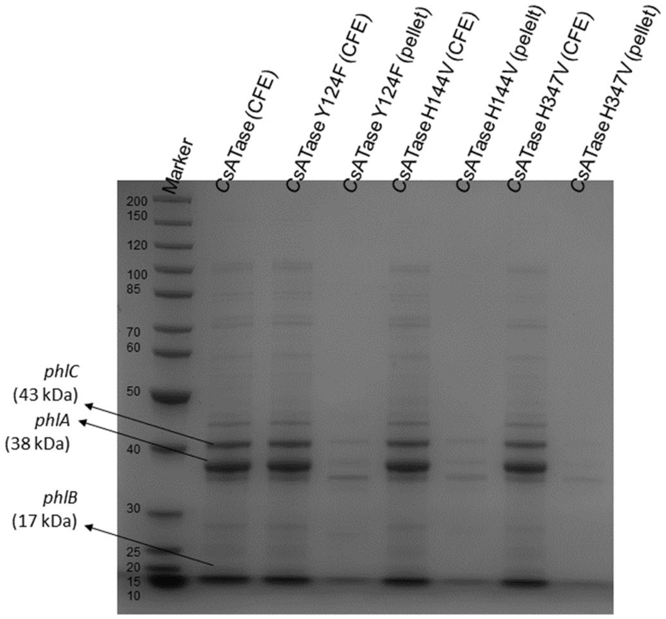

Figure S5. SDS Page gel of *Cs*ATase variants.

1.7. Purification of *Cs*ATase and its variant

Purification of *Cs*ATase and *Cs*ATase C88S was achieved by directly using size-exclusion chromatography with a Superdex 200 16/600 HiLoad-column. The column was initially washed with water (250 mL), followed by conditioning (potassium phosphate buffer, 50 mM, pH 7.5, 100 mM NaCl, 250 mL) with a flow-rate of 0.5 mL/min. The cell-free extract (3.5 mL ≡ 0.5 g wet cells) was filtered (0.45 µm) prior to loading onto the column. *Cs*ATase was eluted after 60 mL with a flow-rate of 0.75 mL/min. The purity of the fractions was estimated by SDS-PAGE (Figure S6, Figure S7). All enzyme-containing fractions were combined and concentrated to approximately 2.5 mL with a Vivaspin column (MWCO 30,000). NaCl was removed by filtration through a PD-10-desalting column (final buffer = potassium phosphate buffer, 50 mM, pH 7.5) and the enzyme solution was concentrated with a Vivaspin column (MWCO 30,000) to 13 mg/mL. Protein concentrations were measured with Bradford assay and initial rates were calculated from the activity assay to determine the batch activity.

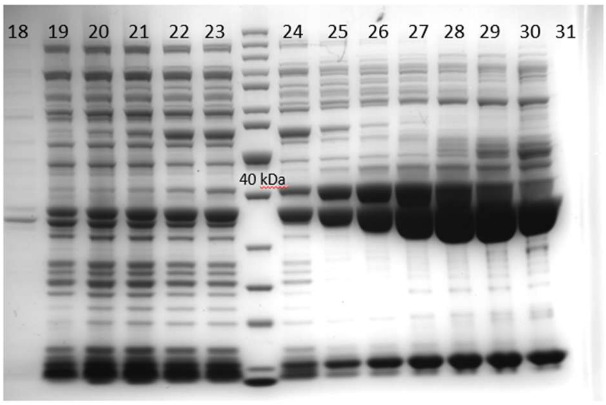

Figure S6. SDS Page gel (12%) of the fractions obtained after size-exclusion purification of *Cs*ATase.

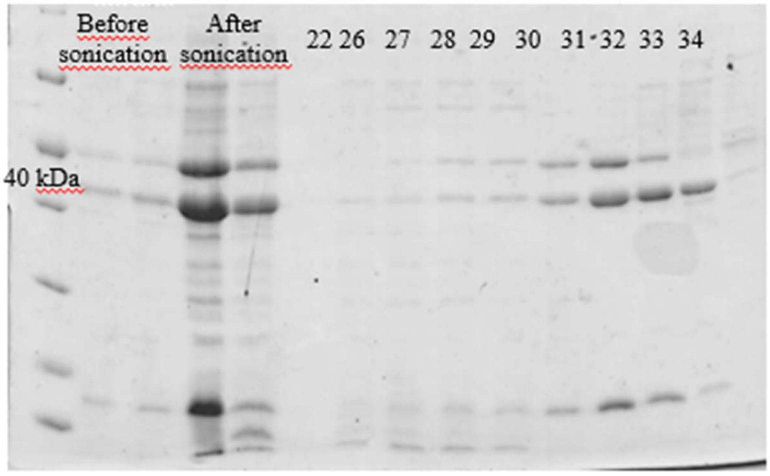

Figure S7. SDS Page gel (12%) of the fractions obtained after size-exclusion purification of *Cs*ATase C88S.

2. Analytical methods

TLC was carried out with pre-coated aluminum sheets (TLC Silica gel 60 F254, Merck) with detection by UV (254 nm) and/or by staining with cerium molybdate solution (0.02 M ammonium molybdate, 0.02 M cerium (IV) sulfate in 1 M sulfuric acid) or potassium permanganate solution (0.047 M potassium permanganate, 0.36 M potassium carbonate and 0.031 M sodium hydroxide in water).

^{1}H- and ^{13}C-NMR spectra were recorded at 20 °C on a Bruker NMR operating at 300 and 75 MHz, respectively.

The HPLC-TOF-MS qualitative analyses were performed using High-Performance Liquid Chromatograph Agilent 1260 Infinity Series coupled with Agilent 6230 TOF LC/MS mass spectrometer equipped with an electrospray ion source (ESI). Parameters for analysis were set using positive ion mode as follows: capillary voltage 1600 V; nebulizer pressure 40 psig; drying gas 5 L/min; gas temperature 300 °C; fragmentor voltage: 150 V; skimmer voltage: 65 V; octopole 1 RF Vpp 750 V; nozzle voltage 2000 V; with direct flow injection (no column). The isocratic flow 0.3 mL/min was applied and the mobile phase was 60% H_2O (0.1% 5 M ammonium formate) and 40% [ACN 0.9:H_2O 0.1 (0.1% 5 M ammonium formate)]; injection volume 1 µL.

The HPLC-MS analyses were performed using High-Performance Liquid Chromatograph Agilent 1290 Infinity Series coupled with Agilent 6100 Series Quadrupole LC/MS mass spectrometer equipped with an electrospray ion source (ESI). Parameters for analysis were set using negative ion mode as follows: capillary voltage 4000 V; nebulizer pressure 15 psig; drying gas 7 L/min; gas temperature 300 °C; fragmentor voltage: 135 V; with a reverse stationary phase column (Luna 5 µm C18 (2) 100 Å 250 × 4.6 mm). The method was run using H_2O (+formic acid, 0.1% v/v, eluent A) and acetonitrile (+formic acid, 0.1% v/v, eluent B) as elution solvents over 18 minutes with 0,8 mL/min flow rate. The injection volume was 5 µL. The column temperature was 30 °C and the compounds were UV detected at 254 nm. Reaction products were quantified at 254 from the peak areas on the basis of standard curves with reference compounds.

Table S8. *The following eluent ratio was used in the case of every formyl acceptor except **1f***

Time [min]	B [%] [acetonitrile (+TFA 0.1% v/v)]
0	35
13	90
15	35
18	35

Table S9. *The following eluent ratio was used in the case of **1,3f***

Time [min]	B [%] [acetonitrile (+TFA 0.1% v/v)]
0	55
13	90
15	55
18	55

2.1. Activity assay

The specific activity was defined as the number of micromoles of 2,4-dihydroxybenzaldehyde (**3a**) formed per minute from the formylation of resorcinol (**1a**) by 1 mg of enzyme preparation at 30 °C, under the assay conditions described below.

The activity of acyltransferases was tested by monitoring spectrophotometrically the absorbance of **3a** at 320 nm using a Molecular Devices SpectraMax M2 Microplate Reader. The enzyme activity was assessed during the formylation reaction of **1a** with **2a** to give **3a**. Calibration solutions for **3a** with total volumes of 200 µL were prepared in triplicates in the concentrations 0 µM, 3 µM, 6.5 µM, 12.5 µM, 25 µM, 50 µM and 100 µM. Besides **3a**, the solutions contained KPi buffer (100 mM, pH 7.5) and 10% DMSO (v/v). Reaction solutions with total volumes of 200 µL were prepared in triplicates containing

KPi buffer (140 µL, 100 mM, pH 7.5), **1a** solution [20 µL from 0.5 mM stock solution dissolved in KPi buffer (100 mM, pH 7.5), 0.05 mM final concentration], **2a** (20 µL from 5 mM stock solution dissolved in DMSO, 0.5 mM final concentration) and acyltransferase (20 µL from 1 µg/µL stock solution dissolved in KPi buffer, 0.1 µg/µL final concentration). Blanks were prepared for each sample containing 20 µL additional buffer but no **1a**. Real-time absorbance measurement at 320 nm was carried out at 30 °C for 10 min and started immediately after the initiation of the reaction by the addition of **2a**.

Table S10. Typical ATase batch activities

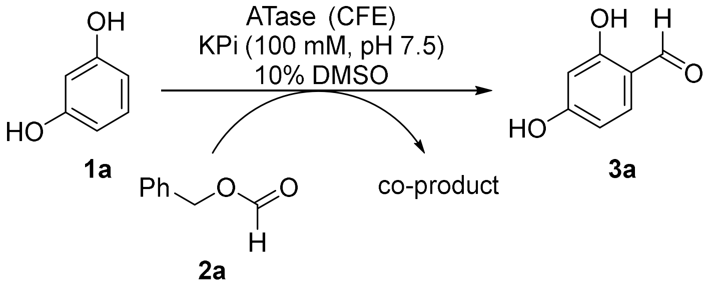

ATase	Specific activity [mU mg^{-1}]
*Pp*ATaseCH (CFE)	22.3±1.6[a]
*Cs*ATase (CFE)	30.1±1.5[a]
*Cs*ATase C88S (CFE)	n.d.
*Cs*ATase Y124F (CFE)	n.d.
*Cs*ATase H144V (CFE)	n.d.
*Cs*ATase H347V (CFE)	n.d.
*Pp*ATaseCH (purified)	12.3±2.7[b]
*Cs*ATase (purified)	15.2±1.0[b]

n.d.= not detected, [a] mg refers to CFE, [b] mg refers to purified enzyme

3. Reaction optimization and substrate scope: Procedure and structural data

General procedure 1: Bioformylation of 1a using 2a catalyzed by ATases

Resorcinol [125 µL of 40 mM stock solution dissolved in KPi buffer (100 mM, pH 7.5), 5 mM final concentration] was mixed with KPi buffer (100 mM, pH 7.5, volume adapted to total volume of reaction 1 mL). Phenyl formate (5.5 µL, 50 mM final concentration) was added to the mixture and the bioformylation (total volume 1 mL) was started by addition of the CFE of *Pp*ATaseCH (164 µL, 60 mU/mL) and the same volume in the case of homologue enzymes. The reaction mixture was shaken for 18 h at 35 °C and 900 rpm in an orbital shaker. Samples (100 µL) were taken after 18 h and were quenched by the addition of acetonitrile (1:1 v/v). The precipitated protein was removed by centrifugation (10 min, 14,000 rpm) and the clear supernatant was injected to HPLC for determination of conversion. All reactions were performed in duplicate and as a negative control, reactions without CFE were performed.

General Procedure 2: Bioformylation of 1a using different formyl donors

Resorcinol [250 µL of 40 mM stock solution in KPi buffer (100 mM, pH 7.5), 10 mM final concentration] was mixed with KPi buffer (100 mM pH 7.5, volume adapted to total volume of reaction 1 mL). Different formyl donors (**2a-d**, 50 mM final concentration) were added to the mixture and the bioformylation (total volume 1 mL) was started by addition of the CFE of recombinant acyltransferase (60 mU/mL). The reaction mixture was shaken for 18 h at 35 °C and 750 rpm in an orbital shaker. Samples (100 µL) were taken after 18 h and were quenched by the addition of acetonitrile (1:1 v/v). The precipitated protein was removed by centrifugation (10 min, 14,000 rpm) and the clear supernatant was injected to HPLC for conversion determination. All reactions were performed in duplicate and as a negative control, reactions without CFE were performed.

General Procedure 3: Hydrolysis study of phenyl formate

Phenyl formate (5.5 µL, 50 mM) was dissolved in KPi buffer (100 mM, pH 7.5). CFE of *Pp*ATaseCH (164 µL, 60 mU/mL) was added to the mixture and the reactions were incubated at 35 °C, 900 rpm for 80 min, in 1 mL reaction volume. Samples were taken at approximately 15-minute intervals and were quenched by the addition of acetonitrile (1:1 v/v). The precipitated protein was removed by centrifugation (10 min, 14,000 rpm) and the clear supernatant was injected directly to HPLC.

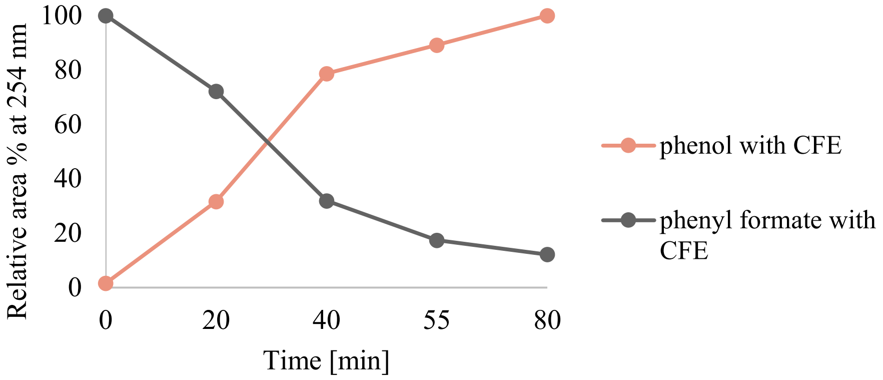

Figure S8. Hydrolysis of phenyl formate in KPi buffer (100 mM, pH 7.5). Reaction was performed in 1.5 mL polypropylene tubes at 35 °C, 900 rpm for 80 min, in 1 mL reaction volume, using 50 mM **2a** concentration, 60 mU/mL of CFE of *Pp*ATaseCH and KPi buffer (100 mM, pH 7.5) as reaction medium.

General procedure 4: Deformylation of 3a

The product, 2,4-dihydroxy-benzaldehyde [250 µL of 40 mM stock solution in KPi buffer (100 mM, pH 7.5), 10 mM final concentration] was mixed with KPi buffer (100 mM pH 7.5, volume adapted to total volume of reaction 1 mL). To simulate the same conditions, **2a** (5.5 µL, 50 mM final concentration) was added to the mixture and the reverse reaction (total volume 1 mL) was started by addition of the CFE of recombinant acyltransferase (60 or 100 mU/mL). The reaction mixture was shaken for 18 h at 35 °C and 750 rpm in an orbital shaker. Samples (100 µL) were taken after 18 h and were quenched by the addition

of acetonitrile (1:1 v/v). The precipitated protein was removed by centrifugation (10 min, 14,000 rpm) and the clear supernatant was injected to HPLC for conversion determination. All reactions were performed in duplicate and as a negative control, reactions without CFE were performed.

Table S11. Conversion values of the reverse reaction. Reactions were performed in 1.5 mL polypropylene tubes at 35 °C, 900 rpm for 18 h, in 1 mL reaction volume, using 10 mM of **3a**, 50 mM of **2a**, 60 mU or 100 mU CFE of acyltransferase and KPi buffer (100 mM, pH 7.5).

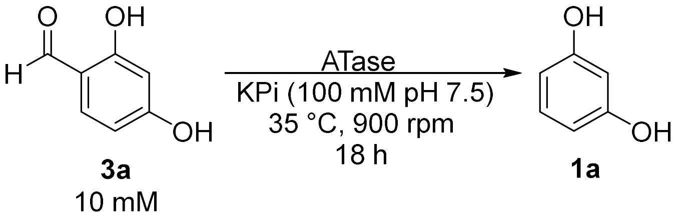

Acyltransferase	Enzyme quantity [mU/mL]	Conv. [%]
Without enzyme	-	n.d.
*Pp*ATaseCH	60	0.5
	100	1.0
	100	1.8*
*Cs*ATase	60	1.8
	100	2.7

n.d.= not detected
* without **2a**

General procedure 5: Bioformylation of 1a using different enzyme loadings
Resorcinol [250 µL of 40 mM stock solution dissolved in KPi buffer (100 mM, pH 7.5), 10 mM final concentration] was mixed with KPi buffer (100 mM, pH 7.5, volume adapted to total volume of reaction 1 mL). Phenyl formate (5.5 µL, 50 mM final concentration) was added to the mixture and the bioformylation (total volume 1 mL) was started by addition of the CFE of *Pp*ATaseCH or *Cs*ATase (20-100 mU/mL). The reaction mixture was shaken for 1 h at 35 °C and 900 rpm in an orbital shaker. Samples (100 µL) were taken after 1 h and were quenched by the addition of acetonitrile (1:1 v/v). The precipitated protein was removed by centrifugation (10 min, 14,000 rpm) and the clear supernatant was injected to HPLC for determination of conversion. All reactions were performed in duplicate and as a negative control, reactions without CFE were performed.

General procedure 6: Bioformylation of 1a using different concentration of 2a
Resorcinol [250 µL of 40 mM stock solution dissolved in KPi buffer (100 mM, pH 7.5), 10 mM final concentration] was mixed with KPi buffer (100 mM, pH 7.5, volume adapted to total volume of reaction 1 mL). Phenyl formate (30-100 mM final concentration, volume adapted to total volume of reaction 1 mL) was added to the mixture and the bioformylation (total volume 1 mL) was started by addition of the CFE of *Pp*ATaseCH (164 µL, 60 mU/mL) or the CFE of *Cs*ATase (83 µL). The reaction mixture was shaken for 1 h at 35 °C and 900 rpm in an orbital shaker. Samples (100 µL) were taken after 1 h and were quenched by the addition of acetonitrile (1:1 v/v). The precipitated protein was removed by centrifugation (10 min, 14,000 rpm) and the clear supernatant was injected to HPLC for determination of conversion. All reactions were performed in duplicate and as a negative control, reactions without CFE were performed.

General Procedure 7: Bioformylation of 1j monitored in time
Resorcinol derivative [200 µL of 10 mM stock solution in KPi buffer (100 mM, pH 7.5), 2 mM final concentration] was mixed with KPi buffer (100 mM pH 7.5, volume adapted to total volume of reaction 1 mL) with DMSO (100 µL, 10% v/v) to aid solubility. Phenyl formate (7.7 µL, 70 mM final concentration) was added to the mixture and the bioformylation (total volume 1 mL) was started by addition of the CFE of *Cs*ATase (83 µL, 60 mU/mL). The reaction mixture was shaken for 120 min at

35 °C and 900 rpm in an orbital shaker. Samples (10 µL) were taken every minute for the first 10 minutes, and then at 20, 30, 60, and 120 minutes. The samples were quenched by the addition of acetonitrile (1:1 v/v). The precipitated protein was removed by centrifugation (10 min, 14,000 rpm) and the clear supernatant was injected to HPLC for conversion determination. All reactions were performed in duplicate.

General Procedure 8: Bioformylation of different formyl acceptors
Resorcinol derivative [250 µL of 40 mM stock solution in KPi buffer (100 mM, pH 7.5), 10 mM final concentration] was mixed with KPi buffer (100 mM pH 7.5, volume adapted to total volume of reaction 1 mL) with DMSO (100 µL, 10% v/v) to aid solubility, except in the case of **1a**, where no DMSO was used. Phenyl formate (7.7 µL, 70 mM final concentration) was added to the mixture and the bioformylation (total volume 1 mL) was started by addition of the CFE of *Cs*ATase (83 µL, 60 mU/mL). The reaction mixture was shaken for 18 h at 35 °C and 900 rpm in an orbital shaker. Samples (100 µL) were taken after 18 h and were quenched by the addition of acetonitrile (1:1 v/v). The precipitated protein was removed by centrifugation (10 min, 14,000 rpm) and the clear supernatant was injected to HPLC for conversion determination. All reactions were performed in duplicate and as a negative control, reactions without CFE were performed.

General Procedure 9: Semi-preparative scale bioformylation of resorcinol derivatives using phenyl formate
A 250 mL Erlenmeyer flask was charged with the appropriate resorcinol derivative (100 mg, 10 mM final concentration; **1j** was used at 2 mM) in KPi buffer (100 mM pH 7.5). DMSO (10% v/v) was included to aid solubility, except in the case of **1a**, where no DMSO was used. Phenyl formate was then added to a final concentration of 70 mM (for detailed amounts see below). The reaction was initiated by the addition of crude cell extract (CFE) of *Cs*ATase (60 mU/mL). The mixture was incubated at 35 °C and shaken at 250 rpm in an orbital shaker. Conversion was monitored by HPLC, and the reaction was stopped once no further change in product formation was observed.
Work-up: The shaking was stopped and the reaction mixture was directly extracted with ethyl acetate. The reaction mixture was distributed into falcons (~25 mL in one falcon) and ethyl acetate was added 3 times (20 mL, 10 mL, 5 mL). The falcons were shaken thoroughly and were centrifuged for 10 min at 4000 rpm. The organic fractions were pooled together and were dried over anhydrous Na_2SO_4 and the solvent was removed under reduced pressure. The crude products (**3a-3f, 3i-3j**) were purified by flash chromatography or by preparative-HPLC (**3g-3h**). Compounds were characterized by ^{1}H, ^{13}C NMR spectroscopy, HPLC-MS and HR-MS.

2,4-dihydroxybenzaldehyde (3a)

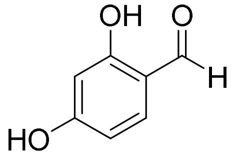

According to **General Procedure 9**, **1a** (100 mg, 0.9 mmol, 1.00 eq.), **2a** (639 µL, 6.3 mmol, 7 eq.) and *Cs*ATase CFE (7.2 mL, 60 mU/mL) were dissolved in KPi buffer (100 mM pH 7.5) with an overall resorcinol derivative concentration of 10 mM in the reaction flask. The flask was shaken at 35 °C for 1.5 hours. Upon completion, the reaction mixture was extracted, and the crude product was purified by flash chromatography (gradient: 0–60% ethyl acetate in cyclohexane), affording compound **3a** as a white solid in 74% yield (92 mg).
^{1}H NMR (300 MHz, DMSO) δ 10.90 (s, 1H), 10.62 (s, 1H), 9.92 (s, 1H), 7.53 (d, *J* = 8.6 Hz, 1H), 6.39 (dd, *J* = 8.6, 2.2 Hz, 1H), 6.32 (d, *J* = 2.2 Hz, 1H). ^{13}C NMR (75 MHz, DMSO) δ 191.3, 165.6, 163.6, 133.2, 115.6, 109.0, 102.6. HRMS: calcd for $C_7H_6O_3H^+$ ([M+H$^+$]) 139.0395, found 139.0395.

2,4-dihydroxy-5-methylbenzaldehyde (3b)

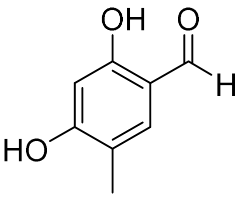

According to **General Procedure 9**, **1b** (100 mg, 0.8 mmol, 1.00 eq.), **2a** (614 µL, 5.6 mmol, 7 eq.) and *Cs*ATase CFE (6.4 mL, 60 mU/mL) were dissolved in KPi buffer (100 mM pH 7.5) containing DMSO (6.5 mL, 10%) with an overall resorcinol derivative concentration of 10 mM in the reaction flask. The flask was shaken at 35 °C for 2 hours. Upon completion, the reaction mixture was extracted, and the crude product was purified by column chromatography (cyclohexane:ethyl acetate, 8:2), affording compound **3b** as a yellow solid in 29% yield (35 mg).
^{1}H NMR (300 MHz, DMSO) δ 10.69 (s, 3H), 9.89 (s, 1H), 7.37 (d, *J* = 1.0 Hz, 1H), 6.38 (s, 1H), 2.04 (s, 2H). ^{13}C NMR (75 MHz, DMSO) δ 191.1, 163.8, 161.9, 132.6, 117.3, 115.2, 102.1, 15.4. HRMS: calcd for $C_8H_8O_3H^+$ ([M+H$^+$]) 153.0551, found 153.0544.

2,4-dihydroxy-6-methylbenzaldehyde (3c)

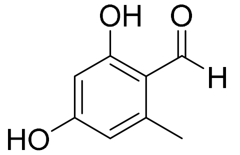

According to **General Procedure 9**, **1c** (100 mg, 0.8 mmol, 1.00 eq.), **2a** (614 µL, 5.6 mmol, 7 eq.) and *Cs*ATase CFE (6.4 mL, 60 mU/mL) were dissolved in KPi buffer (100 mM pH 7.5) containing DMSO (6.5 mL, 10%) with an overall resorcinol derivative concentration of 10 mM in the reaction flask. The flask was shaken at 35 °C for 4 hours. Upon completion, the reaction mixture was extracted, and the crude product was purified by flash chromatography (gradient: 0–60% ethyl acetate in cyclohexane), affording compound **3c** as a light yellow solid in 72% yield (88 mg).
^{1}H NMR (300 MHz, DMSO) δ 12.05 (s, 1H), 10.68 (s, 1H), 10.05 (s, 1H), 6.20 (dd, *J* = 2.3, 1.0 Hz, 1H), 6.12 (d, *J* = 2.2 Hz, 1H), 2.45 (s, 3H). ^{13}C NMR (75 MHz, DMSO) δ 193.4, 165.8, 165.6, 145.2, 113.1, 111.2, 100.6, 19.0. HRMS: calcd for $C_8H_8O_3H^+$ ([M+H$^+$]) 153.0551, found 153.0545.

2,4-dihydroxy-6-methoxybenzaldehyde (3d)

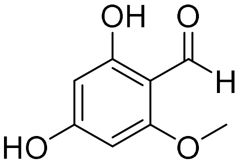

According to **General Procedure 9**, **1d** (100 mg, 0.7 mmol, 1.00 eq.), **2a** (545 µL, 5 mmol, 7 eq.) and *Cs*ATase CFE (5.7 mL, 60 mU/mL) were dissolved in KPi buffer (100 mM pH 7.5) containing DMSO (5.9 mL, 10%) with an overall resorcinol derivative concentration of 10 mM in the reaction flask. The flask was shaken at 35 °C for 4 hours. Upon completion, the reaction mixture was extracted, and the crude product was purified by flash chromatography (gradient: 0–60% ethyl acetate in cyclohexane), affording compound **3d** as a white solid in 19% yield (23 mg).
^{1}H NMR (300 MHz, DMSO) δ = 12.35 (s, 1H), 10.96 (s, 1H), 9.94 (s, 1H), 5.98 (d, *J*=2.0, 1H), 5.86 (d, *J*=2.0, 1H), 3.82 (s, 3H). ^{13}C NMR (75 MHz, DMSO) δ = 191.2, 167.9, 165.6, 164.4, 105.0, 95.4, 91.7, 56.4. HRMS: calcd for $C_8H_8O_4H^+$ ([M+H$^+$]) 169.0500, found 169.0494.

5-chloro-2,4-dihydroxybenzaldehyde (3e)

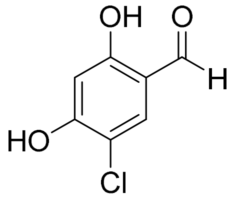

According to **General Procedure 9**, **1e** (100 mg, 0.7 mmol, 1.00 eq.), **2a** (539 µL, 4.9 mmol, 7 eq.) and *Cs*ATase CFE (5.5 mL, 60 mU/mL) were dissolved in KPi buffer (100 mM pH 7.5) containing DMSO (5.7 mL, 10%) with an overall resorcinol derivative concentration of 10 mM in the reaction flask. The flask was shaken at 35 °C for 2 hours. Upon completion, the reaction mixture was extracted, and the crude product was purified by flash chromatography (gradient: 0–60% ethyl acetate in cyclohexane), affording compound **3e** as a white solid in 42% yield (59 mg).
^{1}H NMR (300 MHz, DMSO) δ 10.90 (s, 1H), 10.62 (s, 1H), 9.92 (s, 1H), 7.53 (d, *J* = 8.6 Hz, 1H), 6.39 (dd, *J* = 8.6, 2.2 Hz, 1H), 6.32 (d, *J* = 2.2 Hz, 1H). ^{13}C NMR (75 MHz, DMSO) δ 191.3, 165.6, 163.6, 133.2, 115.6, 109.0, 102.6. HRMS: calcd for $C_7H_5ClO_3H^+$ ([M+H$^+$]) 173.0005, found 172.9998.

2,4-dihydroxy-5-ethylbenzaldehyde (3f)

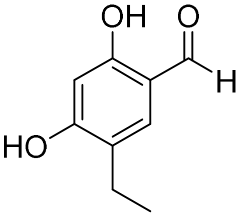

According to **General Procedure 9**, **1f** (100 mg, 0.7 mmol, 1.00 eq.), **2a** (545 µL, 5 mmol, 7 eq.) and *Cs*ATase CFE (5.7 mL, 60 mU/mL) were dissolved in KPi buffer (100 mM pH 7.5) containing DMSO (6 mL, 10%) with an overall resorcinol derivative concentration of 10 mM in the reaction flask. The flask was shaken at 35 °C for 5 hours. Upon completion, the reaction mixture was extracted, and the crude product was purified by column chromatography (cyclohexane:ethyl acetate, 8:2), affording compound **3f** as a brown solid in 12% yield (14.5 mg).

^{1}H NMR (300 MHz, DMSO) δ 10.72 (s, 1H), 10.62 (s, 1H), 9.91 (s, 1H), 7.39 (s, 1H), 6.38 (s, 1H), 2.45 (t, J = 7.5 Hz, 2H), 1.11 (t, J = 7.5 Hz, 3H). ^{13}C NMR (75 MHz, DMSO) δ 191.2, 163.5, 161.8, 131.1, 123.4, 115.2, 102.2, 22.2, 14.3. HRMS: calcd for $C_9H_{10}O_3H^+$ ([M+H$^+$]) 167.0708, found 167.0701.

2,4-dihydroxy-5-propylbenzaldehyde (3g)

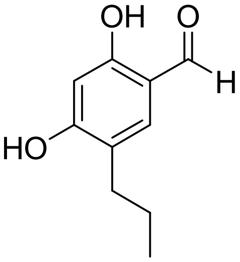

According to **General Procedure 9**, **1g** (100 mg, 0.65 mmol, 1.00 eq.), **2a** (501 µL, 4.6 mmol, 7 eq.) and *Cs*ATase CFE (5.2 mL, 60 mU/mL) were dissolved in KPi buffer (100 mM pH 7.5) containing DMSO (5.5 mL, 10%) with an overall resorcinol derivative concentration of 10 mM in the reaction flask. The flask was shaken at 35 °C for 4 hours. Upon completion, the reaction mixture was extracted, and the crude product was purified by preparative reversed-phase C18 silica gel chromatography [gradient: 30–90% acetonitrile (+0.1% formic acid) in water (+0.1% formic acid)], affording compound **3g** as a brown solid in 42% yield (49.7 mg).

^{1}H NMR (300 MHz, DMSO) δ = 10.71 (s, 1H), 10.60 (s, 1H), 9.90 (s, 1H), 7.36 (s, 1H), 6.38 (s, 1H), 2.41 (dd, J=8.5, 6.5, 2H), 1.51 (h, J=7.4, 2H), 0.87 (t, J=7.3, 3H). ^{13}C NMR (75 MHz, DMSO) δ = 191.2, 163.6, 161.8, 132.0, 121.8, 115.2, 102.3, 31.2, 22.7, 14.2. HRMS: calcd for $C_{10}H_{12}O_3H^+$ ([M+H$^+$]) 181.0864, found 181.0861.

2,4-dihydroxy-5-hexylbenzaldehyde (3h)

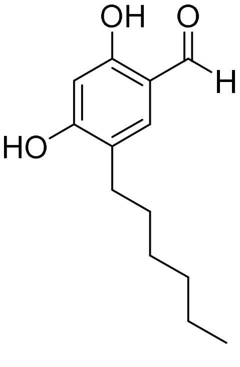

According to **General Procedure 9**, **1h** (100 mg, 0.5 mmol, 1.00 eq.), **2a** (0.404 µL, 3.6 mmol, 7 eq.) and *Cs*ATase CFE (5.9 mL, 60 mU/mL) were dissolved in KPi buffer (100 mM pH 7.5) containing DMSO (4.4 mL, 10%) with an overall resorcinol derivative concentration of 10 mM in the reaction flask. The flask was shaken at 35 °C for 6 hours. Upon completion, the reaction mixture was extracted, and the crude product was purified by preparative reversed-phase C18 silica gel chromatography [gradient: 30–90% acetonitrile (+0.1% formic acid) in water (+0.1% formic acid)], affording compound **3h** as a light yellow solid in 63% yield (72 mg).

^{1}H NMR (300 MHz, DMSO) δ = 10.70 (s, 2H), 9.89 (s, 1H), 7.35 (s, 1H), 6.37 (s, 1H), 2.42 (dd, J=8.7, 6.5, 3H), 1.35 – 1.20 (m, 8H), 0.90 – 0.79 (m, 3H). ^{13}C NMR (75 MHz, DMSO) δ = 191.3, 163.7, 161.9, 131.9, 122.1, 115.2, 102.3, 31.7, 29.6, 29.1, 28.9, 22.5, 14.4. HRMS: calcd for $C_{13}H_{18}O_3H^+$ ([M+H$^+$]) 223.1334, found 223.1320.

2,4-dihydroxy-1-naphthaldehyde (3i)

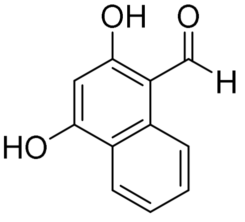

According to **General Procedure 9**, **1i** (100 mg, 0.62 mmol, 1.00 eq.), **2a** (0.476 mL, 4.4 mmol, 7 eq.) and *Cs*ATase CFE (4.7 mL, 60 mU/mL) were dissolved in KPi buffer (100 mM pH 7.5) containing DMSO (5.3 mL, 10%) with an overall resorcinol derivative concentration of 10 mM in the reaction flask. The flask was shaken at 35 °C for 3 hours. Upon completion, the reaction mixture was extracted, and the crude product was purified by flash chromatography (gradient: 0–60% ethyl acetate in cyclohexane), affording compound **3i** as a brown solid in 7% yield (8 mg).

^{1}H NMR (300 MHz, MeOD) δ 10.54 (s, 1H), 8.39 (d, *J* = 8.5 Hz, 1H), 8.19 (dd, *J* = 8.4, 1.5 Hz, 1H), 7.59 (ddd, *J* = 8.4, 6.9, 1.5 Hz, 1H), 7.37 (ddd, *J* = 8.2, 6.9, 1.1 Hz, 1H), 6.40 (s, 1H).^{13}C NMR (75 MHz, MeOD) δ 190.8, 168.0, 163.4, 134.5, 129.4, 123.1 (d, *J* = 1.2 Hz), 120.6, 118.7, 106.0, 98.6. HRMS: calcd for $C_{11}H_8O_3H^+$ ([M+H$^+$]) 189.0551, found 189.0550.

2,4,6-trihydroxyisophthalaldehyde (4j)

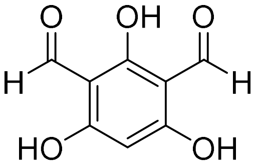

According to **General Procedure 9**, **1j** (100 mg, 0.8 mmol, 1.00 eq.), **2a** (3.1 mL, 5.5 mmol, 7 eq.) and *Cs*ATase CFE (30 mL, 60 mU/mL) were dissolved in KPi buffer (100 mM pH 7.5) containing DMSO (5.4 mL, 10%) with an overall resorcinol derivative concentration of 2 mM in the reaction flask. The flask was shaken at 35 °C for 3 hours. Upon completion, the reaction mixture was extracted, and the crude product was purified by flash chromatography (gradient: 0–60% ethyl acetate in cyclohexane), affording compound **4j** as a bordeaux red solid in 57% yield (82 mg).

^{1}H NMR (300 MHz, DMSO) δ = 12.51 (s, 2H), 10.00 (s, 2H), 5.89 (s, 1H). ^{13}C NMR (75 MHz, DMSO) δ = 191.9, 169.9, 169.5, 104.2, 94.5. HRMS: calcd for $C_8H_6O_5H^+$ ([M+H$^+$]) 183.0293, found 183.0285.

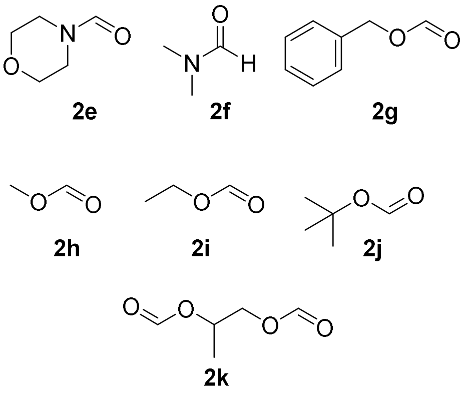

Figure S9. Not accepted formyl donors.

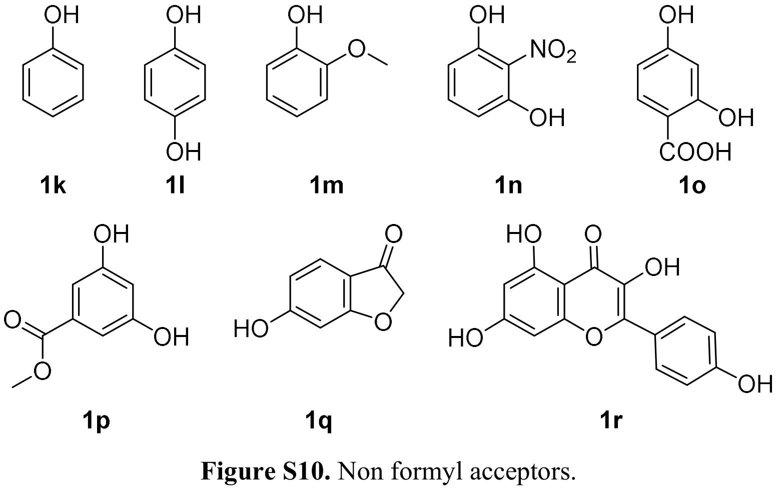

Figure S10. Non formyl acceptors.

4. Synthesis of 1,3-phenylene diformate and its hydrolysis in aqueous reaction conditions

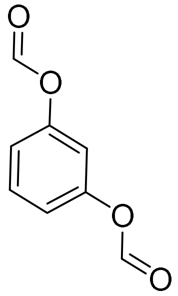

1,3-phenylene diformate was prepared according to a modified method described by Oestreich and co-workers.[2] Acetic anhydride (10 mL, 0.1 mol, 12.00 eq.) and concentrated formic acid (7 mL, 0.18 mol, 20.00 eq.) were placed in a 100 mL-round bottom flask and stirred in at 60 °C for 2 h. Resorcinol (1 g, 0.009 mol, 1.0 eq.) and solid $NaHCO_3$ (3.2 g, 0.36 mol, 4.0 eq.) were added portion wise at room temperature and the reaction mixture was stirred for another 20 h. Distilled H_2O (5 mL) and CH_2Cl_2 (10 mL) were added to the mixture and the phases were separated. The aqueous layer was extracted with CH_2Cl_2 (2 × 10 mL) and the combined organic layers were washed brine (1 × 10 mL). The organic layer was dried over $MgSO_4$, filtered and the solvent was evaporated under reduced pressure. The product was obtained as a colorless liquid and analyzed by NMR. ^{1}H NMR (300 MHz, $CDCl_3$) δ 8.32 (s, 2H), 7.46 (dd, J = 8.5, 7.9 Hz, 1H), 7.11 (dd, J = 8.2, 2.2 Hz, 2H), 7.05 (t, J = 2.2 Hz, 1H).^{13}C NMR (75 MHz, $CDCl_3$) δ 158.6, 150.3, 130.3, 119.2, 114.8.

The substrate, 1,3-phenylene diformate [1.2 µL, 10 mM final concentration] was mixed with KPi buffer (100 mM pH 7.5, volume adapted to total volume of reaction 1 mL). The reaction mixture was shaken for 18 h at 35 °C and 900 rpm in an orbital shaker. Samples (40 µL) were taken after 1 min, 10 min, 1 h, 3 h and 18 h and were injected to HPLC-MS.

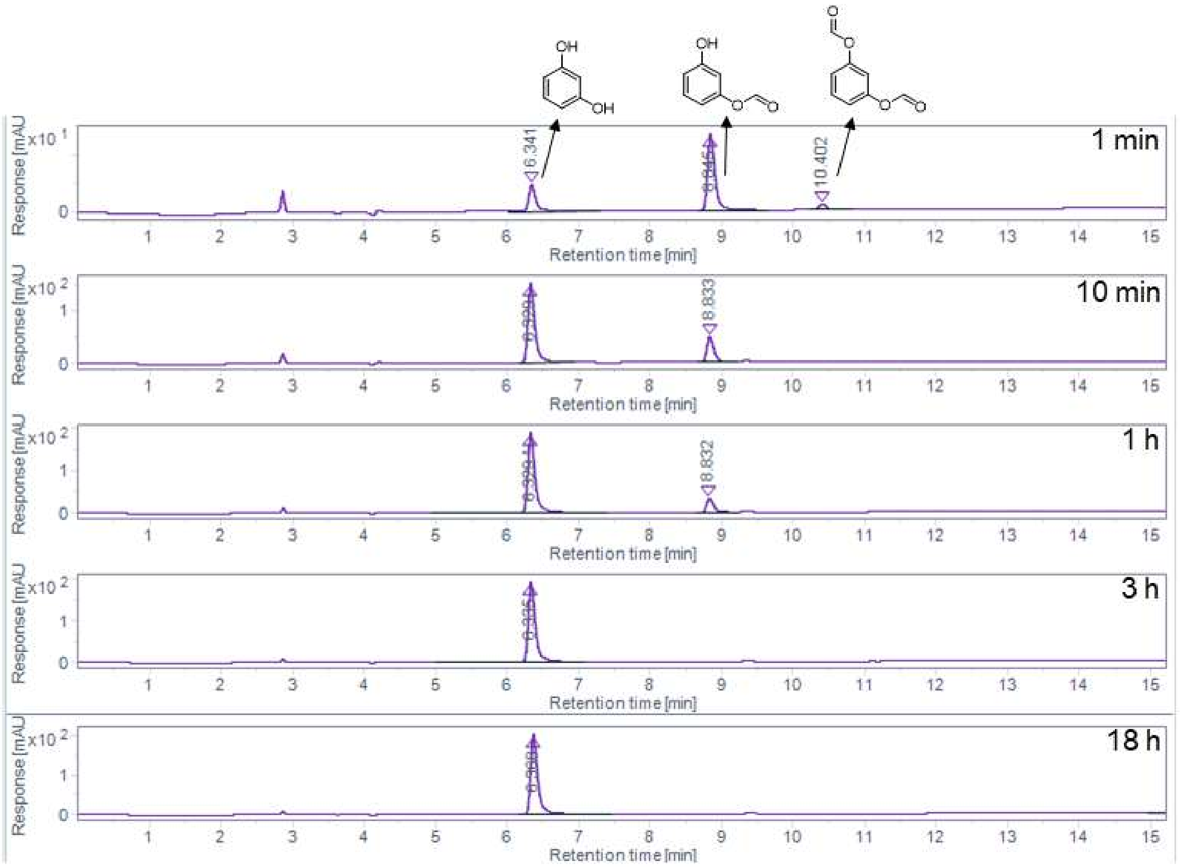

Figure S11. Hydrolysis of 1,3-phenylene diformate in KPi buffer (100 mM pH 7.5).

5. Spectroscopic data

Representative chromatograms and calibration curves

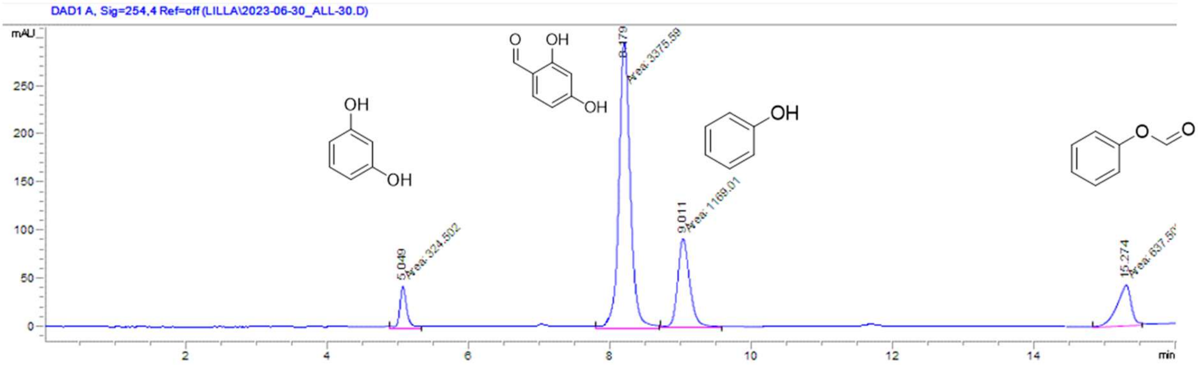

Figure S12. Representative HPLC-chromatogram showing the bioformylation of **1a** using **2a**

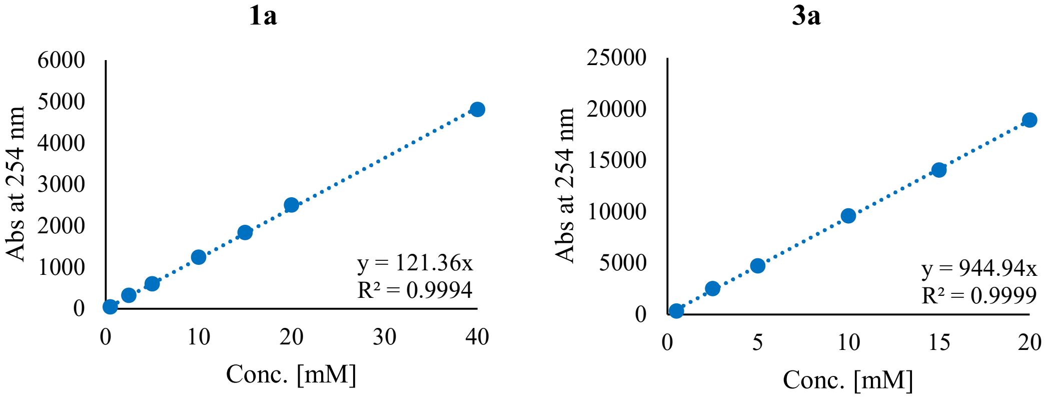

Figure S13. Calibration curve for **1a** and **3a**.

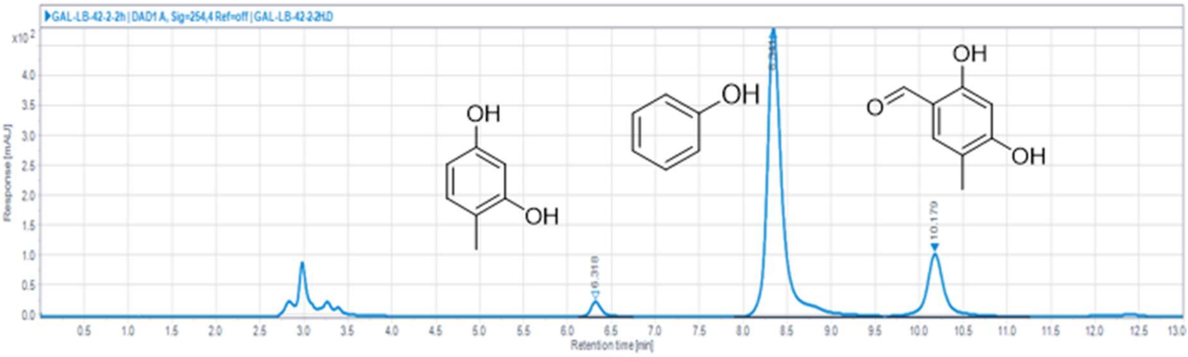

Figure S14. Representative HPLC-chromatogram showing the bioformylation of **1b** using **2a**

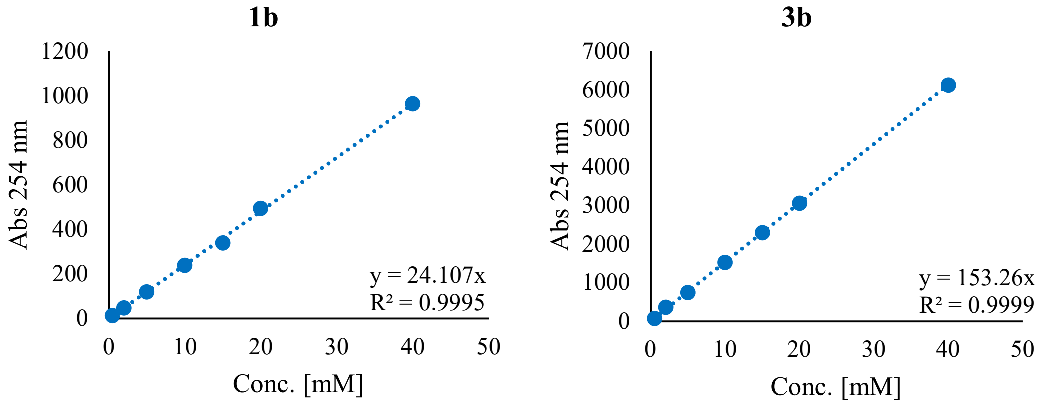

Figure S15. Calibration curve for **1b** and **3b**.

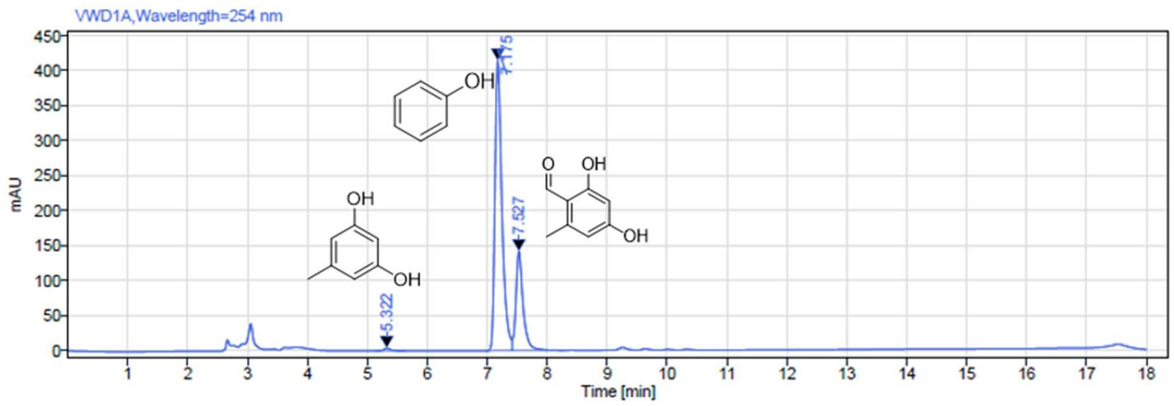

Figure S16. Representative HPLC-chromatogram showing the bioformylation of **1c** using **2a**.

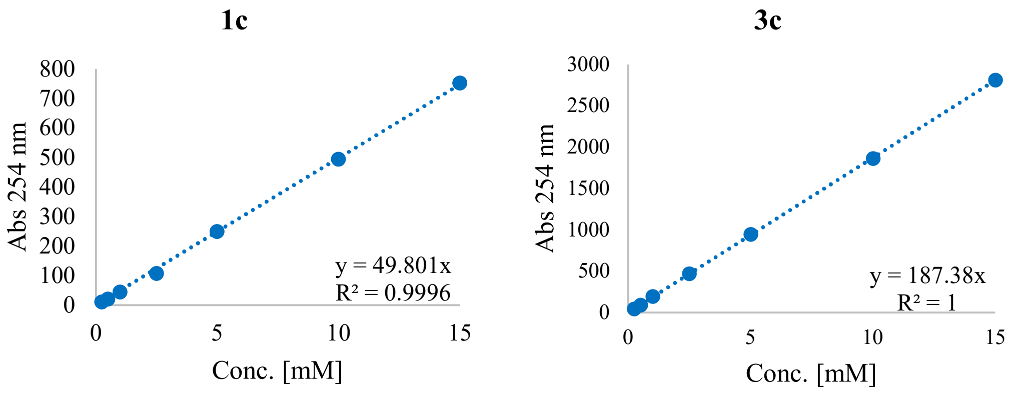

Figure S17. Calibration curve for **1c** and **3c**.

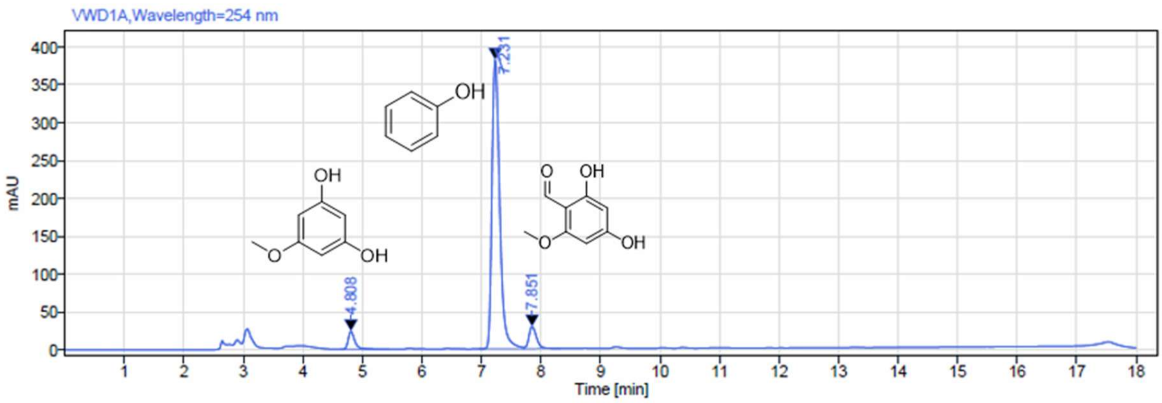

Figure S18. Representative HPLC-chromatogram showing the bioformylation of **1d** using **2a**.

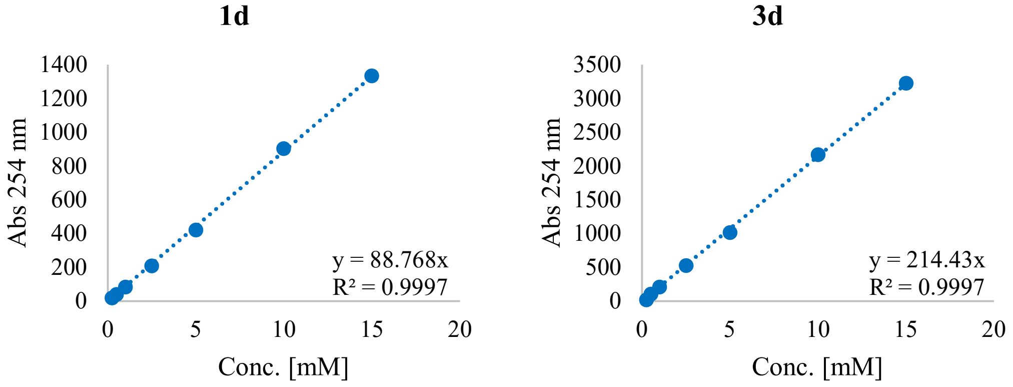

Figure S19. Calibration curve for **1d** and **3d**.

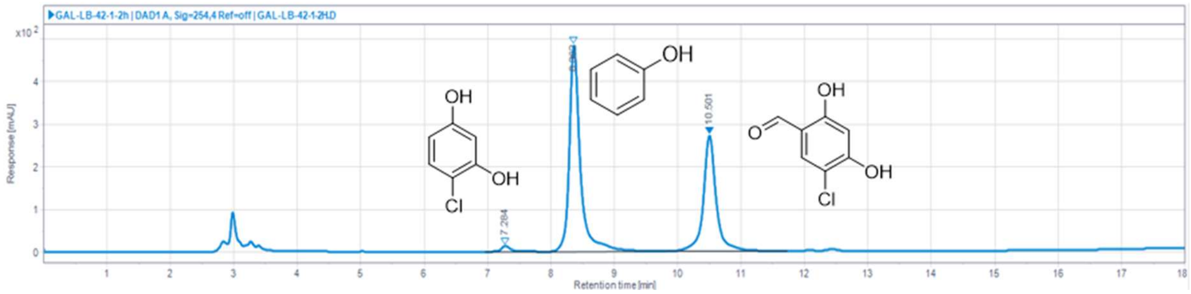

Figure S20. Representative HPLC-chromatogram showing the bioformylation of **1e** using **2a**.

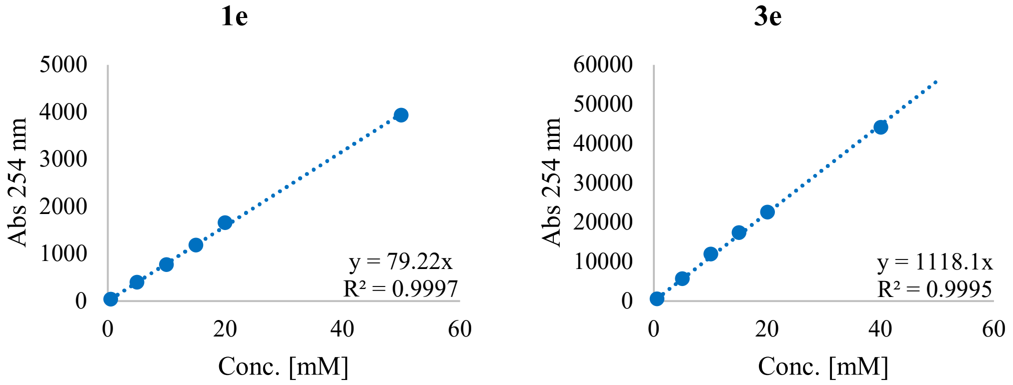

Figure S21. Calibration curve for **1e** and **3e**.

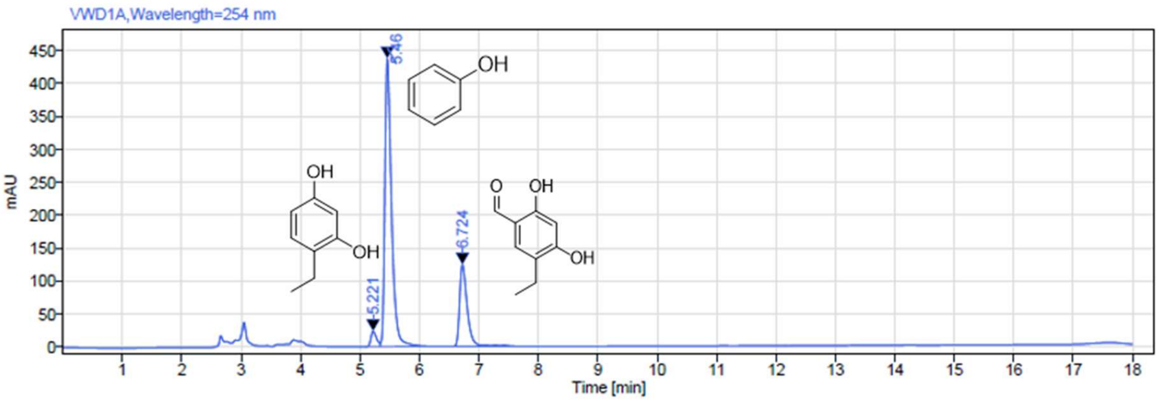

Figure S22. Representative HPLC-chromatogram showing the bioformylation of **1f** using **2a**.

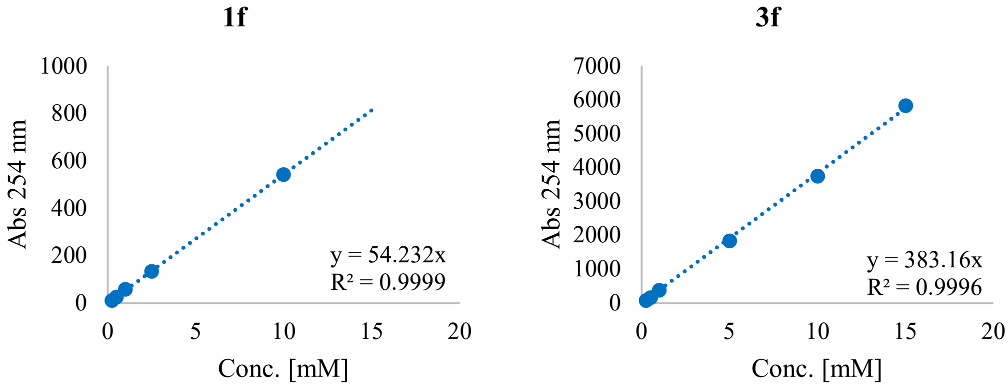

Figure S23. Calibration curve for **1f** and **3f**.

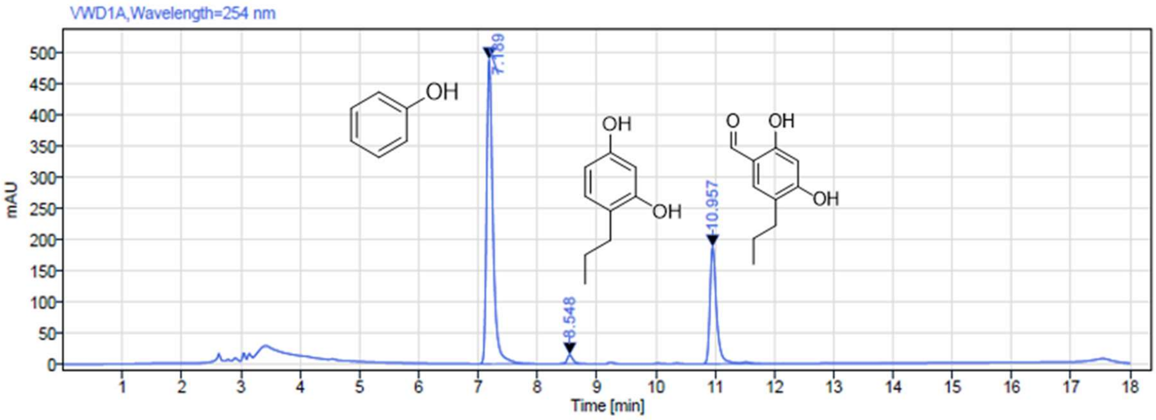

Figure S24. Representative HPLC-chromatogram showing the bioformylation of **1g** using **2a**.

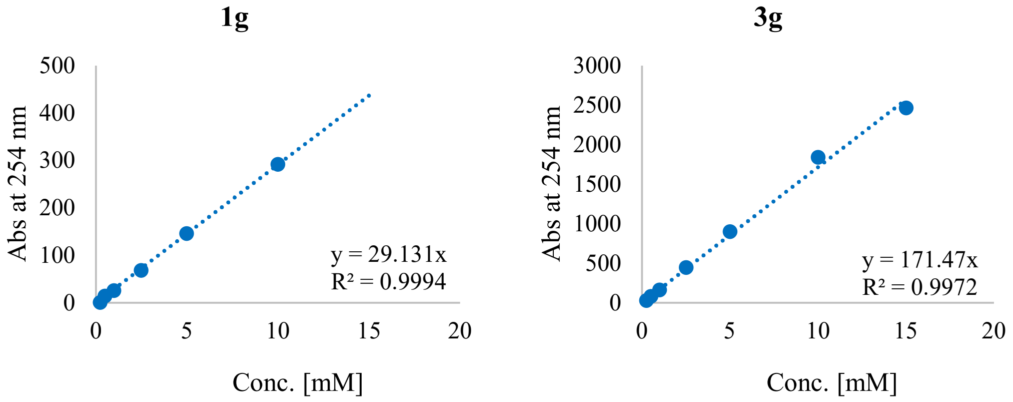

Figure S25. Calibration curve for **1g** and **3g**.

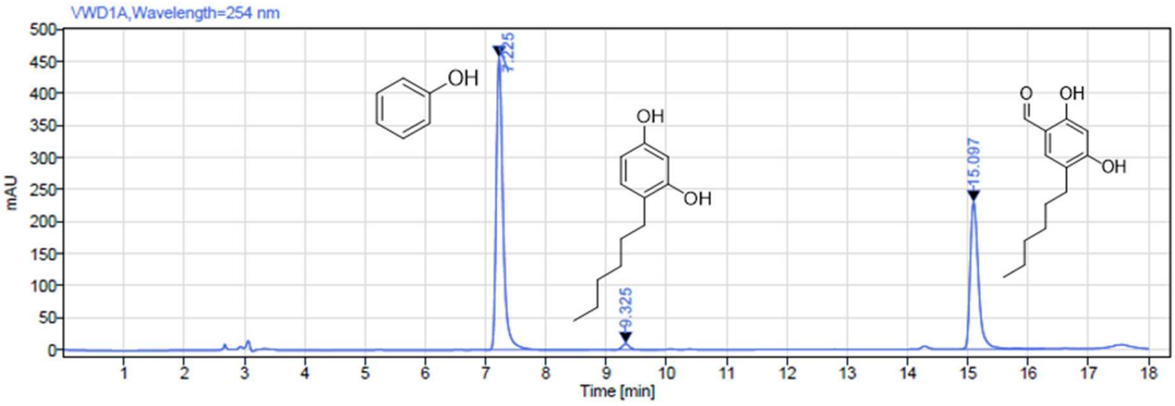

Figure S26. Representative HPLC-chromatogram showing the bioformylation of **1h** using **2a**.

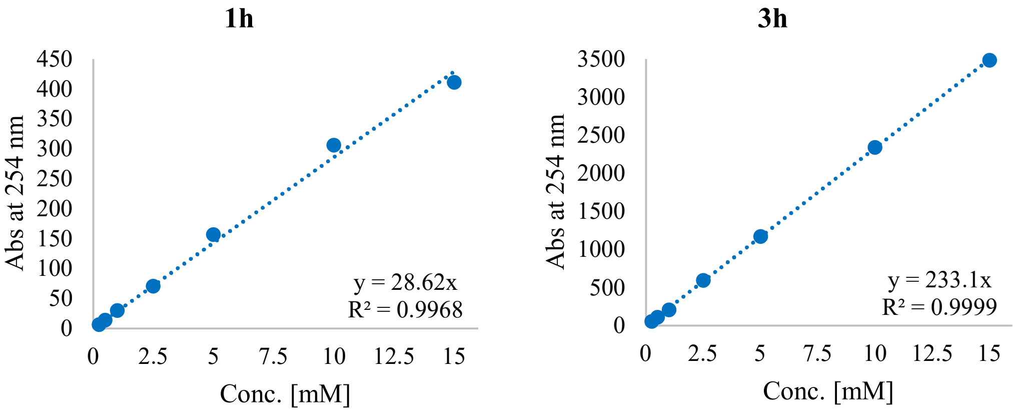

Figure S27. Calibration curve for **1h** and **3h**.

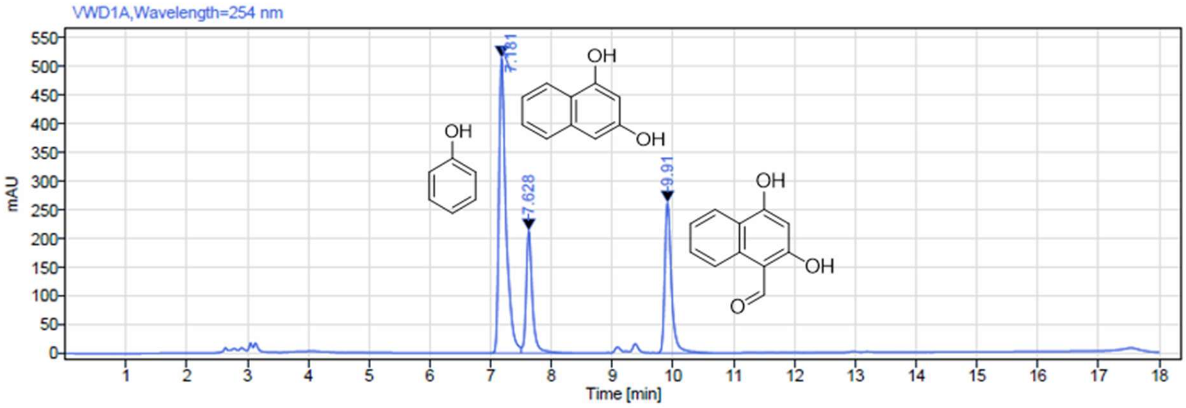

Figure S28. Representative HPLC-chromatogram showing the bioformylation of **1i** using **2a**.

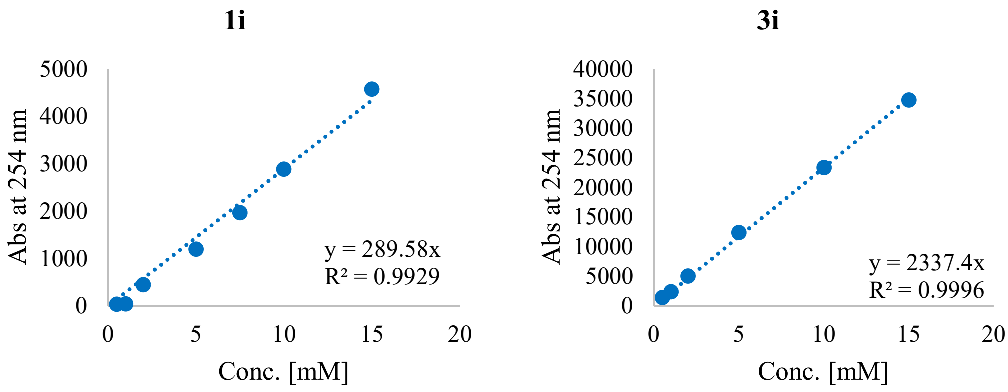

Figure S29. Calibration curve for **1i** and **3i**.

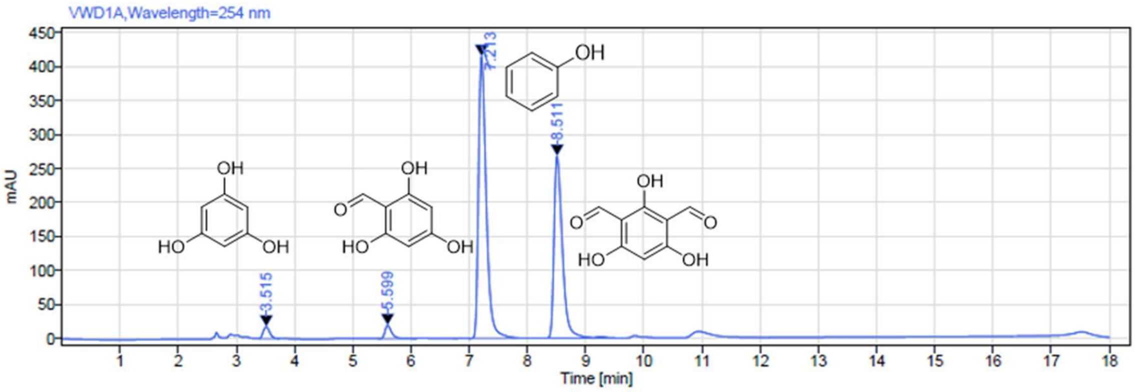

Figure S30. Representative HPLC-chromatogram showing the bioformylation of **1j** using **2a**.

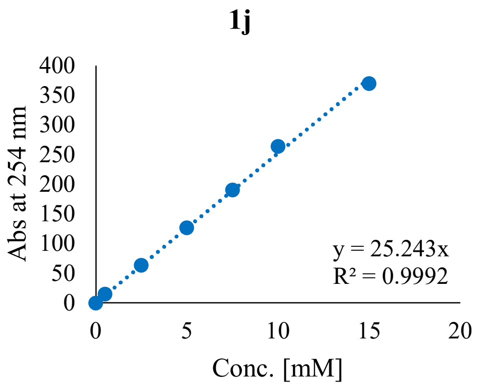

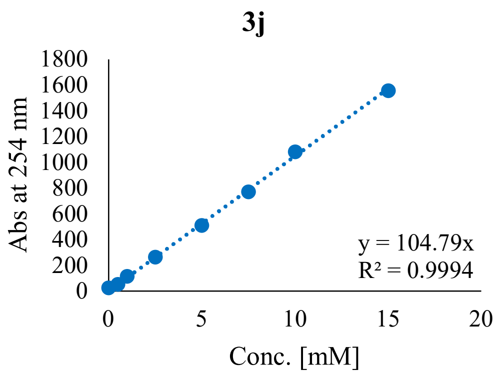

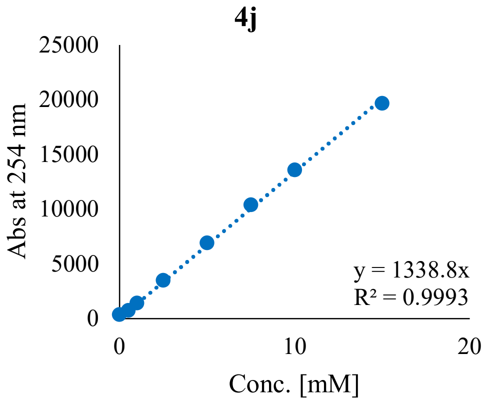

Figure S31. Calibration curve for **1j**, **3j** and **4j**.

6. X-ray crystallization and structure determination

Crystals of wild type *Cs*ATase and C88S *Cs*ATase (in 50 mM KPi, pH 7.5) were grown using the hanging-drop method. A 4 µL droplet (2 µL protein sample + 2 µL reservoir solution) was prepared and set against a reservoir solution of 500 µl containing PEG (400, 6-16%, w/v), PEG MME (350, 9-12%, v/v), in sodium acetate (0.05 M, pH 5.2). Crystals began to form after one day at 20 °C, though their size was insufficient for further analysis. Consequently, micro-seeding was employed, which resulted in slightly bigger crystals (Figure S32). The shape of the crystals was uniform; they resembled thin blades/needles. The crystals were soaked in a cryo-solution containing 15% (v/v) glycerol and the specific reservoir solution of the selected crystal at room temperature for 2–5 minutes before flash-cooling in liquid nitrogen.

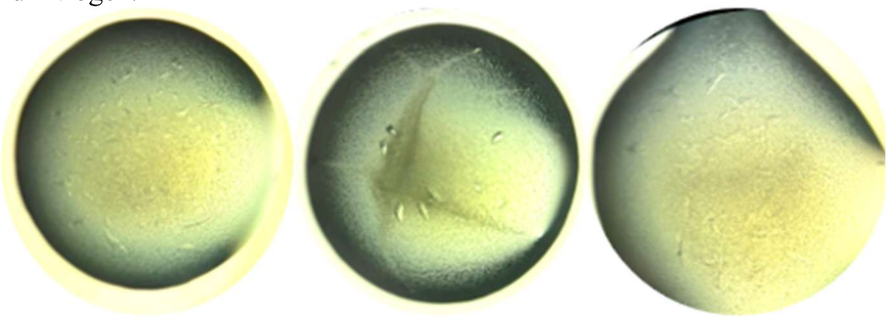

Figure S32. Crystals formed with condition Pi-PEG #C2 PEG (400, 6-16%, w/v), PEG MME (350, 9-12%, v/v), in sodium acetate (0.05 M, pH 5.2).

X-ray datasets of single crystals were collected under cryogenic temperature at the European Molecular Biology Laboratory (EMBL) beamline P14 at the Deutsches Elektronen-Synchrotron Hamburg (DESY) at a wavelength of 1 Å. The structure was determined by molecular replacement of the heterododecamer Alphafold[3] prediction. Image processing was performed using autoPROC (v1.1.7), molecular replacement and refinement were performed using the PHENIX[4] suite (v1.20.1).
Model building was performed using WinCoot[5] (v0.8.9.1). Iterative refinement cycles yielded geometries that very well corresponding with the observed electron densities. Structure validation was performed using MolProbity.[6] Crystallographic figures were prepared with Pymol.[7]

Table S12. Data collection and refinement statistics

	***Cs*ATase wild-type 9SKH**	***Cs*ATase PhlC C88S 9SKM**
**Data collection**		
Space group	P21	P21
Cell dimensions,		
a, b, c (Å)	94.470 185.447 120.680	93.733 184.359 120.782
α, β, γ (°)	90.000 103.213 90.000	90.000 103.656 90.000
Resolution (Å)	99.24-2.60 (2.79-2.60)	64.92-1.87 (1.90-1.87)
R_{meas}	0.35 (1.47)	0.15 (1.11)
I/σ/	4 (1.5)	11.2 (2.2)
Completeness (%)	spherical- 55.9 (14.3)<br>ellipsoidal- 88.5 (66.5)	98.1 (97.7)
Redundancy	7.1 (7.0)	6.5 (6.6)
**Refinement**		
Resolution (Å)	91.97-2.60	64.92-1.87
No. reflections	69156	318276
R_{work}/R_{free}	0.190/0.240	0.165/0.193
No. atoms		
Protein	53797	54054
Water	325	2704
Mean B-factors (in A^2)		
Protein	36.70	23.03
Water	27.90	29.16
R.M.S. deviation		
Bond lengths (Å)	0.003	0.008
Bond angles (°)	0.645	0.944

*Values in parentheses are for the highest-resolution shell.

7. Molecular docking

7.1. Protein structures and ligands

The models for *Ppi*ATase and *Pk*ATase were predicted using AlphaFold2 and have an ipTM score of 0.97 for both models, indicating high confidence in the overall structural accuracy.

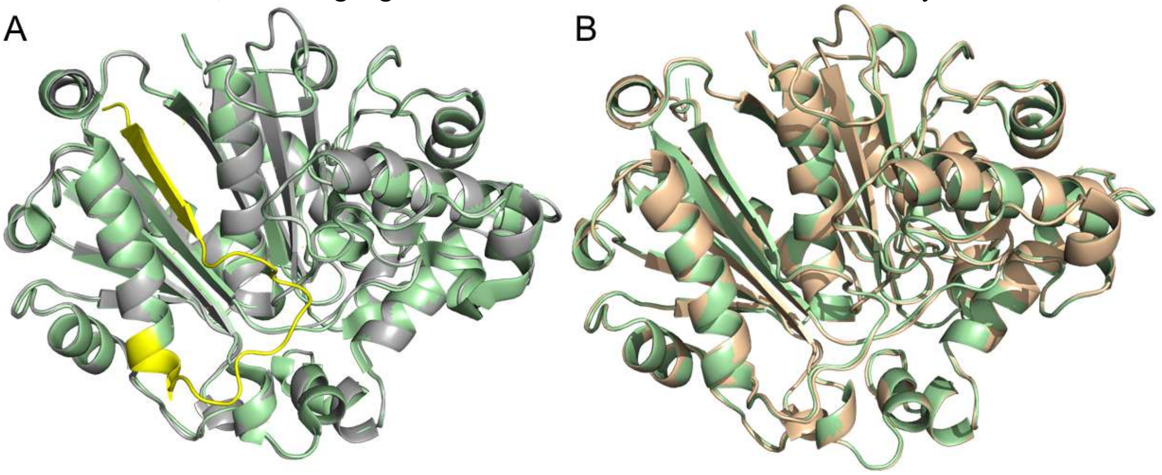

Figure S33. A) Structural overlay of the *PhlC* subunit of *Cs*ATase (PDB 9SKH) (palegreen) with the one from *Ppi*ATase (Alphafold model) (gray). Residues present in *Cs*ATase but absent in *Ppi*ATase are highlighted in yellow. B) Structural overlay of the *PhlC* subunit of *Cs*ATase (PDB 9SKH) (palegreen) with the one from *PhlC*-*Pk*ATase (Alphafold model) (wheat).

The crystal structure of wild-type *Cs*ATase, obtained in this study (PDB ID:9SKH), was used as the receptor for molecular docking. Phenyl formate and resorcinol were selected as the ligands for this in silico analysis. As the structure represents the apo-enzyme, the conformation of residue W211, known to act as a catalytic lid in the *phlC* subunit,[8] was manually adjusted to the closed state, based on the corresponding structure of *Pp*ATaseCH (PDB ID: 5MG5). Additionally, for the docking of resorcinol, the catalytic cysteine residue in the *phlC* subunit was modified to a formylated state, analogous to the acetylated cysteine observed in the same reference structure (PDB ID: 5MG5). After each structural modification, the model was energy-minimized using the Minimization MacroModel[9-10] module in Maestro (v14.2.118) from the Schrödinger Suite, employing the OPLS_2005 force field.

Molecular docking was carried out using both the Schrödinger Suite and AutoDock Vina (v1.2.7.) For the Schrödinger workflow, protein structures were prepared using the Protein Preparation Wizard,[11] and substrates were processed with LigPrep, using default parameters. Receptor grids were generated based on the residues C88, H56, H144, H347, Y124, and W211, with a grid size of 15 × 15 × 15 Å. Docking was then performed using the Ligand Docking (Glide)[12-15] protocol for each substrate.

For the AutoDock Vina workflow, the previously modified and minimized protein structure was prepared using AutoDock4 Tools. Docking was performed at the active site using a grid size of 14.6 × 14.6 × 14.6 Å. The docking parameters included an exhaustiveness value of 24, an energy range of 5, and 20 output modes, along with the default settings of AutoDock Vina. Notably, AutoDock Vina was used exclusively for the docking of resorcinol.

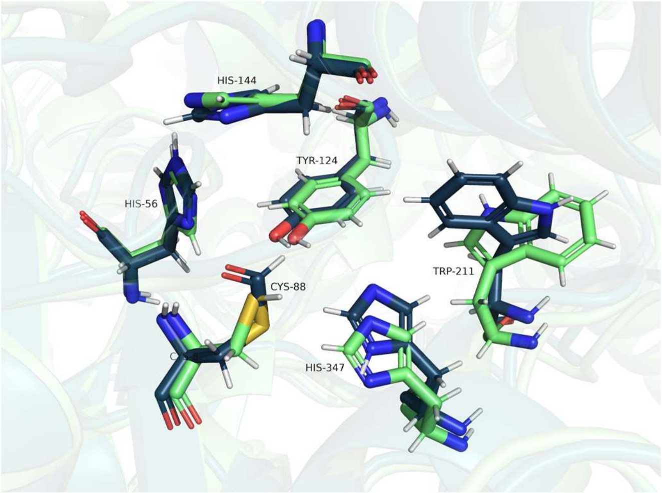

Figure S34. Comparison of the crystal structure of wild-type *Cs*ATase before (light green) and after modification and minimization (dark green).

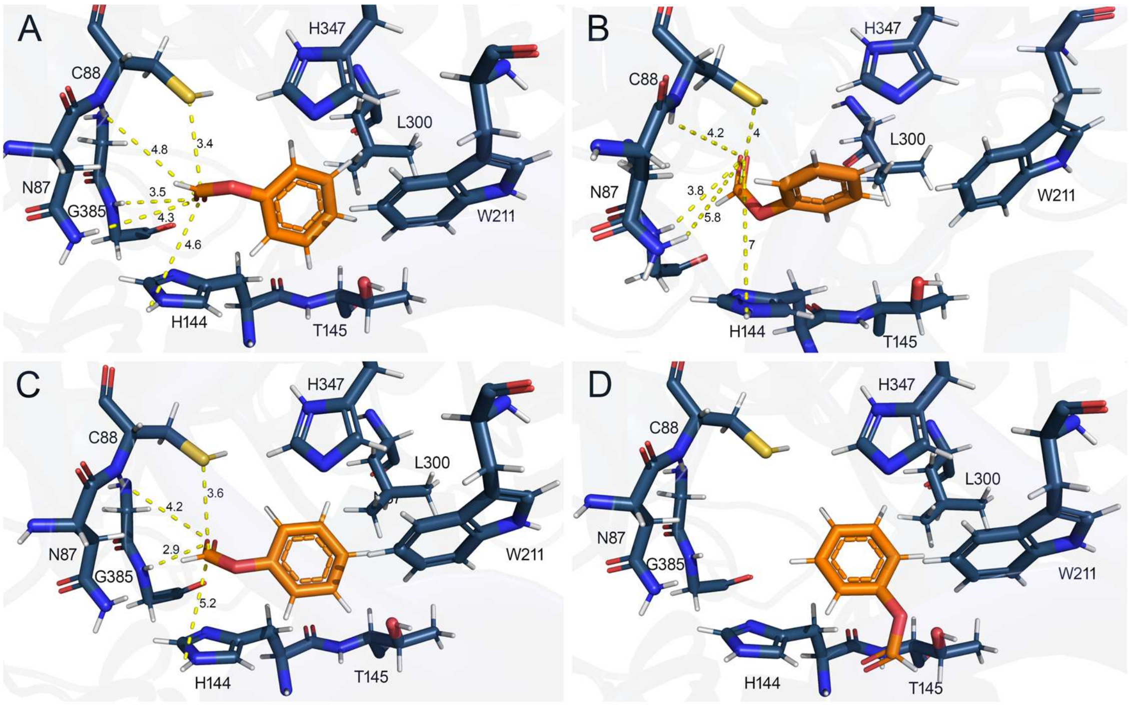

Figure S35. Non-productive docking poses obtained for phenyl formate with Schrödinger Glide.

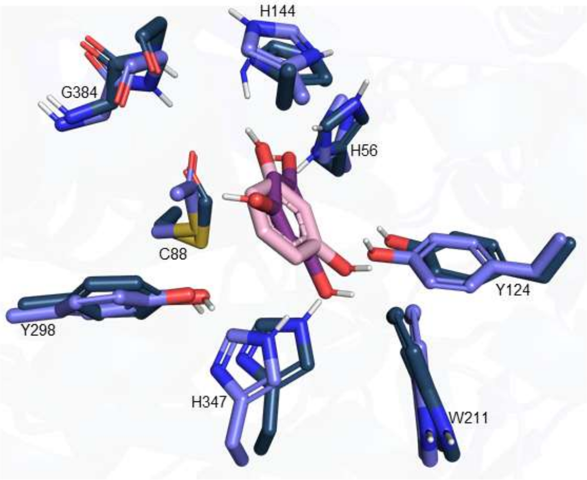

Figure S36. Crystal structure of *Cs*ATase (dark green) with resorcinol modeled into the active site and overlayed with the crystal structure of *Pp*ATaseCH (purple) soaked with phloroglucinol (PDB 5MG5).

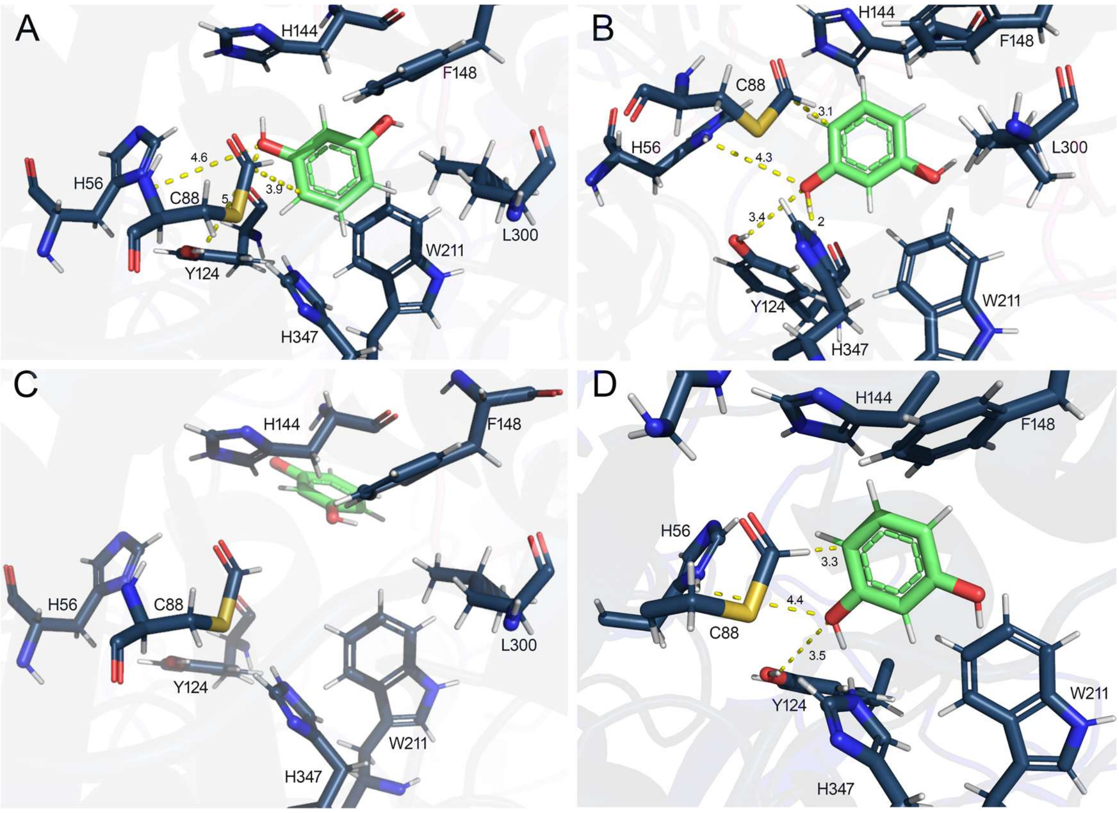

Figure S37. Non-productive docking poses obtained for resorcinol with Schrödinger Glide.

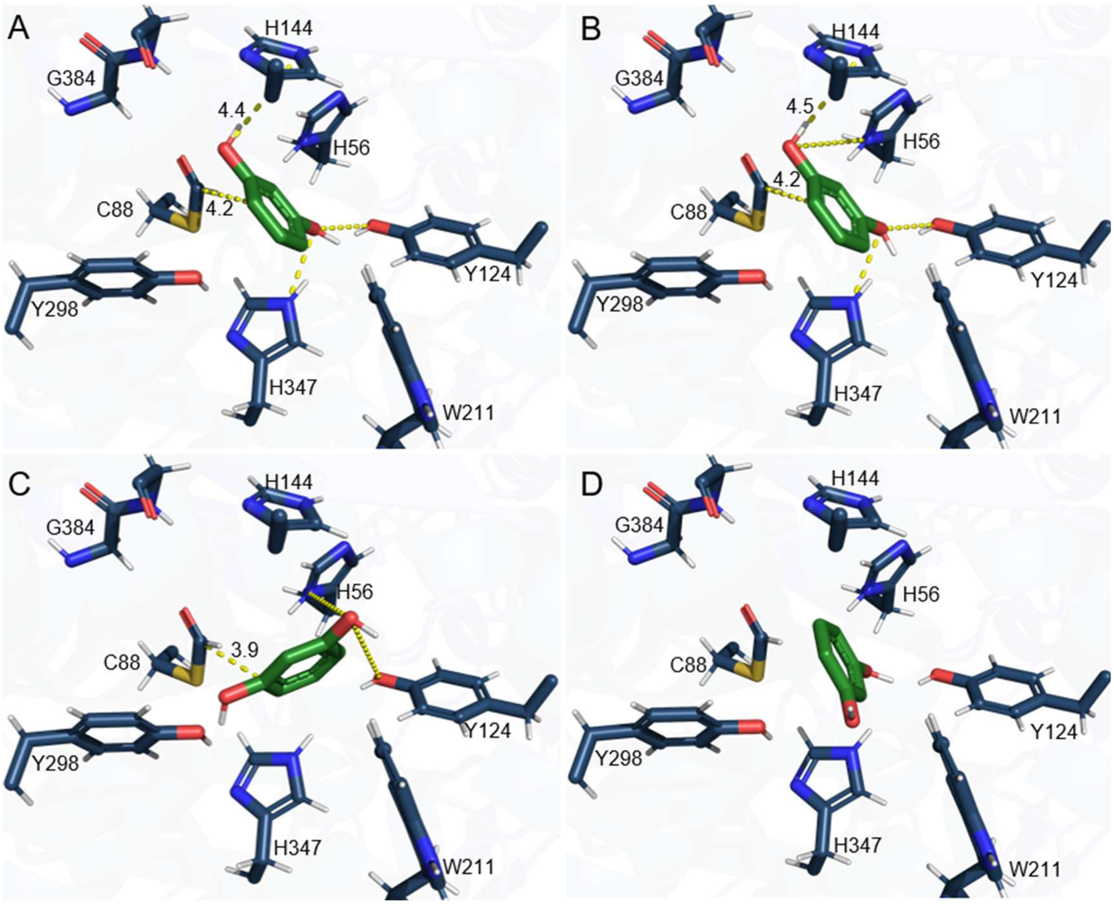

Figure S38. Non-productive docking poses obtained for resorcinol with Autodock Vina

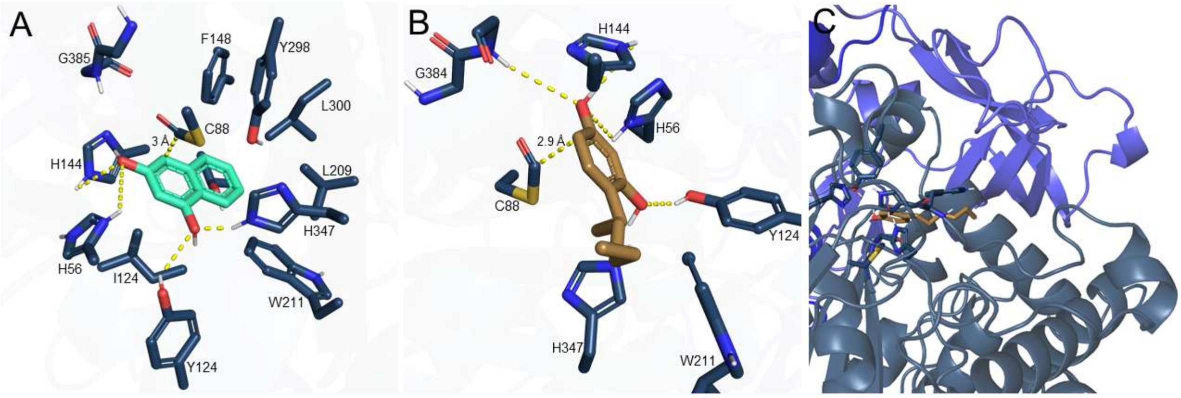

Figure S39. A) 1,3-dihydroxynaphthalene docked into the active site of *Cs*ATase. B) 4-hexyl-1,3-benzenediol docked into the active site of *Cs*ATase C) Hexyl-chain of 4-hexyl-1,3-benzenediol pointing out from the active site toward the solvent.

8. Additional figures

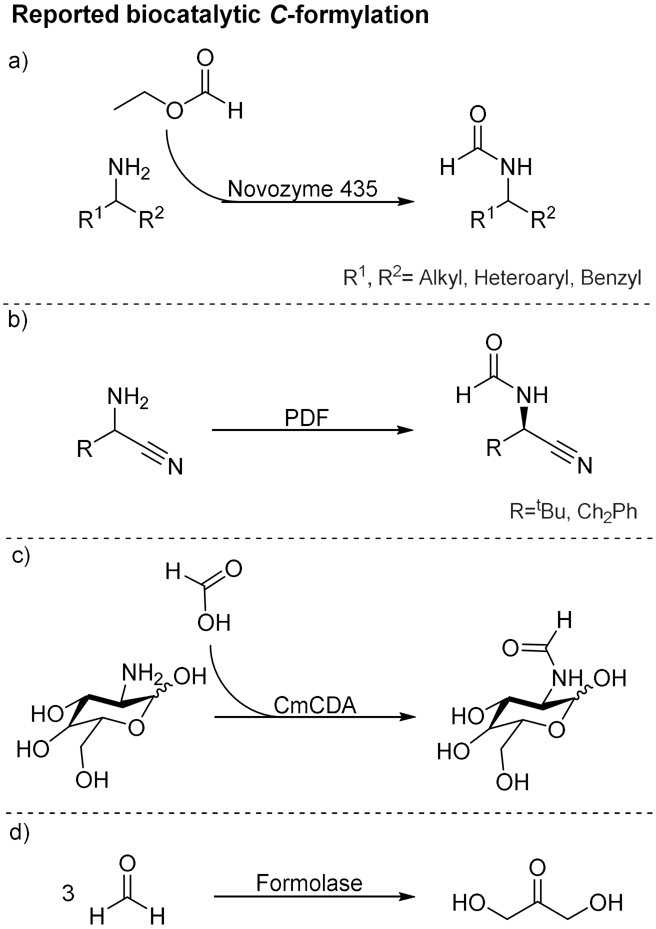

Figure S40. a) *N*-formylation of amines catalyzed by immobilized *Candida antarctica* lipase. b) PDF catalyzed formylation of α-aminonitriles. c) CmCDA-catalyzed *N*-formylation of glucosamine d) Artificial formolase-catalyzed carboligation of three molecules of formaldehyde.

9. NMR spectra

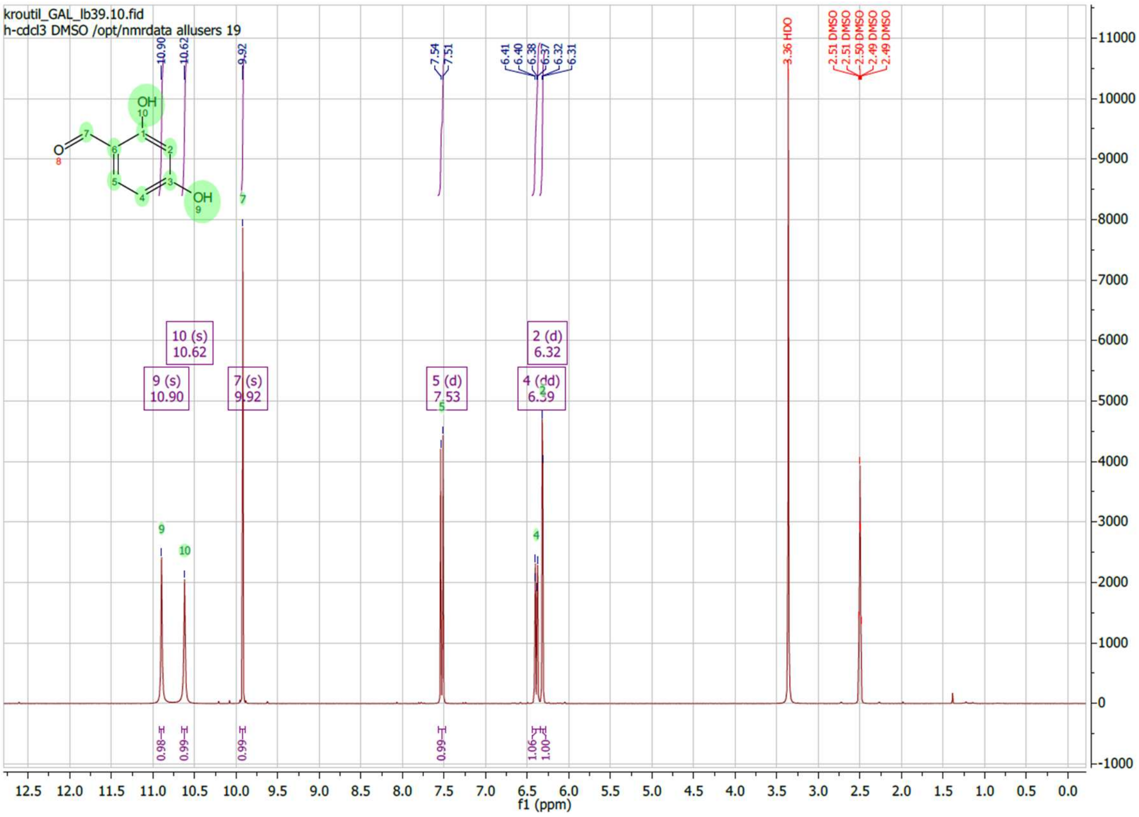

Figure S41. ^{1}H NMR spectrum of 2,4-dihydroxybenzaldehyde (**3a**) (solvent-DMSO-d_6, 300 MHz).

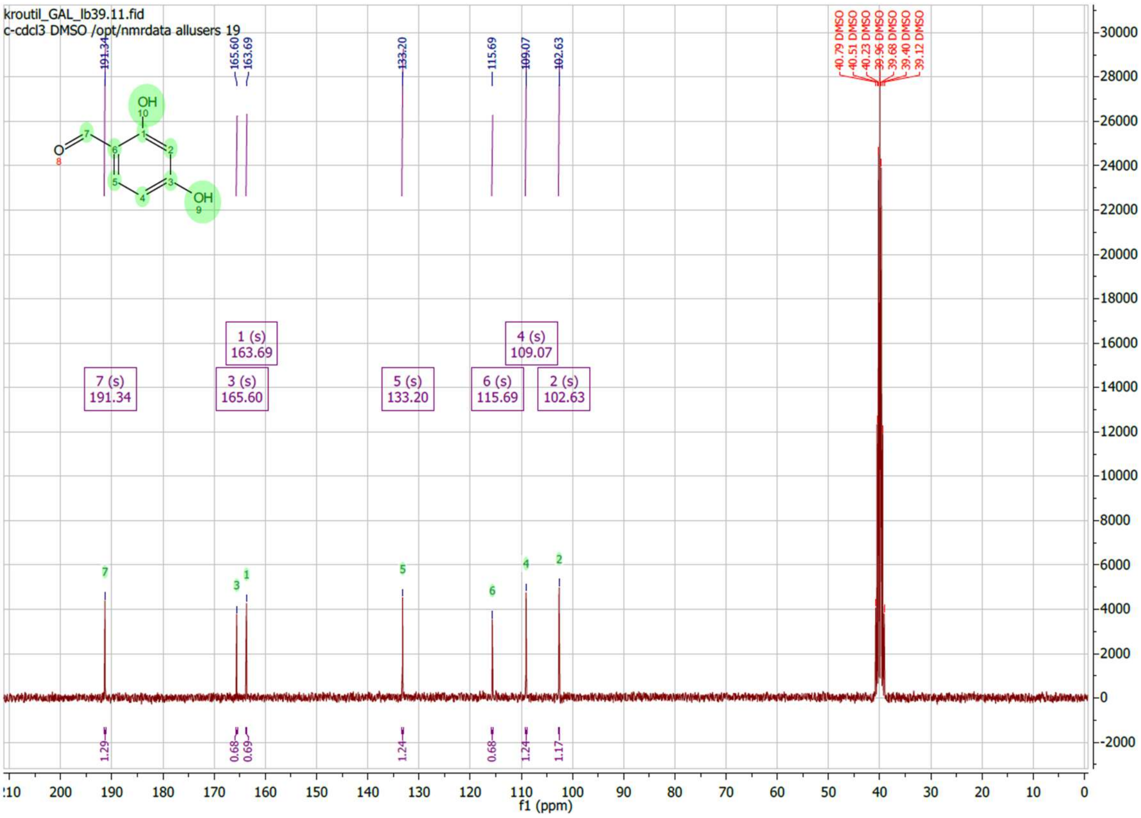

Figure S42. ^{13}C NMR spectrum of 2,4-dihydroxybenzaldehyde (**3a**) (solvent-DMSO-d_6, 75 MHz).

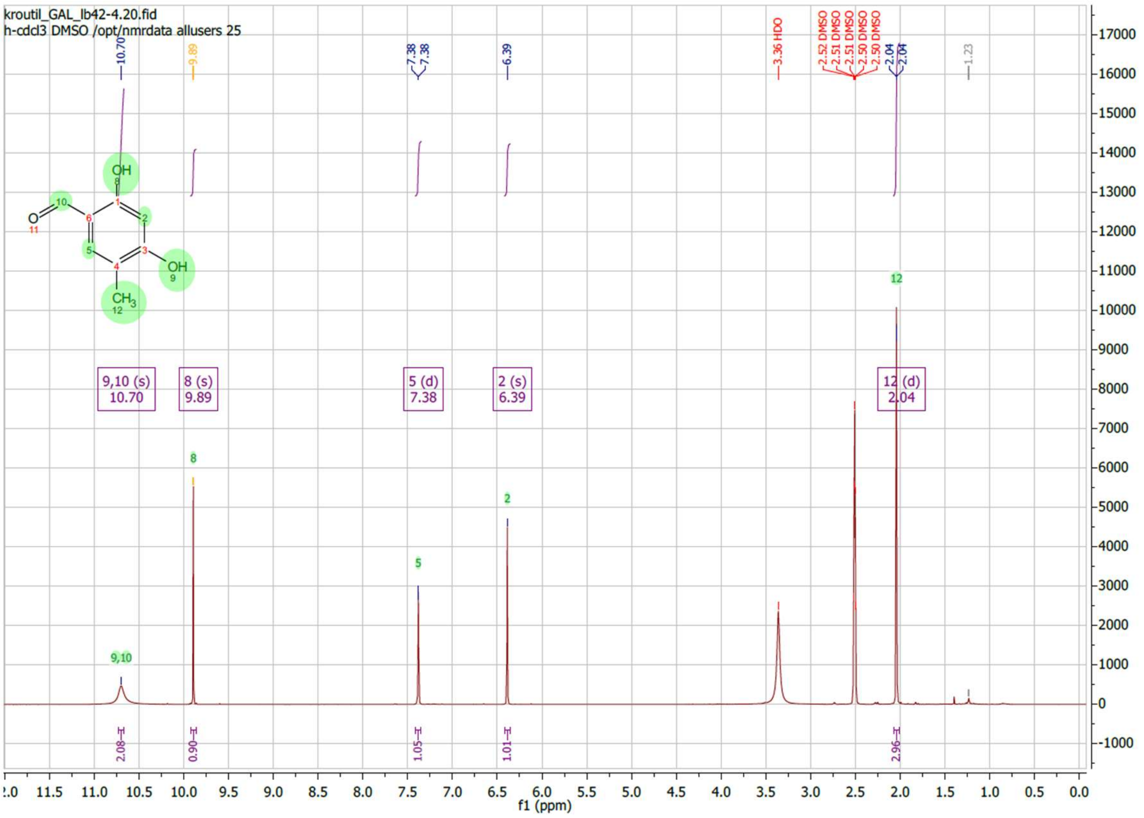

Figure S43. ^{1}H NMR spectrum of 2,4-dihydroxy-5-methylbenzaldehyde (**3b**) (solvent-DMSO-d₆, 300 MHz).

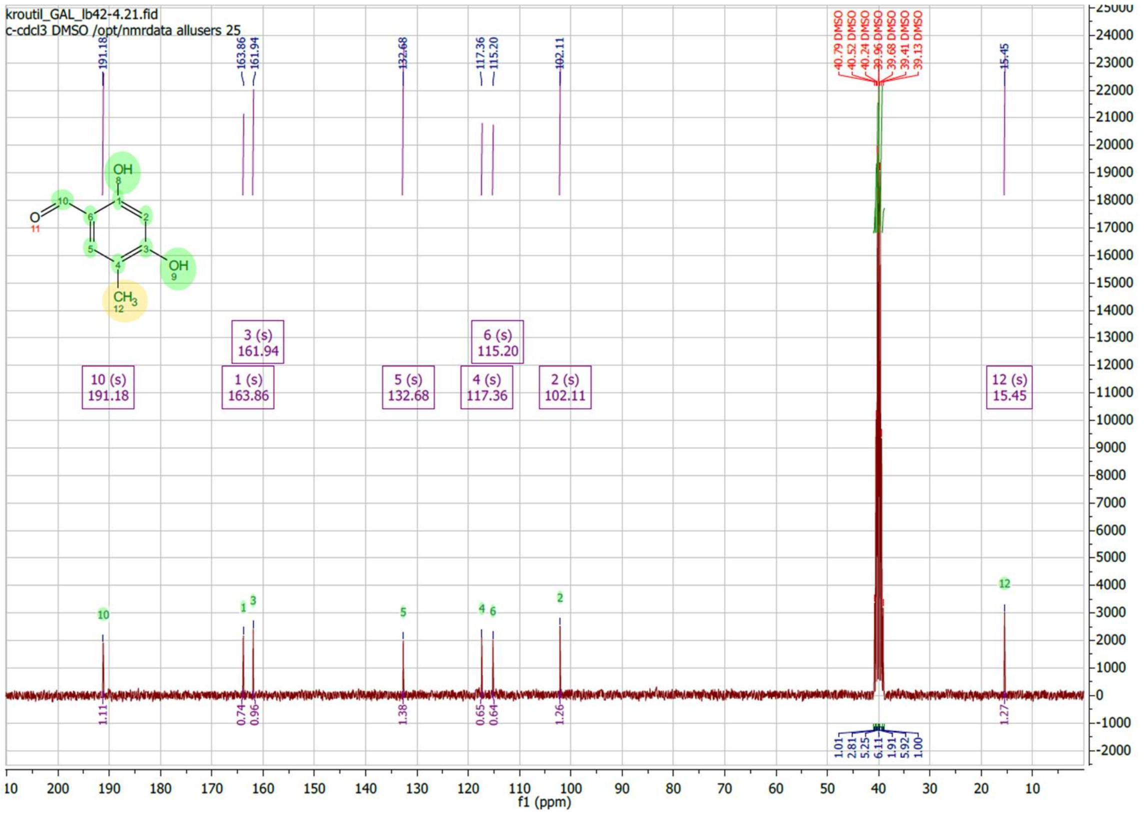

Figure S44. ^{13}C NMR spectrum of 2,4-dihydroxy-5-methylbenzaldehyde (**3b**) (solvent-DMSO-d₆, 75 MHz).

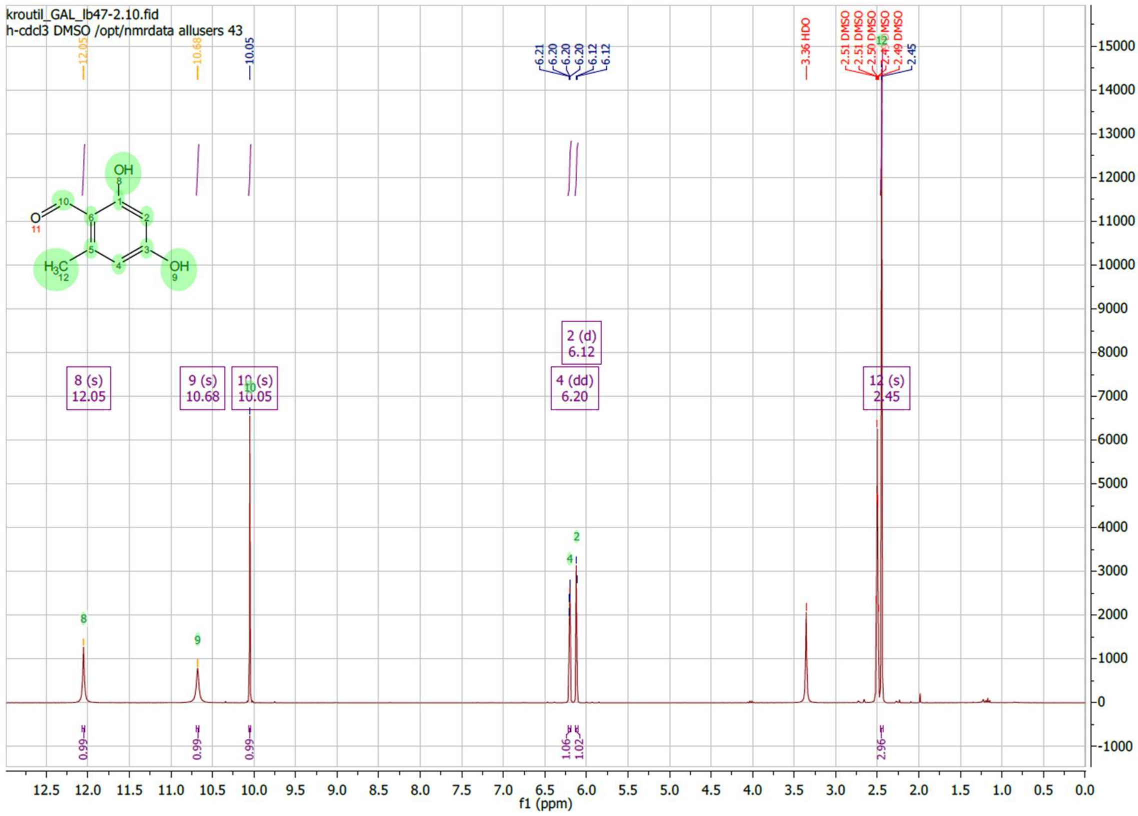

Figure S45. ^{1}H NMR spectrum of 2,4-dihydroxy-6-methylbenzaldehyde (**3c**) (solvent-DMSO-d$_6$, 300 MHz).

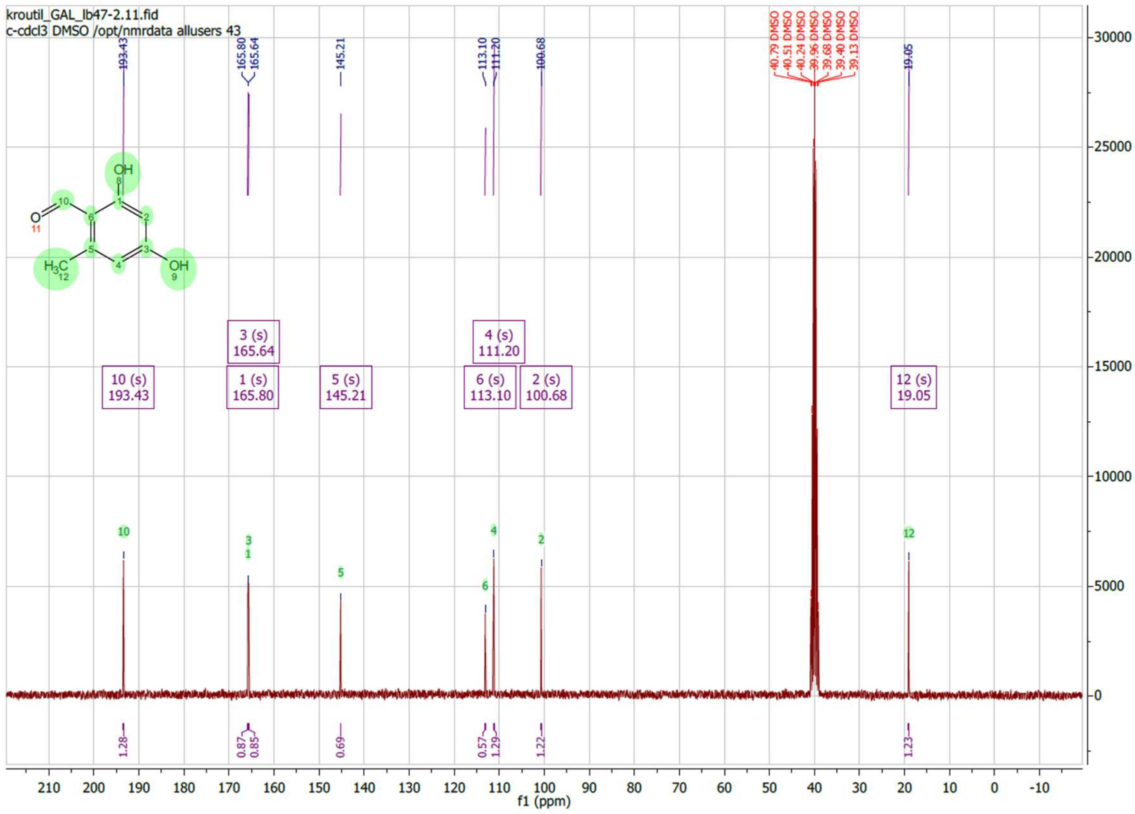

Figure S46. ^{13}C NMR spectrum of 2,4-dihydroxy-6-methylbenzaldehyde (**3c**) (solvent-DMSO-d$_6$, 75 MHz).

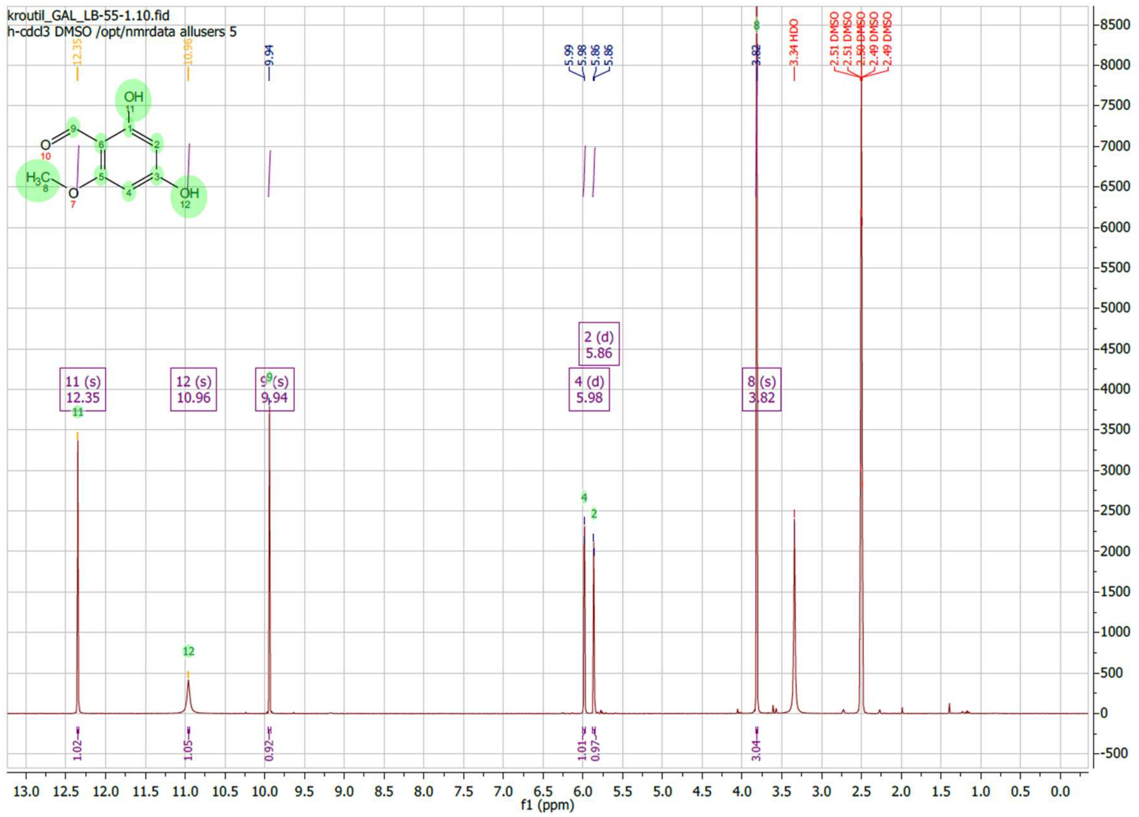

Figure S47. ^{1}H NMR spectrum of 2,4-dihydroxy-6-methoxybenzaldehyde (**3d**) (solvent-DMSO-d_6, 300 MHz).

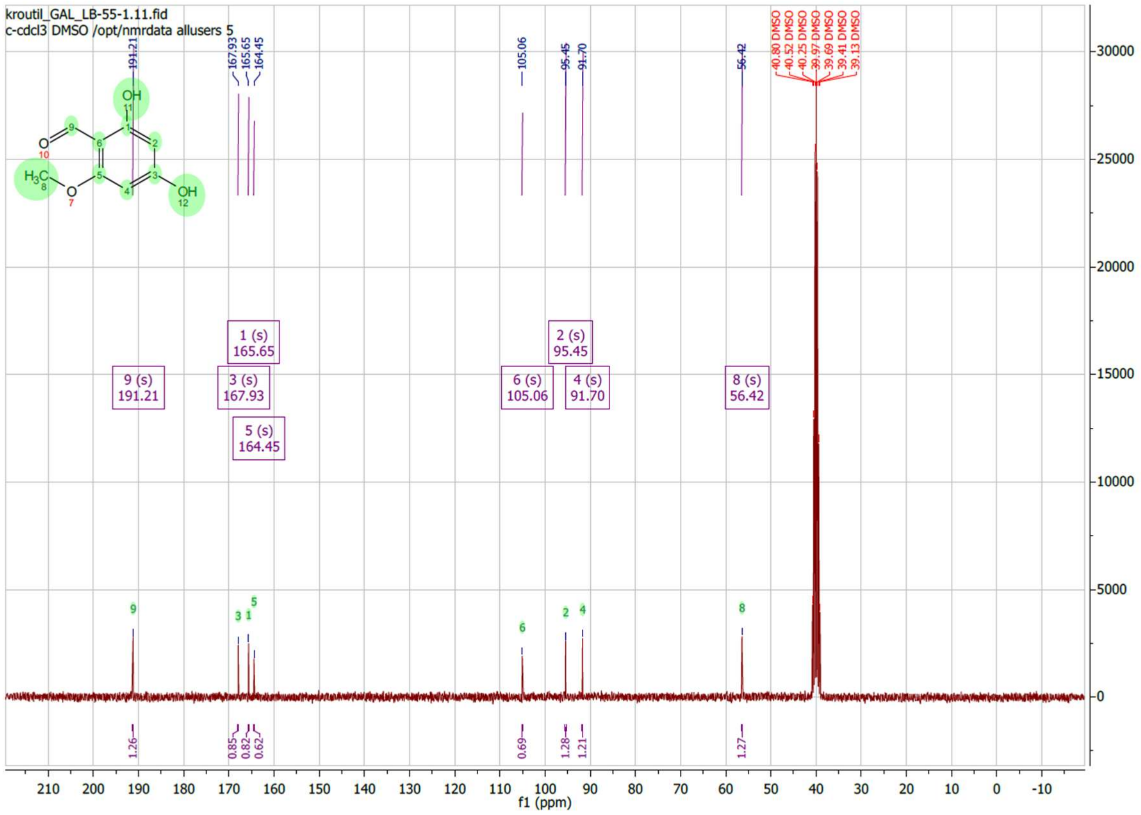

Figure S48. ^{13}C NMR spectrum of 2,4-dihydroxy-6-methoxybenzaldehyde (**3d**) (solvent-DMSO-d_6, 75 MHz).

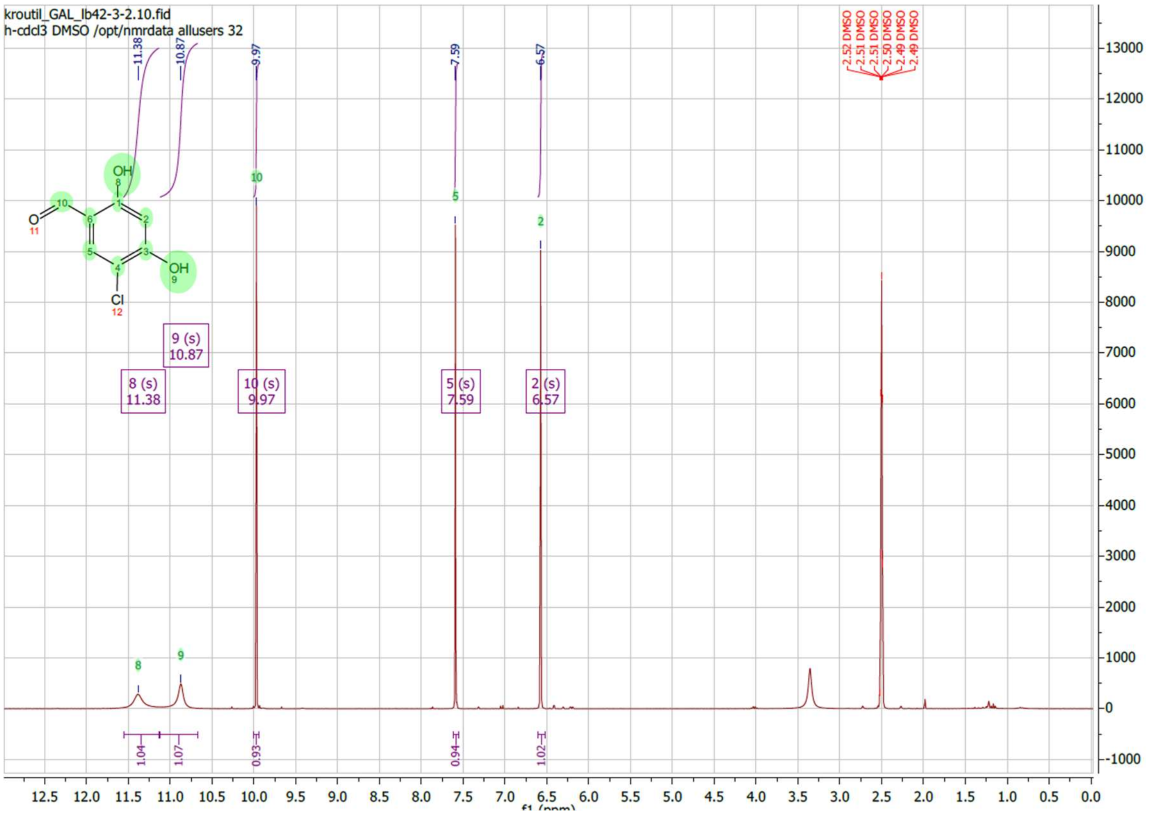

Figure S49. ^{1}H NMR spectrum of 5-chloro-2,4-dihydroxybenzaldehyde (**3e**) (solvent-DMSO-d_6, 300 MHz).

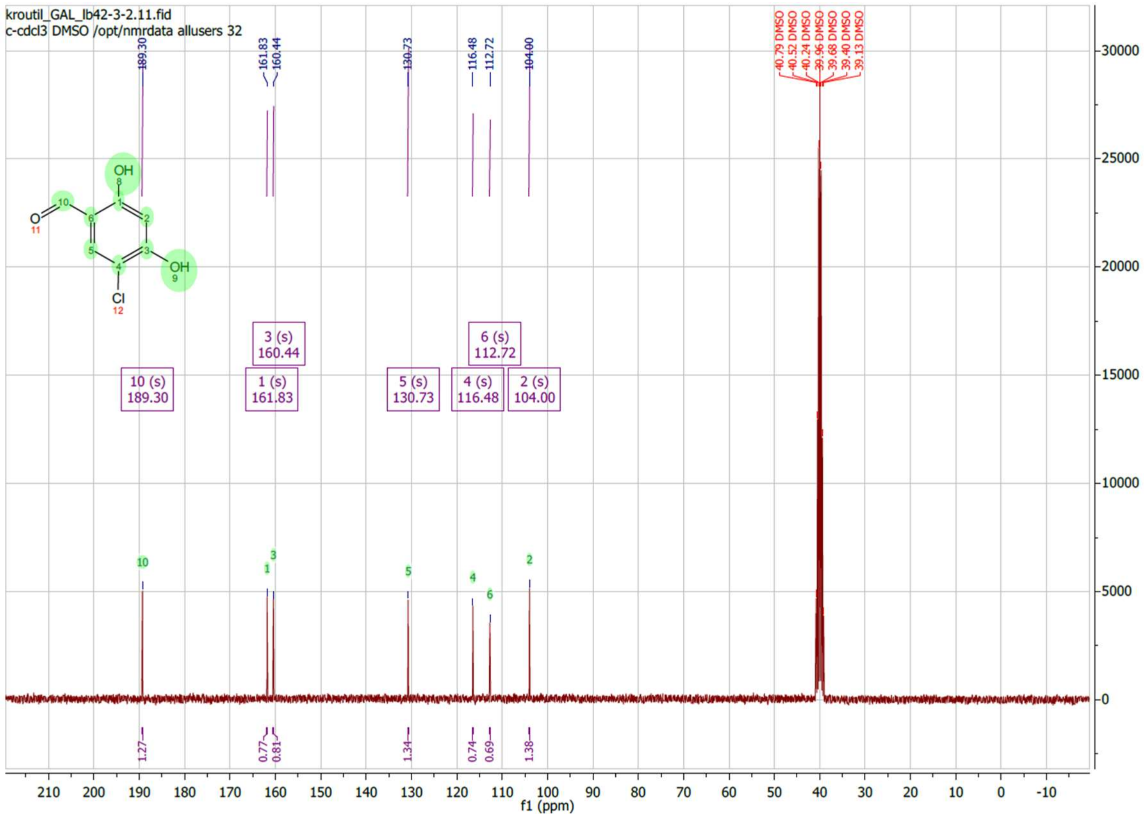

Figure S50. ^{13}C NMR spectrum of 5-chloro-2,4-dihydroxybenzaldehyde (**3e**) (solvent-DMSO-d_6, 75 MHz).

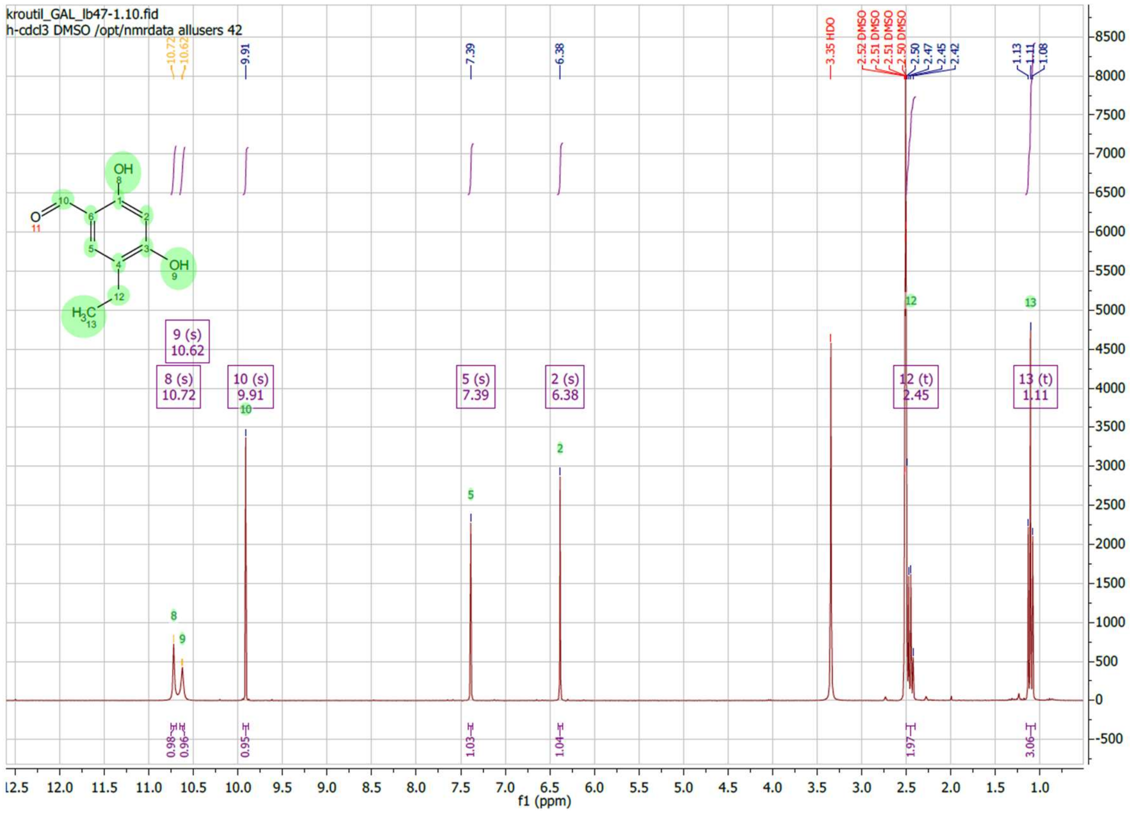

Figure S51. ^{1}H NMR spectrum of 2,4-dihydroxy-5-ethylbenzaldehyde (**3f**) (solvent-DMSO-d$_6$, 300 MHz).

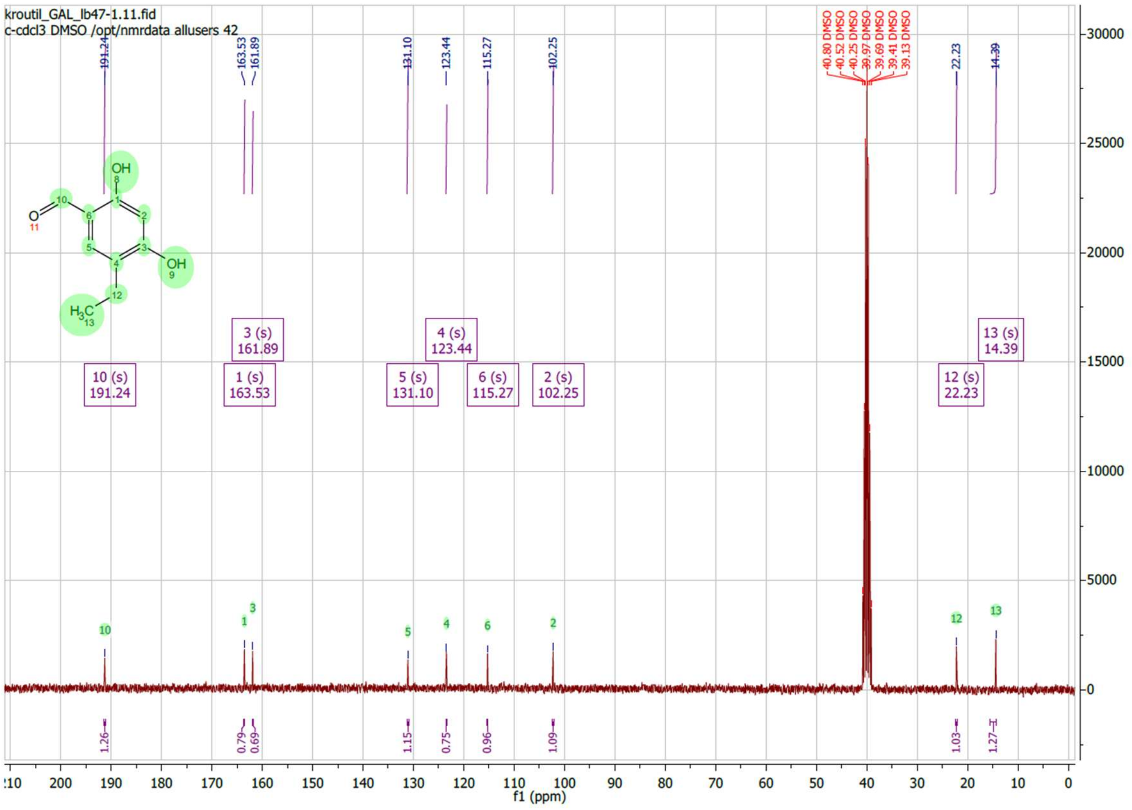

Figure S52. ^{13}C NMR spectrum of 2,4-dihydroxy-5-ethylbenzaldehyde (**3f**) (solvent-DMSO-d$_6$, 75 MHz).

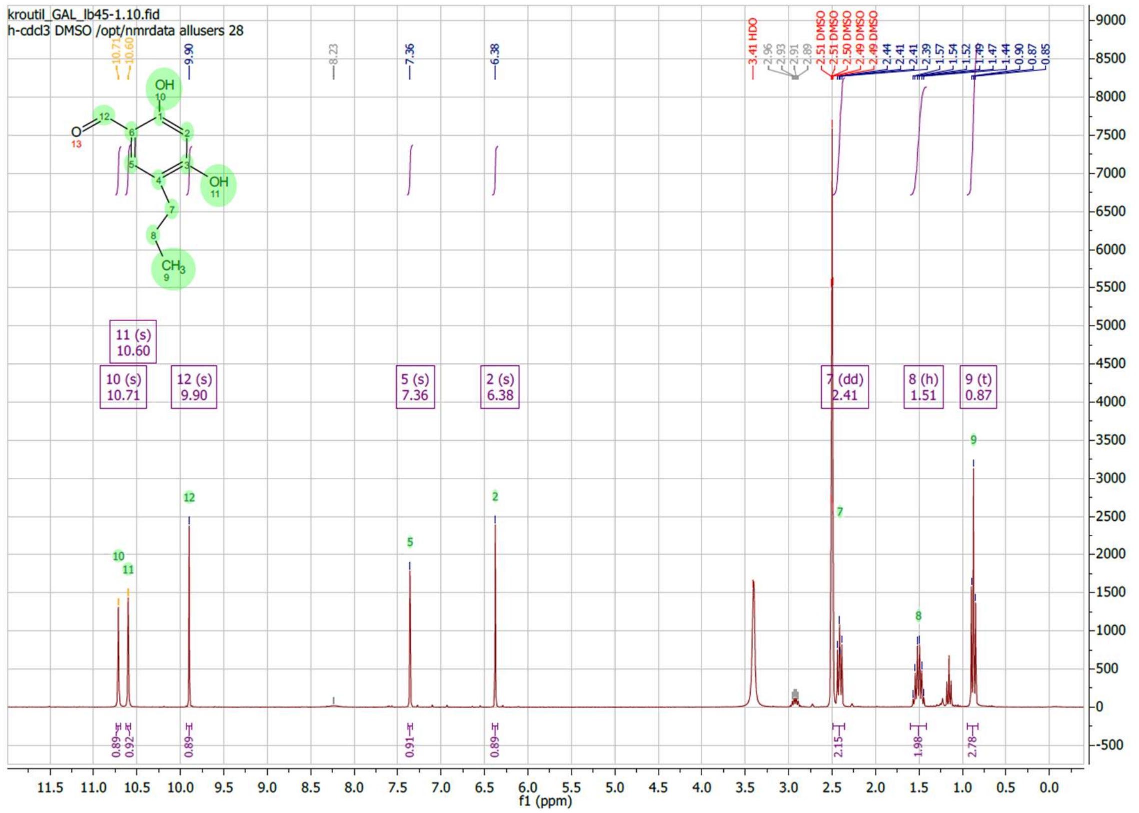

Figure S53. ^{1}H NMR spectrum of 2,4-dihydroxy-5-propylbenzaldehyde (**3g**) (solvent-DMSO-d_6, 300 MHz).

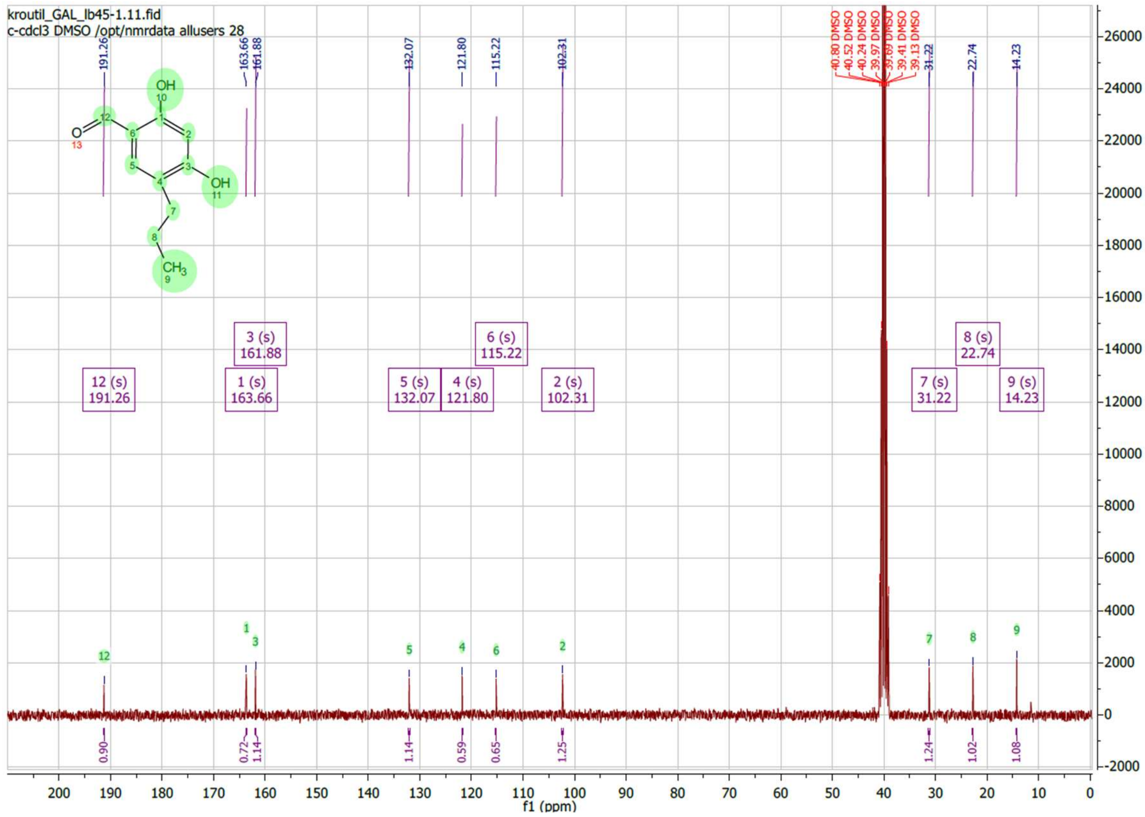

Figure S54. ^{13}C NMR spectrum of 2,4-dihydroxy-5-propylbenzaldehyde (**3g**) (solvent-DMSO-d_6, 75 MHz).

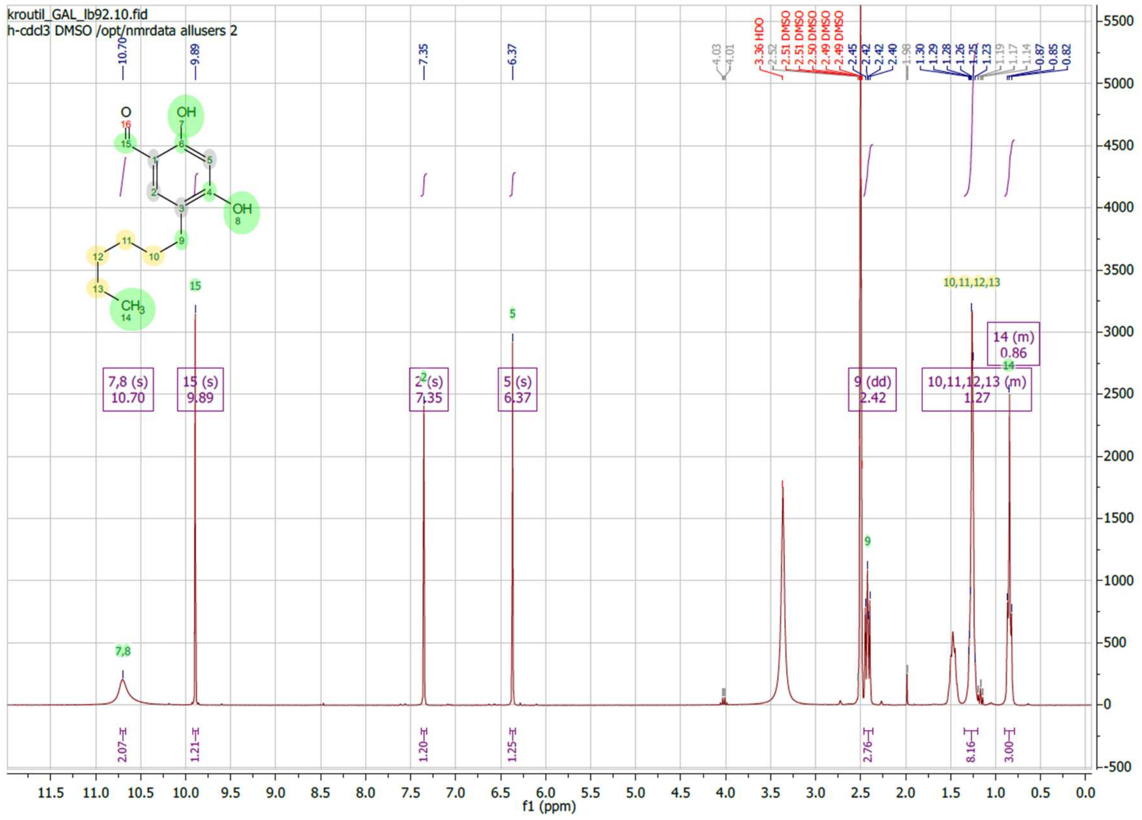

Figure S55. ^{1}H NMR spectrum of 2,4-dihydroxy-5-hexylbenzaldehyde (**3h**) (solvent-DMSO-d₆, 300 MHz).

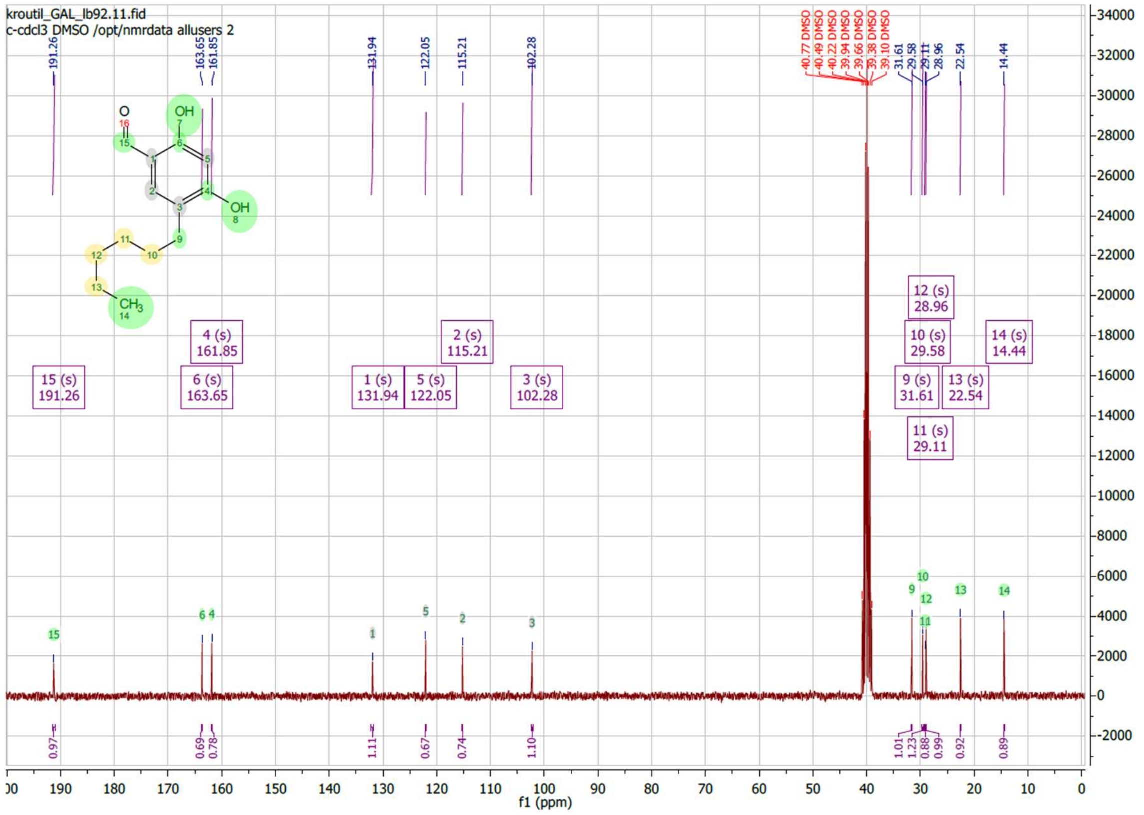

Figure S56. ^{13}C NMR spectrum of 2,4-dihydroxy-5-hexylbenzaldehyde (**3h**) (solvent-DMSO-d₆, 75 MHz).

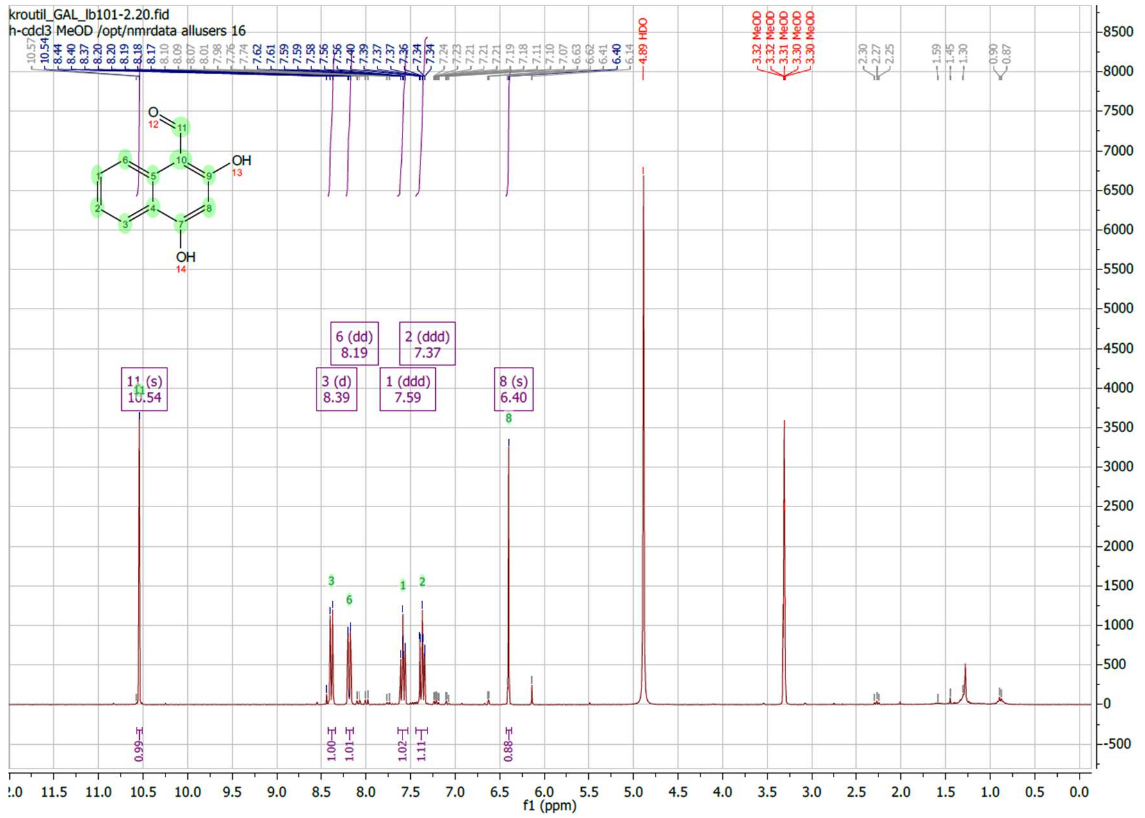

Figure S57. ^{1}H NMR spectrum of 2,4-dihydroxy-1-naphthaldehyde (**3i**) (solvent-DMSO-d₆, 300 MHz).

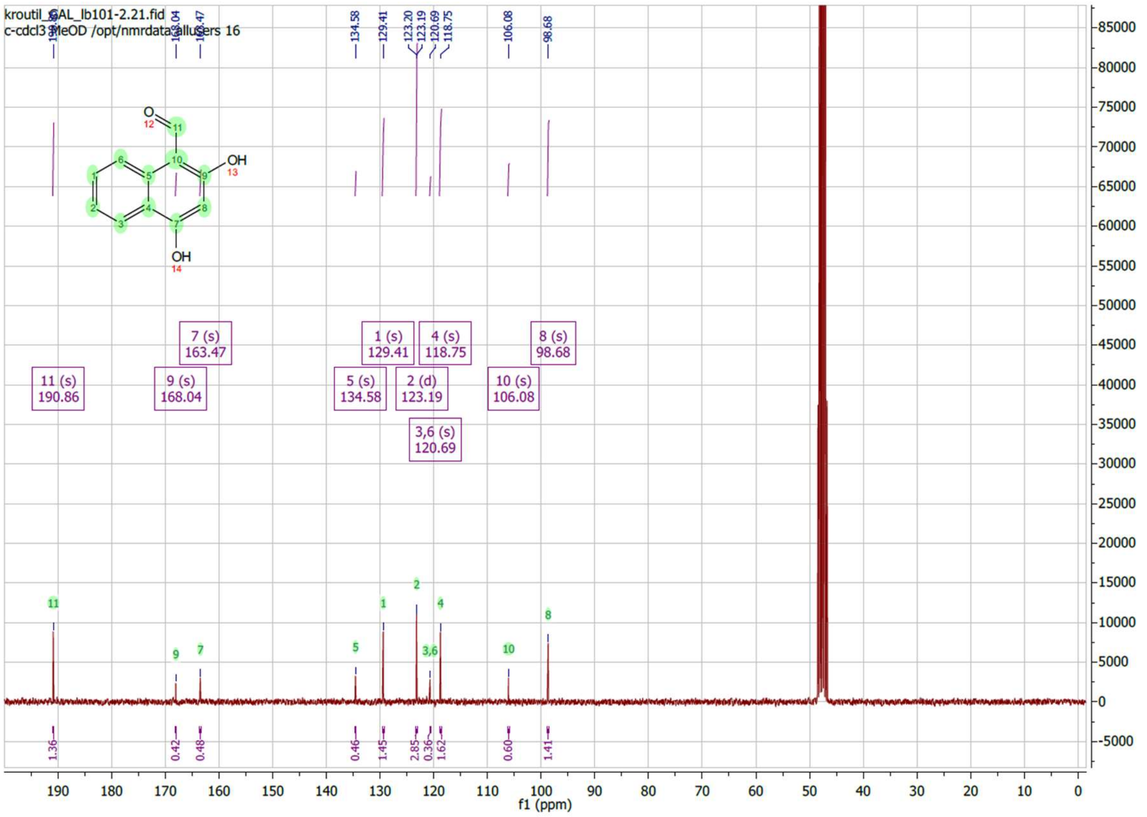

Figure S58. ^{13}C NMR spectrum of 2,4-dihydroxy-1-naphthaldehyde (**3i**) (solvent-DMSO-d₆, 75 MHz).

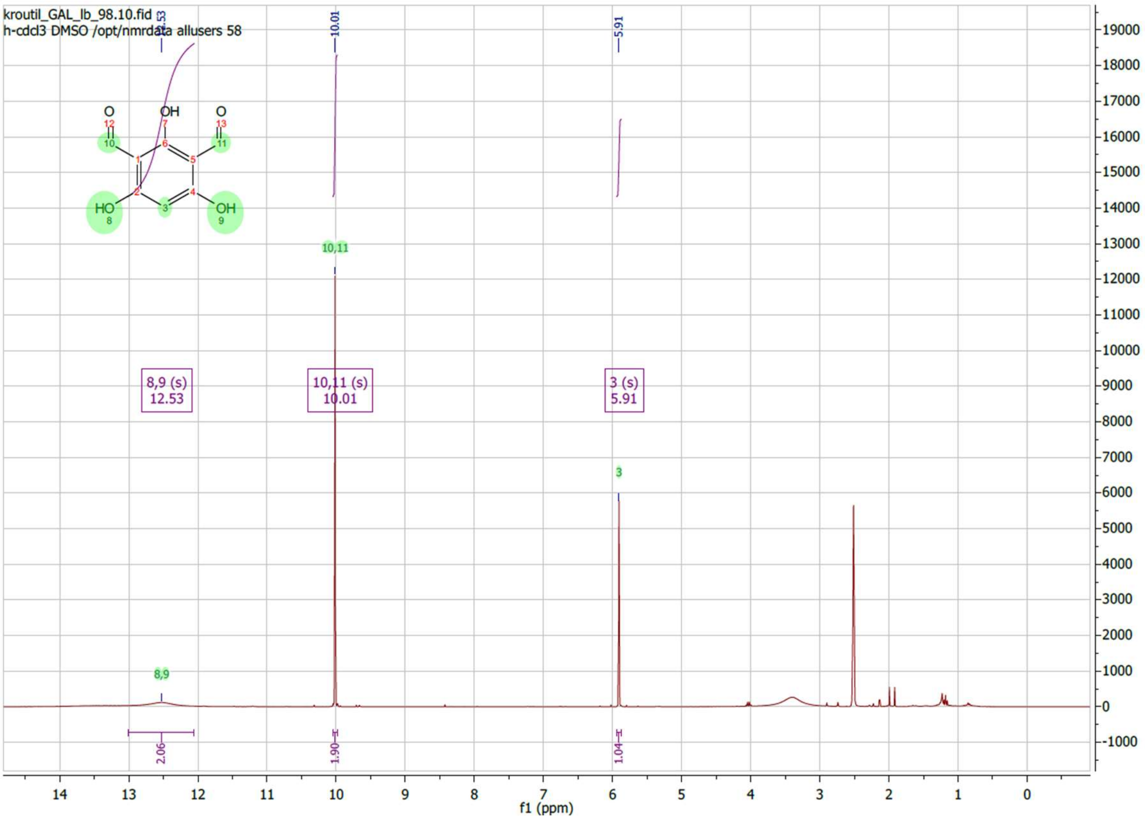

Figure S59. ^{1}H NMR spectrum of 2,4,6-trihydroxyisophthalaldehyde (**4j**) (solvent-DMSO-d_6, 300 MHz).

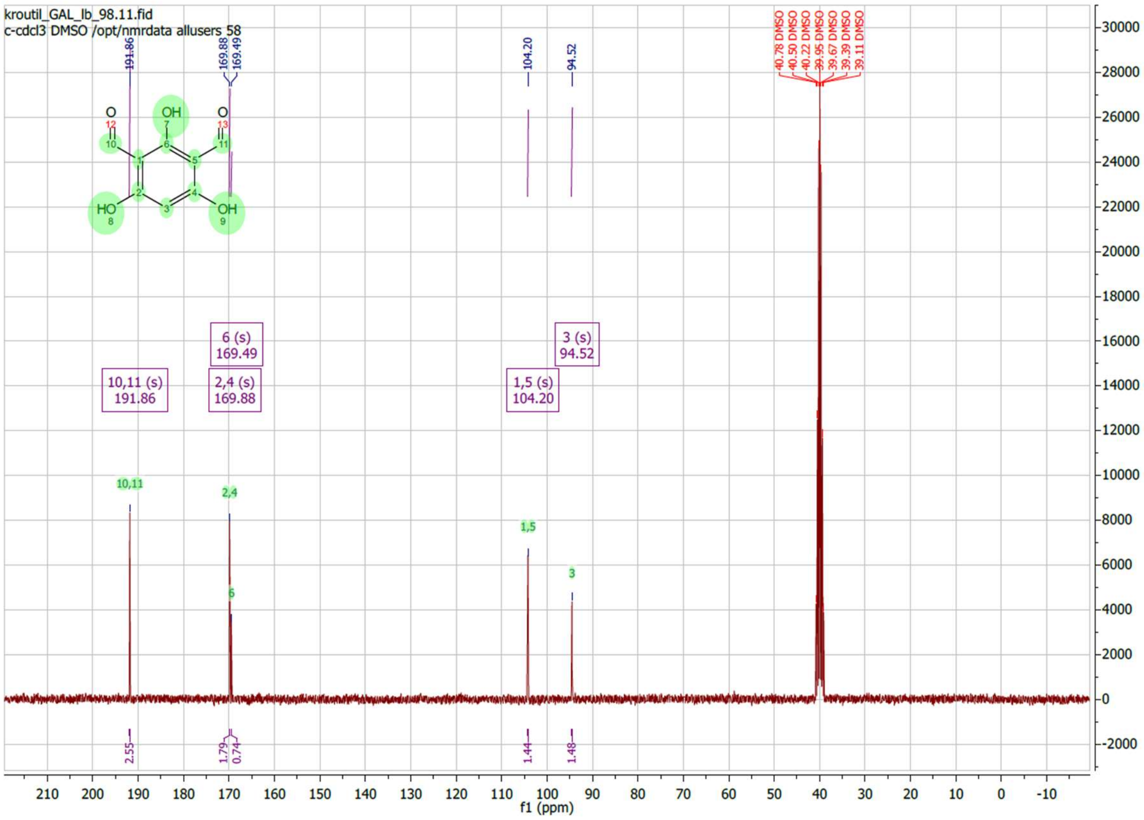

Figure S60. ^{13}C NMR spectrum of 2,4,6-trihydroxyisophthalaldehyde (**4j**) (solvent-DMSO-d_6, 75 MHz).

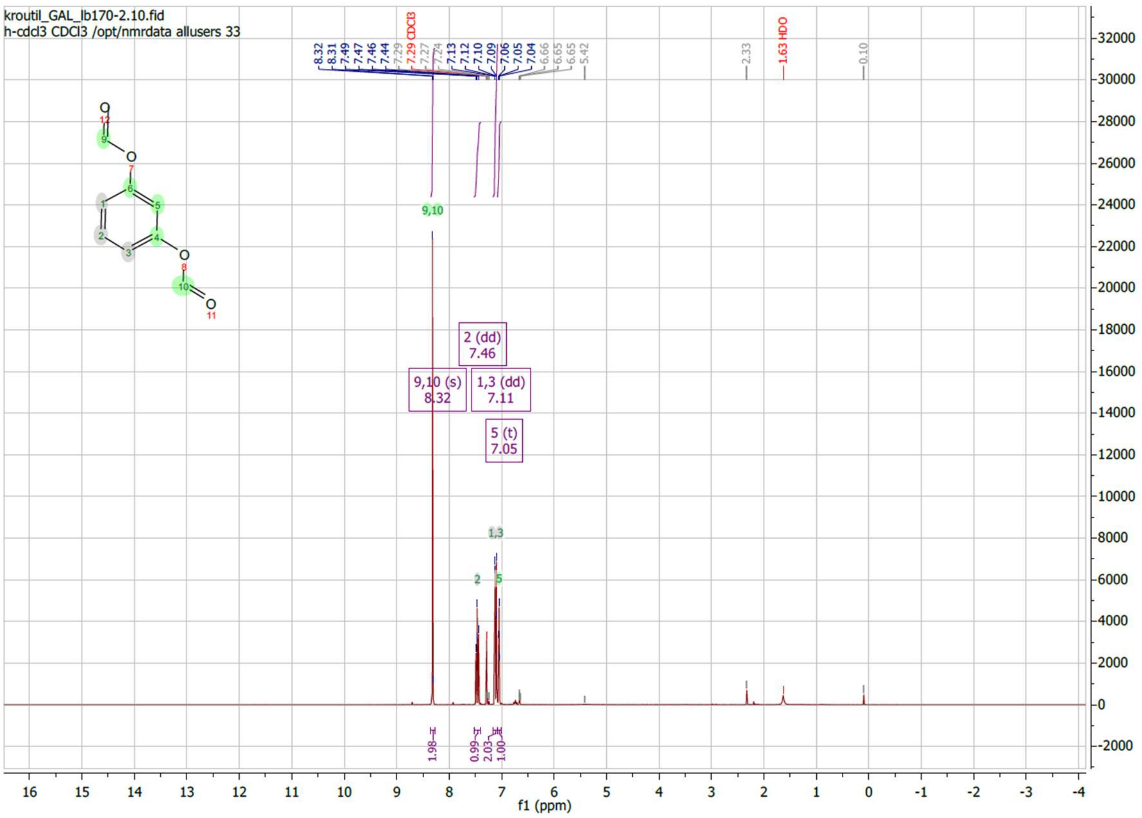

Figure S61. ^{1}H NMR spectrum of 1,3-phenylene diformate (solvent-$CDCl_3$, 300 MHz).

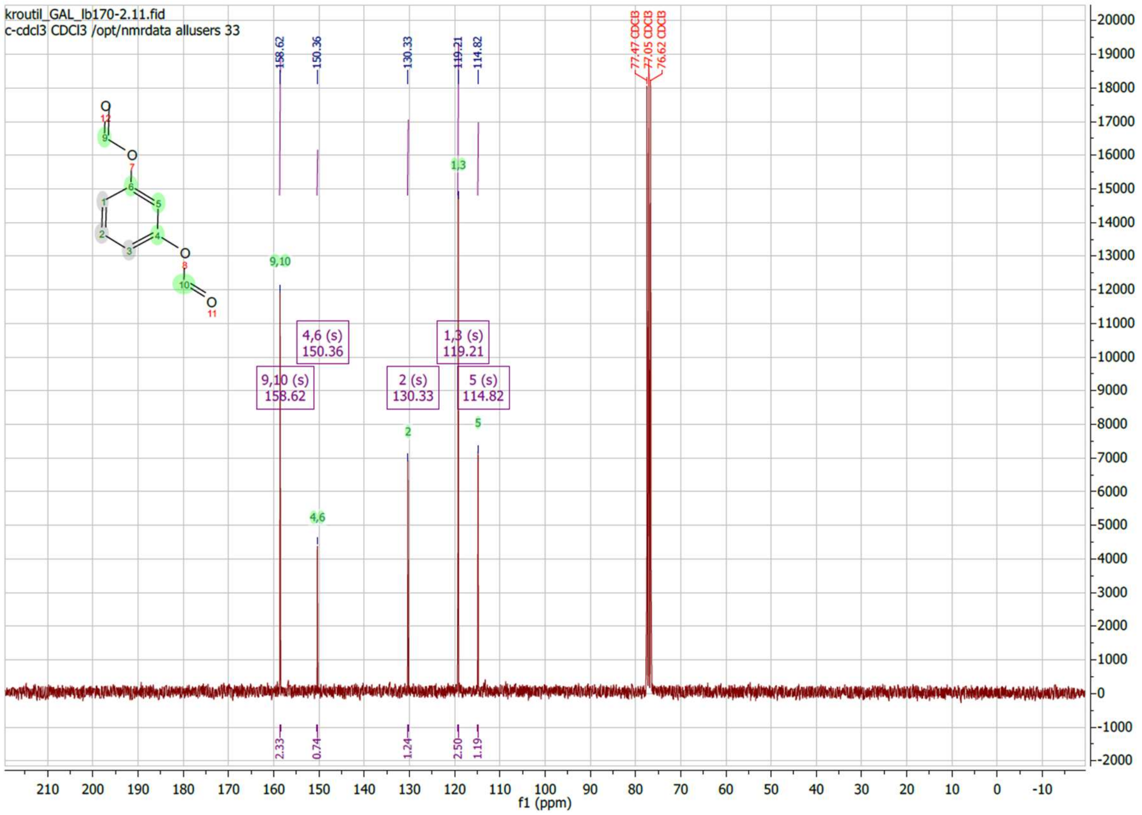

Figure S62. ^{13}C NMR spectrum of 1,3-phenylene diformate (solvent-$CDCl_3$, 75 MHz).

10. References

[1] N. G. Schmidt, T. Pavkov‐Keller, N. Richter, B. Wiltschi, K. Gruber, W. Kroutil, "Biocatalytic Friedel‐crafts acylation and fries reaction" *Angew. Chem.* **2017**, *129*, 7723-7727.

[2] B. M. Gross, M. Oestreich, "The Trityl Cation Embedded into a [7]Helicene-Like Backbone: Preparation and Application as a Lewis Acid Catalyst" *Synth.* **2021**, *53*, 2512-2516.

[3] J. Abramson, J. Adler, J. Dunger, R. Evans, T. Green, A. Pritzel, O. Ronneberger, L. Willmore, A. J. Ballard, J. Bambrick, S. W. Bodenstein, D. A. Evans, C.-C. Hung, M. O'Neill, D. Reiman, K. Tunyasuvunakool, Z. Wu, A. Žemgulytė, E. Arvaniti, C. Beattie, O. Bertolli, A. Bridgland, A. Cherepanov, M. Congreve, A. I. Cowen-Rivers, A. Cowie, M. Figurnov, F. B. Fuchs, H. Gladman, R. Jain, Y. A. Khan, C. M. R. Low, K. Perlin, A. Potapenko, P. Savy, S. Singh, A. Stecula, A. Thillaisundaram, C. Tong, S. Yakneen, E. D. Zhong, M. Zielinski, A. Žídek, V. Bapst, P. Kohli, M. Jaderberg, D. Hassabis, J. M. Jumper, "Accurate structure prediction of biomolecular interactions with AlphaFold 3" *Nature* **2024**, *630*, 493-500.

[4] D. Liebschner, P. V. Afonine, M. L. Baker, G. Bunkóczi, V. B. Chen, T. I. Croll, B. Hintze, L. W. Hung, S. Jain, A. J. McCoy, N. W. Moriarty, R. D. Oeffner, B. K. Poon, M. G. Prisant, R. J. Read, J. S. Richardson, D. C. Richardson, M. D. Sammito, O. V. Sobolev, D. H. Stockwell, T. C. Terwilliger, A. G. Urzhumtsev, L. L. Videau, C. J. Williams, P. D. Adams, "Macromolecular structure determination using X-rays, neutrons and electrons: recent developments in Phenix" *Acta Crystallogr. Sect. D. Biol. Crystallogr.* **2019**, *75*, 861-877.

[5] J. Agirre, M. Atanasova, H. Bagdonas, C. B. Ballard, A. Baslé, J. Beilsten-Edmands, R. J. Borges, D. G. Brown, J. J. Burgos-Mármol, J. M. Berrisford, P. S. Bond, I. Caballero, L. Catapano, G. Chojnowski, A. G. Cook, K. D. Cowtan, T. I. Croll, J. Debreczeni, N. E. Devenish, E. J. Dodson, T. R. Drevon, P. Emsley, G. Evans, P. R. Evans, M. Fando, J. Foadi, L. Fuentes-Montero, E. F. Garman, M. Gerstel, R. J. Gildea, K. Hatti, M. L. Hekkelman, P. Heuser, S. W. Hoh, M. A. Hough, H. T. Jenkins, E. Jiménez, R. P. Joosten, R. M. Keegan, N. Keep, E. B. Krissinel, P. Kolenko, O. Kovalevskiy, V. S. Lamzin, D. M. Lawson, A. A. Lebedev, A. G. W. Leslie, B. Lohkamp, F. Long, M. Malý, A. J. McCoy, S. J. McNicholas, A. Medina, C. Millán, J. W. Murray, G. N. Murshudov, R. A. Nicholls, M. E. M. Noble, R. Oeffner, N. S. Pannu, J. M. Parkhurst, N. Pearce, J. Pereira, A. Perrakis, H. R. Powell, R. J. Read, D. J. Rigden, W. Rochira, M. Sammito, F. Sánchez Rodríguez, G. M. Sheldrick, K. L. Shelley, F. Simkovic, A. J. Simpkin, P. Skubak, E. Sobolev, R. A. Steiner, K. Stevenson, I. Tews, J. M. H. Thomas, A. Thorn, J. T. Valls, V. Uski, I. Usón, A. Vagin, S. Velankar, M. Vollmar, H. Walden, D. Waterman, K. S. Wilson, M. D. Winn, G. Winter, M. Wojdyr, K. Yamashita, "The CCP4 suite: integrative software for macromolecular crystallography" *Acta Crystallogr. Sect. D. Biol. Crystallogr.* **2023**, *79*, 449-461.

[6] C. J. Williams, J. J. Headd, N. W. Moriarty, M. G. Prisant, L. L. Videau, L. N. Deis, V. Verma, D. A. Keedy, B. J. Hintze, V. B. Chen, S. Jain, S. M. Lewis, W. B. Arendall, 3rd, J. Snoeyink, P. D. Adams, S. C. Lovell, J. S. Richardson, D. C. Richardson, "MolProbity: More and better reference data for improved all-atom structure validation" *Protein Sci.* **2018**, *27*, 293-315.

[7] Schrödinger LLC, **2015**.

[8] T. Pavkov-Keller, N. G. Schmidt, A. Żądło-Dobrowolska, W. Kroutil, K. Gruber, "Structure and Catalytic Mechanism of a Bacterial Friedel–Crafts Acylase" *ChemBioChem* **2019**, *20*, 88-95.

[9] F. Mohamadi, N. G. J. Richards, W. C. Guida, R. Liskamp, M. Lipton, C. Caufield, G. Chang, T. Hendrickson, W. C. Still, "Macromodel—an integrated software system for modeling organic and bioorganic molecules using molecular mechanics" *J. Comput. Chem.* **1990**, *11*, 440-467.

[10] K. S. Watts, P. Dalal, A. J. Tebben, D. L. Cheney, J. C. Shelley, "Macrocycle Conformational Sampling with MacroModel" *J. Chem. Inf. Model.* **2014**, *54*, 2680-2696.

[11] G. Madhavi Sastry, M. Adzhigirey, T. Day, R. Annabhimoju, W. Sherman, "Protein and ligand preparation: parameters, protocols, and influence on virtual screening enrichments" *J. Comput. Aided Mol. Des.* **2013**, *27*, 221-234.

[12] Y. Yang, K. Yao, M. P. Repasky, K. Leswing, R. Abel, B. K. Shoichet, S. V. Jerome, "Efficient Exploration of Chemical Space with Docking and Deep Learning" *J. Chem. Theory Comput.* **2021**, *17*, 7106-7119.

[13] R. A. Friesner, R. B. Murphy, M. P. Repasky, L. L. Frye, J. R. Greenwood, T. A. Halgren, P. C. Sanschagrin, D. T. Mainz, "Extra Precision Glide: Docking and Scoring Incorporating a Model of Hydrophobic Enclosure for Protein−Ligand Complexes" *J. Med. Chem.* **2006**, *49*, 6177-6196.

[14] T. A. Halgren, R. B. Murphy, R. A. Friesner, H. S. Beard, L. L. Frye, W. T. Pollard, J. L. Banks, "Glide: A New Approach for Rapid, Accurate Docking and Scoring. 2. Enrichment Factors in Database Screening" *J. Med. Chem.* **2004**, *47*, 1750-1759.

[15] R. A. Friesner, J. L. Banks, R. B. Murphy, T. A. Halgren, J. J. Klicic, D. T. Mainz, M. P. Repasky, E. H. Knoll, M. Shelley, J. K. Perry, D. E. Shaw, P. Francis, P. S. Shenkin, "Glide: A New Approach for Rapid, Accurate Docking and Scoring. 1. Method and Assessment of Docking Accuracy" *J. Med. Chem.* **2004**, *47*, 1739-1749.
